# The plasma metabolome of juvenile idiopathic arthritis varies according to subtype and underlying inflammatory status

**DOI:** 10.1186/s12969-024-01041-8

**Published:** 2024-12-30

**Authors:** Jooa Kwon, Melanie R. Neeland, Justine A. Ellis, Jane Munro, Richard Saffery, Boris Novakovic, Toby Mansell

**Affiliations:** 1 Infection, Immunity and Global Health Theme, Murdoch Children’s Research Institute, Parkville, VIC 3052 Australia; 2 Department of Paediatrics, University of Melbourne, Parkville, VIC 3052 Australia; 3 Northern Health Research Development and Governance Unit, Epping, VIC 3076 Australia; 4 School of Health & Biomedical Sciences, RMIT University, Melbourne, VIC 3000 Australia; 5 Royal Children’s Hospital Melbourne, Parkville, VIC 3052 Australia

**Keywords:** Juvenile idiopathic arthritis, JIA, Systemic JIA, Polyarticular, Rheumatoid factor, NMR metabolomics, Glycoprotein acetyls, GlycA, High-sensitivity C-Reactive protein, Inflammation, Metabolome

## Abstract

**Background:**

Juvenile idiopathic arthritis (JIA) is challenging to classify and effectively monitor due to the lack of disease- and subtype-specific biomarkers. A robust molecular signature that tracks with specific JIA features over time is urgently required, and targeted plasma metabolomics may reveal such a signature. The primary aim of this study was to characterise the differences in the plasma metabolome between JIA patients and non-JIA controls and identify specific markers of JIA subtype. We also assessed the extent to which these signatures are due to underlying inflammation as assessed by glycoprotein acetyls (GlycA) and high-sensitivity C-Reactive Protein (hsCRP) levels.

**Methods:**

Targeted nuclear magnetic resonance (NMR) metabolomic profiles of plasma of 72 children with JIA and 18 controls were assessed cross-sectionally. Associations between 71 metabolomic biomarkers and JIA, JIA subtype, disease activity status, and inflammation markers (GlycA and hsCRP) were assessed using multivariable linear regression models.

**Results:**

JIA was associated with higher GlycA (mean difference = 0.93 standard deviations, 95% confidence interval = [0.370, 1.494], *P*_*adj*_ = 0.039) and docosahexaenoic acid (1.06, [0.51, 1.60], *P*_*adj*_ = 0.021), and lower acetate (-0.92, [-1.43, -0.41], *P*_*adj*_ = 0.024) relative to controls. This variation was largely driven by systemic JIA (sJIA), with 24 of 71 total biomarkers significantly different (*P*_*adj*_ <0.05) relative to controls. There were no specific differences identified in oligoarticular (oJIA) or polyarticular (rheumatoid factor positive or negative) JIA relative to controls. Despite being generally highly correlated with hsCRP (*r* > 0.70), GlycA, but not hsCRP, was positively associated with active disease in sJIA (0.22, [-0.40, -0.04], P_*adj*_ = 0.018), and 6 of 24 sJIA-associated markers were associated with GlycA levels. Only 1 sJIA-associated biomarker, histidine, was associated with hsCRP levels.

**Conclusion:**

Differences in the plasma NMR metabolomic profiles are apparent in children with sJIA, but not other JIA subtypes, relative to non-JIA controls. These findings suggest a potential utility for classifying and monitoring JIA through metabolomic profiling, with chronic inflammation, measured by GlycA, potentially playing a role in at least some of these metabolomic differences.

**Supplementary Information:**

The online version contains supplementary material available at 10.1186/s12969-024-01041-8.

## Key messages

• Children with JIA showed limited metabolomic differences in plasma compared to non-JIA controls.

• Subtype-associated analysis identified exclusive metabolomic variation in sJIA.

• Some metabolomic differences in sJIA may be driven by chronic inflammation.

• GlycA may better reflect disease activity and metabolomic differences in sJIA than other inflammatory markers such as hsCRP.

**Supplementary Information:**

The online version contains supplementary material available at 10.1186/s12969-024-01041-8.

## Introduction

Juvenile idiopathic arthritis (JIA) is a heterogenous group of chronic rheumatic diseases in children, categorised into subtypes such as oligoarticular (oJIA, 27–60%), polyarticular (pJIA, 2–30%, Rheumatoid factor-negative more common than Rheumatoid factor-positive) and systemic JIA (sJIA, 10–20%), based on numbers of affected joints and presence of Rheumatoid factor (RF) and systemic symptoms [1–4]. The response rates of drug treatment between JIA subtypes vary, and there is a lack of specific biomarkers to distinguish JIA subtypes and to monitor disease activity. A key feature of JIA is inflammation, with certain subtypes distinguished by whether the inflammation is systemic affecting multiple organs or localized to the joints and surrounding tissues [1, 2, 5–7]. The absence of distinctive JIA-associated molecules hinders effective disease classification and monitoring [4, 6]. As such, approaches for profiling the molecular signatures of JIA and its subtypes are becoming acknowledged as a potential way to achieve more personalised treatment, and therefore improve clinical outcomes [4–6].

The plasma metabolome is a functional readout of metabolic activities in the body. Alterations in the plasma metabolome have previously been associated with chronic inflammatory conditions in childhood such as Inflammatory Bowel Disease (IBD) [8] and juvenile Systemic Lupus Erythematosus (jSLE) [9]. The metabolomic differences in adults with RA and children with jSLE relative to controls have also been characterised, some of which may promote inflammation [9, 10]. While several studies have begun to characterise the metabolome in JIA [11–13], these have yet to identify subtype-differentiating signatures or markers predictive of disease course and long-term outcome.

Untargeted metabolomics measures a wide spectrum of metabolites in a sample without prior knowledge of their identity [14]. However, due to a wide variety of metabolites, including uncharacterised molecules, it can require extensive validation to confirm their identities [14, 15]. Also, it is a relative quantification of metabolites, which could be less suitable for precise measurements for JIA subtype categorisation and monitoring [14]. In contrast, targeted metabolomics focuses on quantifying specific, predefined metabolites of interest within a sample, providing absolute concentrations [14]. Nuclear magnetic resonance (NMR)-based targeted metabolomics is increasingly recognised as having utility in identifying disease biomarkers across a range of conditions including adult RA [10, 16–19], and providing insight into disease processes [16]. Furthermore, several NMR-based metabolomic markers are now approved for clinical use in a range of settings [20].

Among the biomarkers identified through NMR-based approaches, glycoprotein acetyls (GlycA) is a promising marker of cumulative inflammation, based on glycosylation of several acute-phase proteins across multiple inflammatory pathways [21–23]. It is more stable over time and thought to better reflect chronic inflammation than conventional inflammatory markers such as high sensitivity C-reactive protein (hsCRP) or erythrocyte sedimentation rate (ESR) [21, 22, 24]. Studies in adult RA have shown that GlycA is closely associated with disease activity as well as hsCRP and ESR, which are widely used markers for monitoring disease activity in RA [25, 26].

Here, we characterised NMR metabolomic biomarkers in plasma of children with four JIA subtypes, oJIA, pJIA(RF-), pJIA(RF+), and sJIA, with the aim of characterising molecular signatures of JIA generally, and those associated with specific JIA subtypes. We also investigated the relationship between disease activity status, inflammation measured by GlycA and hsCRP, and these metabolomic differences.

## Methods

### Participants

A total of 72 children diagnosed with JIA and 18 hospital controls were recruited between 2013 and 2017 as part of the CLARITY (ChiLdhood Arthritis Risk factor Identification sTudY) cohort and biobank (Table 1) [27]. CLARITY is a cross-sectional biobank of JIA and healthy control children, which aims to investigate both genetic and environmental risk factors that impact on JIA risk. All CLARITY protocols were approved by the Human Research Ethics Committee of the Royal Children’s Hospital, Melbourne, Australia (HREC no. 27127Q) [27]. All participants provided informed consent [27]. All JIA cases (≤ 18 years) were recruited from the Royal Children’s Hospital (RCH) and diagnosed with JIA by a paediatric rheumatologist by ILAR classification [3, 27]. Active disease in cases was defined as the presence of at least one active joint or systemic features and inactive disease was assessed using the Wallace criteria as no active disease for at least 6 months on treatment or 12 months off treatment [28]. Controls (≤ 9 years) were recruited from the Day Surgery Unit and consisted of children undergoing minor surgery unrelated to immune-mediated inflammatory conditions.

**Table 1:** Baseline demographics and clinical characteristics of patients (*n* = 90)^a^

Subtypes	Age (years) *P*-value < 0.01^b^	Females (%) *P*-value < 0.05^b^	Disease activity (%)		
			Active	Inactive	Unknown
Control (*n* = 18)	6.5 (4.5–8.6)	50% (9)	NA	NA	NA
oJIA (*n* = 19)	10.5 (4.6–14.8)	53% (9)	53% (10)	16% (3)	31% (6)
RF-pJIA (*n* = 17)	9.6 (3.8–13.8)	50% (9)	76% (13)	6% (1)	18% (3)
RF + pJIA (*n* = 14)	13.7 (5.6–17.3)	100% (14)	64% (9)	7% (1)	29% (4)
sJIA (*n* = 22)	10.8 (1.8–17.8)	64% (14)	50% (11)	37% (8)	13% (3)

^a^Data are presented as mean (age range: from the youngest to the oldest) for continuous data and percentage (number) for categorical variables. Most of the cohort JIA participants received multiple medications

^b^ANOVA test between 5 groups

### NMR targeted metabolomics

We used a high-throughput proton NMR metabolomics platform (Nightingale Health, Helsinki, Finland) to quantify metabolites within plasma (https://research.nightingalehealth.com/). The platform generates 249 total measures including 39 clinically validated blood biomarkers for lipid and glucose metabolism, ketone bodies, amino acids and a marker of chronic inflammation (GlycA) [20, 21]. Plasma samples (EDTA tube collection) from the 90 participants were shipped on dry ice to Nightingale Health (Helsinki, Finland) for metabolomic quantification.

### Preparation of data

Due to the high degree of correlation between markers (natural unit Spearman’s rank correlation) (Supplementary Figure S1), 249 initial measures (Supplementary Data D1) were filtered down to 71 primary measures prior to statistical analysis. Consistent with previous studies with this metabolomics platform [29–31], lipoprotein subclasses, relative lipoprotein lipid concentrations groups, and ratio measures expressed as a percentage were removed due to their high correlation with other measures. Data for 3-hydroxybutyrate were also removed due to the high percentage (55%) of missing data. Since the fasting status of participants was unknown, fasting-sensitive measures including glucose and lactate were also removed prior to statistical analysis.

### Statistical analysis

All analyses were conducted using *R* (version 3.6.3). Metabolomic measures were natural log-transformed and scaled to a standard distribution, with effect sizes reported in standard deviation units. Linear regression models adjusted for age and sex were used to identify (i) JIA-associated biomarkers independent of subtypes, (ii) subtype-associated biomarkers, and (iii) inflammation (hsCRP and GlycA) associated biomarkers. Discovery regression models from the *ggforestplot* package were used for analysis. For JIA-associated biomarkers, disease status (control group (0) and JIA group (1) was used as a predictor. Estimates were reported as an adjusted mean difference (AMD) and the associated 95% confidence interval (CI). To control the false discovery rate (FDR), we applied the Benjamini-Hochberg (BH) procedure [32] with *p*_*adj*_ values below 0.05 considered statistically significant. This corresponds to a stringency level of 5% for the false discover rate (FDR). Volcano and forest plots were generated using *ggplot2*. Biomarkers of interest were visualised using box-and-whisker plots. In secondary analyses, we considered models stratifying participants by disease activity status (active disease only and inactive disease only models). For subtype-associated biomarkers, each subtype [sJIA, oJIA, pJIA(RF+) and pJIA(RF-)] was independently compared to the control group using the same approach as above. For inflammation analyses, we first investigated whether there were differences in GlycA and hsCRP levels between disease status (inactive, active and unknown) in JIA cases and within each subtype using pairwise Student’s t-tests. We also calculated the Pearson’s correlation between GlycA and hsCRP for each subtype. We investigated inflammation-associated metabolomic differences using linear regression modelling with GlycA or hsCRP as the exposure and each of the 70 non-GlycA metabolomic measures as the outcome, adjusted for age and sex. Biomarkers of interest were visualised with scatter plots with linear fit lines.

### Data availability

Mean metabolomic biomarker levels per group for 71 primary measures are provided in Supplementary data D2. Individual participant data from CLARITY cannot be made freely available online per the approved ethics for this study. Interested parties can access the data used in this study upon reasonable request, with approval by the CLARITY data custodians.

## Results

### Children with JIA show limited variation in the plasma metabolome relative to non-JIA controls

**Fig. 1:**
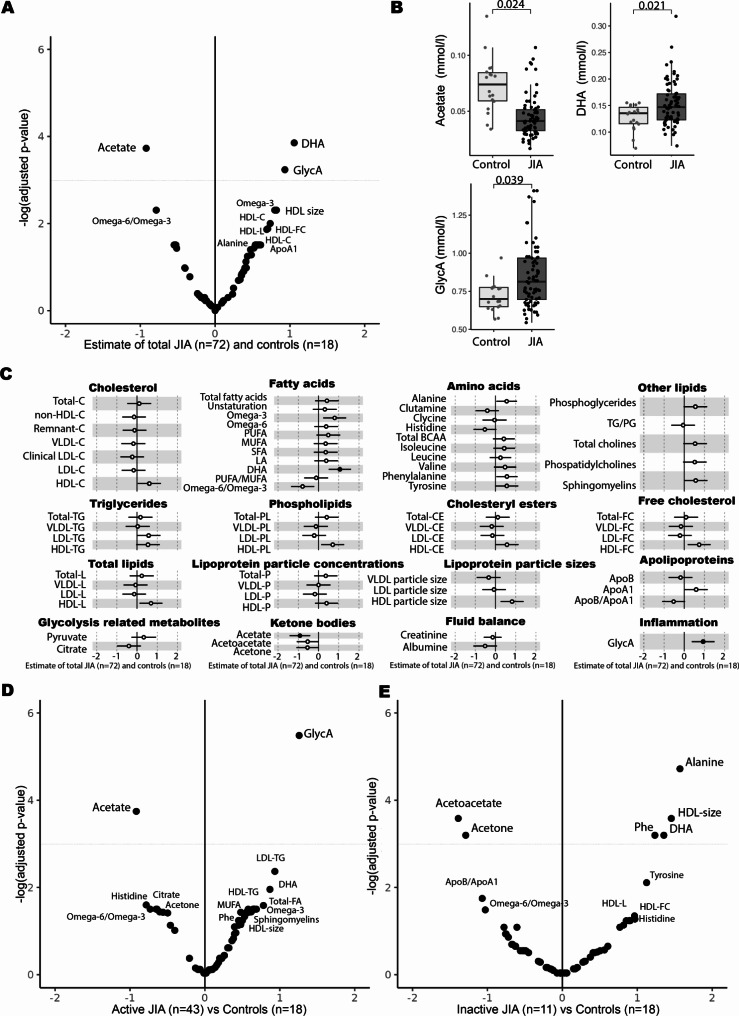
JIA-associated metabolomic biomarkers **(A)** Volcano plot for the estimated metabolomic difference between JIA group (*n* = 72) and controls (*n* = 18) from adjusted linear regressions model. A dotted line indicates *p*_*adj*_ value cut-off of 0.05. Biomarkers with *p*_*adj*_<0.05 are labelled above the dotted line. Biomarkers with raw *p* < 0.05 are labelled below the dotted line. **(B)** Dot plots of JIA-associated markers between JIA and non-JIA controls. The statistical comparison was performed with the Student’s T-test. *p*_*adj*_ values are marked above dendrogram. All models were adjusted for age and sex. The distribution difference between JIA group and controls for all primary metabolomic measures are shown in Supplementary Figure S2. **(C)** Forest plot for the estimated metabolomic difference between JIA group and control (circle points) from adjusted linear regression models. Error bars are 95% confidence intervals. Closed points represent *p*_*adj*_ values < 0.05 (BH). **(D)** Volcano plot for the estimated metabolomic difference between active JIA group (*n* = 43) and controls (*n* = 18) from adjusted linear regressions model. A dotted line indicates *p*_*adj*_ value cut-off of 0.05. Biomarkers with *p*_*adj*_<0.05 are labelled above the dotted line. Biomarkers with raw *p* < 0.05 are labelled below the dotted line. **(E)** Volcano plot for the estimated metabolomic difference between inactive JIA group (*n* = 13) and controls (*n* = 18) from adjusted linear regressions model. A dotted line indicates *p*_*adj*_ value cut-off of 0.05. Biomarkers with *p*_*adj*_<0.05 are labelled above the dotted line. Biomarkers with raw *p* < 0.05 are labelled below the dotted line

Linear regression was performed to identify differences in metabolomic profiles between the JIA and control groups, independently of subtype. Compared to controls, JIA status was associated with higher GlycA (mean difference = 0.93 standard deviations, 95% confidence interval = [0.370, 1.49], *P*_*adj*_ =0.039) and DHA (1.06, [0.51, 1.6], *P*_*adj*_ =0.021), and lower acetate (0.92, [-1.43, -0.41], *P*_*adj*_ =0.024) (Fig. 1A **&** Supplementary Data D3). Acetate showed the clearest separation between the JIA and control groups with regards to interquartile range (Fig. 1B). Weak associations were found for several other biomarkers, particularly amino acids and high-density lipoprotein (HDL), however, these differences were not statistically significant following correction for multiple testing (P_*adj*_ >0.05; Fig. 1C, Supplementary Data D3). In stratified models limited to active JIA patients only, the difference in GlycA was more marked (1.26, [0.69, 1.82], P_*adj*_ = 0.004), while acetate levels remained significantly lower (-0.92, [-1.40, -0.43], P_*adj*_ = 0.023), and DHA, while still elevated (0.87, [0.25, 1.48]), was no longer significant (P_*adj*_ = 0.141 (Fig. 1D **&** Supplementary Data D4). In JIA cases with inactive disease (*n* = 13), only DHA was still higher relative to controls amongst JIA-associated markers (1.35, [0.52, 2.17], P_*adj*_ = 0.041) (Fig. 1E **&** Supplementary Data D5). In addition, alanine, HDL particle size (HDL-size), phenylalanine, and HDL free cholesterol (HDL-FC) levels were also higher in inactive JIA, while acetoacetate and acetone were lower (Fig. 1E).

### Subtype-specific analysis identifies widespread metabolomic variation in systemic JIA

**Fig. 2:**
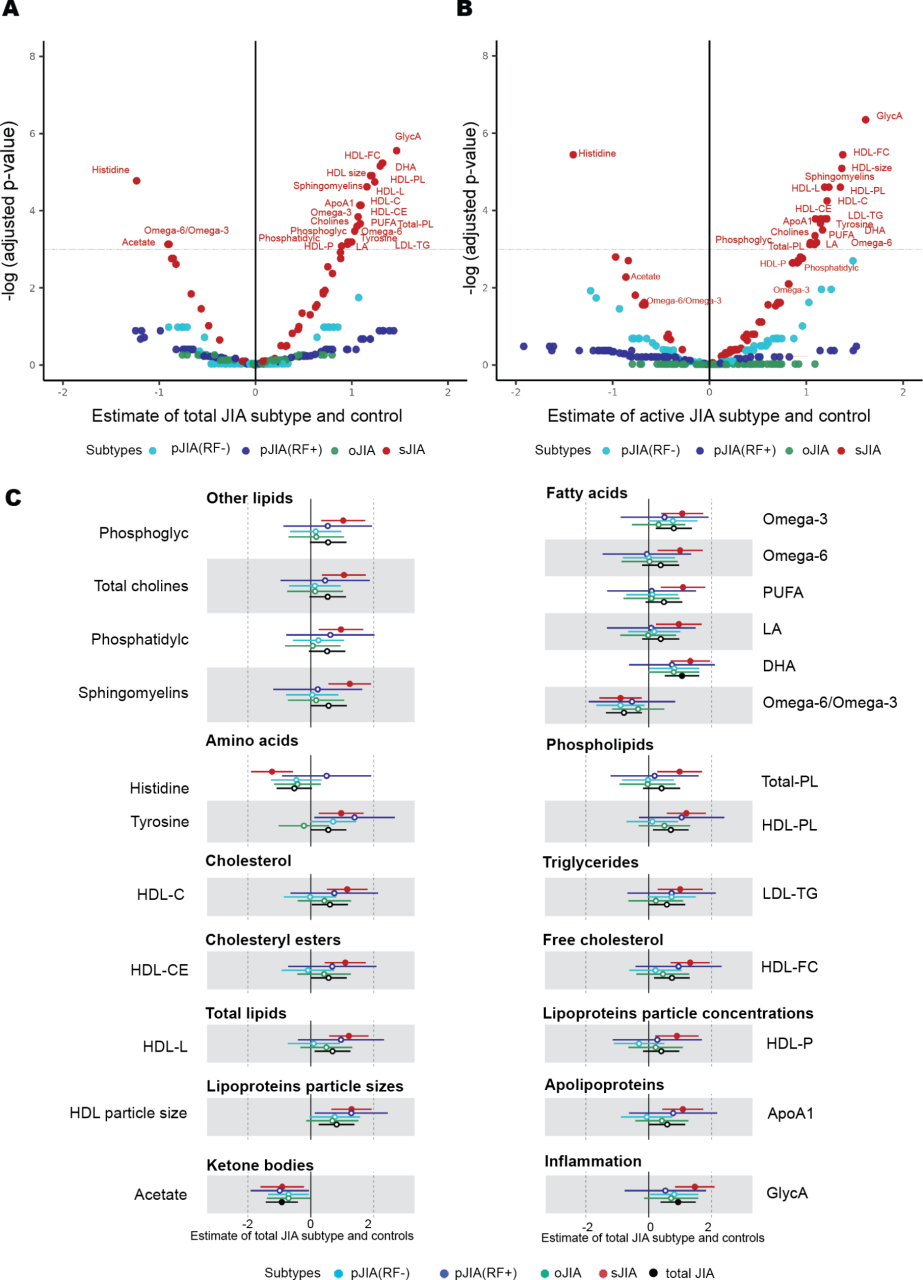
Subtype-associated analysis between four JIA subtypes (colored by subtype) **A.** Volcano plot for the estimated metabolomic difference between each subtype and control from adjusted linear regressions model (sJIA: *n* = 22, oJIA: *n* = 18, pJIA(RF-): *n* = 18 and pJIA(RF+): *n* = 14) and non-JIA controls (*n* = 18). (**A**) dotted line indicates *p*_*adj*_ value cut-off of 0.05. Biomarkers with *p*_*adj*_<0.05 in these subtype analyses are labelled. **(B)** Volcano plot for the estimated metabolomic difference between each subtype and control from adjusted linear regressions model in active subgroup (sJIA: *n* = 11, oJIA: *n* = 10, pJIA(RF-): *n* = 13 and pJIA(RF+): *n* = 9) and non-JIA controls (*n* = 18). A dotted line indicates *p*_*adj*_ value cut-off of 0.05. sJIA-associated biomarkers (*n* = 24) are labelled. **(C)** Forest plot for the estimated metabolomic difference between each subtype and control in 24 sJIA-associated biomarkers (circle points) from adjusted linear regression models (*n* = 90). Error bars are 95% confidence intervals. Closed points represent *p*_*adj*_<0.05 (BH). All models were adjusted for participants age and sex. Metabolomic difference between each subtype and control for all primary metabolomic measures are shown in Supplementary Figure S3

Linear regression was also used to identify metabolome differences associated with each of four JIA subtypes [sJIA, oJIA, pJIA(RF+) and pJIA(RF-)] relative to controls. No significant markers were identified in oJIA, pJIA(RF+) or pJIA(RF-) following adjustment for multiple testing (Fig. 2A **&** Supplementary Data D6). In contrast, 24 of 71 total measures were different in the sJIA subtype compared to controls (*P*_*ad*j_ <0.05; Fig. 2A, Supplementary Figure S3). This included amino acids (lower histidine, higher tyrosine), lower acetate, several fatty acid measures including higher omega-3, omega-6, linoleic acid (LA) and DHA, higher inflammatory GlycA and several lipid and cholesterol measures. These were higher levels of apolipoprotein A1 (ApoA1), sphingomyelins and various high-density lipoprotein (HDL) measures, including HDL-FC, HDL-size, HDL-associated lipids (HDL-L), HDL cholesterol (HDL-C), HDL cholesterol esters (HDL-CE), HDL particles (HDL-P) and HDL phospholipids (HDL-PL).

In models including only participants with active disease, GlycA and DHA remained significantly associated with active sJIA, while evidence for differences in several lipid markers, including HDL-P, Phosphatidylcholine (Phosphatidylc), omega-3 FA, and acetate was reduced (Fig. 2B **&** Supplementary Data D7). No evidence of metabolome changes in inactive sJIA patients was found (Supplementary Data D8). Whilst evidence for metabolomic differences was much weaker in non-sJIA subtypes, the estimated differences for a small number of measures showed a similar direction and/or magnitude of effect across all four subtypes, particularly acetate. (Fig. 2C).

### GlycA is more strongly associated with sJIA disease activity than hsCRP

**Fig. 3:**
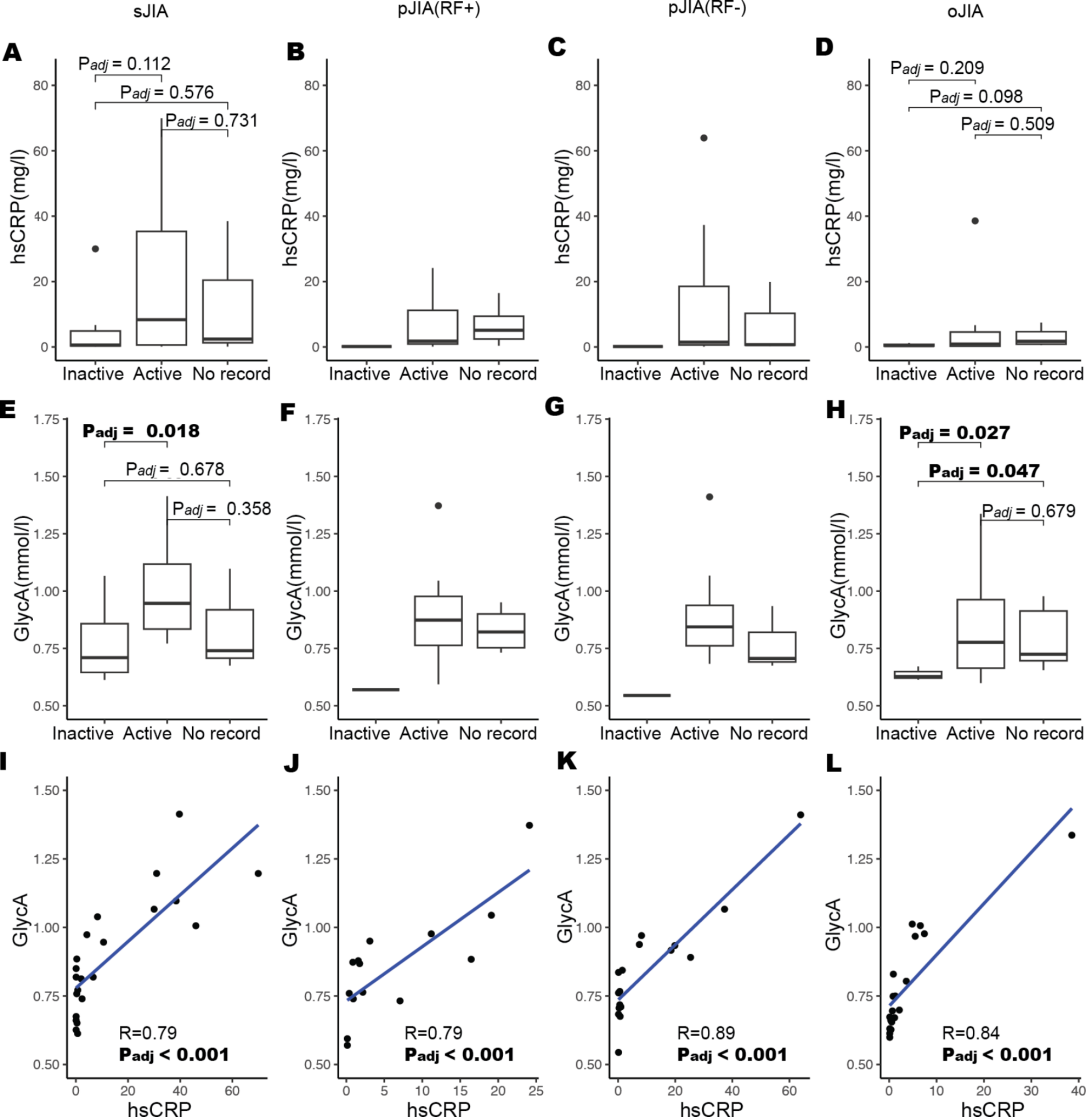
Boxplots of hsCRP (**A-D**) and GlycA (**E-H**) by disease activity groups (inactive, active, no record) across different JIA subtypes. P_*adj*_ value is corrected by BH and the significance between groups are marked by *p*-values. The scatter plots (I-L) show Pearson’s correlation between hsCRP and GlycA levels with a linear fitted line, demonstrating a strong positive correlation across all subtypes. R-value and P_*adj*_ value are marked. (A, E, I: sJIA, B,F, J: pJIA(RF+), C, G,K: pJIA(RF-), D, H,L: oJIA.)

Across all subtypes, hsCRP levels tended to be higher in the active group compared to the inactive group (Fig. 3A-D). However, these differences were not statistically significant in sJIA (Fig. 3A). Additionally, only one patient for each of pJIA RF- and RF + had inactive status, preventing statistical comparison (Fig. 3B **and C**). Similarly, the trends of GlycA levels were higher in active patients than inactive (Fig. 3E and H). Notably, GlycA levels were significantly higher in the active sJIA disease group compared to inactive group (0.25 mmol/L higher, [-0.40, -0.04], P_*adj*_ =0.018) (Fig. 3E), and were higher in active oJIA compared to inactive (0.50 mmol/L higher, [0.03, 0.36], P_*adj*_=0.027) (Fig. 3H). There was a strong positive correlation between hsCRP and GlycA across all JIA subtypes (sJIA: Pearson’s correlation (R) = 0.79 [Fig. 3I], pJIA(RF-): *R* = 0.79 [Fig. 3J], pJIA(RF+): *R* = 0.89 [Fig. 3K], and oJIA: *R* = 0.84 [Fig. 3L]).

### Marker of chronic (GlycA), rather than acute (hsCRP), inflammation captures more variation in the metabolome of sJIA

**Fig. 4:**
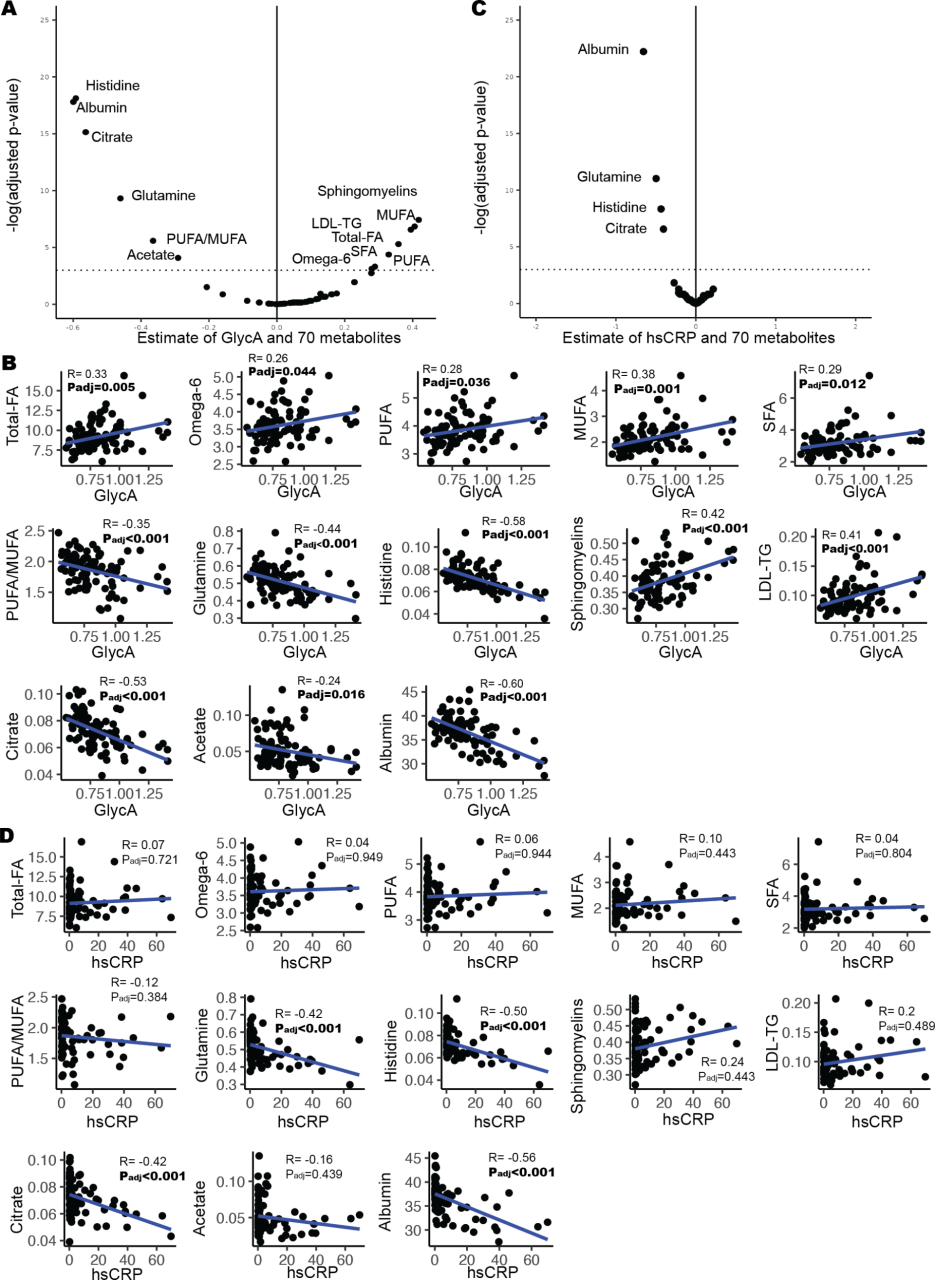
Biomarkers that are associated with chronic inflammation (GlycA) **(A)** Volcano plot for the estimated association between level of 70 primary metabolomic measures and GlycA (*n* = 90) from adjusted linear regressions model. A dotted line indicates *p*_*adj*_ value cut-off of 0.05. Biomarkers with *p*_*adj*_<0.05 are labelled. **(B)** A scatter plot for 13 GlycA-associated biomarkers (*n* = 90). The distribution in the whole cohort were described by scatter plot (black dots) and the associations between GlycA and potential biomarkers were illustrated with a linear fitted line (blue). **(C)** Volcano plot for the estimated association between level of 70 primary metabolomic measures and hsCRP (*n* = 90) from adjusted linear regressions model. A dotted line indicates *p*_*adj*_ value cut-off of 0.05. Biomarkers with *p*_*adj*_<0.05 are labelled. **(D)** A scatter plot for 4 hsCRP-associated biomarkers (*n* = 90). The distribution in the whole cohort were described by scatter plot (black dots) and the associations between hsCRP and potential biomarkers were illustrated with a linear fitted line (blue)

Next, we wanted to investigate the extent to which differences observed in the metabolome between sJIA and control groups are associated with underlying inflammation. We assessed the relationship between GlycA with each of the other 70 metabolomic measures in all participants (*n* = 90). GlycA was associated with 13 biomarkers (*P*_*adj*_<0.05) (Fig. 4A, Supplementary Data D9). Of these, 6 biomarkers (polyunsaturated fatty acids (PUFA), sphingomyelins, low density lipoprotein triglycerides (LDL-TG), acetate, histidine, and omega-6 fatty acids) also overlapped with the 24 sJIA-associated biomarkers identified in subtype analysis (Fig. 2A). Correlation plots of GlycA and each of the 13 GlycA-associated biomarkers are shown in Fig. 4B. We also assessed the relationship between hsCRP and the same 70 biomarkers (Fig. 4C **&** Supplementary Data D10). Correlation plots of hsCRP and each of the 13 GlycA-associated biomarkers are shown in Fig. 4D. hsCRP was associated with 4 of the same biomarkers as GlycA, generally with a similar strength of correlation: albumin (GlycA *R*=-0.60; hsCRP *R*=-0.56), glutamine (GlycA *R*=-0.44; hsCRP *R*=-0.42), histidine (GlycA *R*=-0.58; hsCRP *R*=-0.50), and citrate (GlycA *R*=-0.53; hsCRP *R*=-0.42 (Fig. 4B and D**)**.

## Discussion

In this study, we used targeted metabolomics to identify metabolic differences between JIA patients and non-JIA controls in plasma. The main benefits of the NMR-based targeted platform used here include clinically validated biomarkers, absolute quantification of metabolites, and high reproducibility owing to no batch effects [33]. Another strength of our approach was the analysis of four JIA subtypes. A previous untargeted liquid chromatography/mass spectrometry metabolomics study reported some differences in metabolites between JIA (*n* = 20) and controls (*n* = 20) but did not investigate JIA subtype-specific differences [12].

Of the 71 metabolic biomarkers investigated, three showed clear differences between children with JIA relative to controls, some of which differed by disease status at the time of blood collection. GlycA—a marker of cumulative inflammation—showed the largest difference in the active JIA group compared to controls. Previous studies have similarly reported that GlycA levels are elevated in individuals with SLE and RA and are associated with the severity of these diseases [34, 35]. We found elevated DHA levels in plasma in sJIA, in contrast to a previous JIA study that reported lower levels of polyunsaturated fatty acids in relation to active disease [36]. However, the difference in DHA between JIA cases and controls appeared specific to JIA cases with inactive disease, potentially reflecting medication use [37, 38]. Participants in our study received medications such as corticosteroids, NSAIDs and disease modifying anti rheumatoid drugs (DMARDs). In this cross-sectional study, the impact of medications on biomarkers is difficult to determine due to the non-random assignment of medication use, affected by factors including disease status, timing of diagnosis, and medication intolerance. Our findings suggest that active and inactive disease status in JIA is associated with distinct metabolic profile, with a chronic inflammatory marker, GlycA, prominent in active disease and lipid/amino acid metabolism differences becoming more prominent in remission.

In subtype-specific analysis, metabolic variation was most evident for sJIA, which has the most severe inflammation and is classified as an autoinflammatory disease (driven by innate immunity), as opposed to other subtypes that are primarily autoimmune (driven by adaptive immunity) in nature [1, 4, 39, 40]. ​While HDL has anti-inflammatory properties [41], our results showed that HDL-associated markers and ApoA1 were significantly increased in total sJIA and remained significant in active sJIA group. This contrasts with a meta-analysis that found no significant differences in HDL levels between JIA patients and healthy controls, though the included studies had a high degree of heterogeneity (I^2^ = 98.0%), indicating substantial variability between studies [42]. Furthermore, increased HDL levels in sJIA patients identified in our study might reflect specific subgroups not captured in the meta-analysis, such as patients with certain disease characteristics or those undergoing particular treatments. Unlike other childhood inflammatory conditions such as jSLE [43], we did not see strong evidence for LDL measures except for LDL-TG differing between sJIA and controls.

The absence of clear metabolic differences in plasma of children with oJIA and pJIA subtypes may reflect that sJIA is characterised by systemic inflammation affecting the circulation, in contrast to oJIA and pJIA, which show localized inflammation at the joints [44, 45]. It is possible that the metabolome of non-systemic JIA might show greater variation compared to controls within the affected joints than in plasma, however this requires investigation.

We found that hsCRP and GlycA were highly correlated across all JIA subtypes. However, only GlycA was significantly elevated in the active disease group compared to the inactive group in sJIA and oJIA subtypes. Further, we found that the level of chronic inflammation (as measured by GlycA) may explain some of the metabolic differences observed in sJIA relative to other JIA subtypes. GlycA was positively associated with 4 sJIA-associated biomarkers (LDL-TG, PUFA, omega-6 and sphingomyelins) and negatively associated with 2 (acetate and histidine). This negative association is consistent with previous evidence that acetate and histidine have anti-inflammatory properties and regulate the production of pro-inflammatory cytokines and the activation of inflammatory cells [46]. While histidine-rich glycoprotein (HRG) has been identified as a biomarker in adult RA [47], acetate has not previously been linked to arthritis. The weaker association of hsCRP and sJIA- associated metabolomic changes in JIA suggests the chronic aspects of inflammation may be more relevant to metabolic alterations in sJIA than acute inflammation.

Overall, our findings indicate that only some of the JIA-associated metabolic biomarkers were also inflammation-associated, suggesting that there are metabolic signatures in sJIA that are independent of inflammation. Further, as GlycA is a composite marker of several inflammatory processes in the body, such as elevated inflammatory cytokines and neutrophils, it is difficult to determine which of these cellular processes are contributing most to metabolomic differences [24, 48]. Future research is needed to characterize other soluble inflammatory mediators such as cytokines and inflammatory cells in JIA, and to explore opportunities to use metabolic and inflammatory biomarkers to better distinguish JIA subtypes, particularly sJIA, from other inflammatory conditions such as jSLE [49].

### Strengths and limitations

This study investigated the association between the metabolome and JIA, as well as chronic inflammation, using a targeted NMR metabolomic approach. Our pediatric JIA cohort is large compared to previous metabolomic studies in JIA and included four distinct subtypes. Certain limitations merit consideration. NMR-based metabolomics is generally less sensitive and less comprehensive than mass-spectrometry based platforms, raising the possibility that untargeted metabolomics profiling may identify circulating biomarkers not measured here [50]. Our control cohort was younger on average than our JIA cohort, which may influence the results. However, age was included as a covariate in all statistical models. The limited number of rarer subtypes, such as only 14 cases of pJIA (RF+), affected the statistical power for relevant subtype analyses. Of note, the pJIA (RF+) group in this cohort were all female and older on average than the other subtypes, in line with the known higher prevalence of girls (8–9 females to 1 male) and age range (generally 10 to 13 years of age) [51]. Our results showed no significant changes in the plasma metabolome of pJIA(RF+) patients compared to controls. Hormonal and metabolic changes typically associated with puberty in this age range [52] may be involved, however this was not formally tested.

The lack of data for the medication history, including the duration of medication and symptom improvement after the treatment, means we were unable to account for these potential confounding factors. Additionally, the broad “active disease” category used in this study included patients with varying levels of disease severity. This wide range of clinical features, particularly for non-sJIA patients, may have diminished our ability to identify effects of disease activity on metabolomic associations. Future studies should investigate more detailed categories of disease activity to better understand the relationship between these biomarkers and disease severity.

## Conclusion

The plasma NMR metabolome of systemic JIA is different to non-JIA controls, where as other JIA subtypes show limited evidence of metabolomic disruption compared to controls. Some of the sJIA-associated metabolomic variation tracks with underlying inflammatory status. These findings suggest a potential clinical application for classifying and monitoring JIA with targeted metabolomics with inflammation. Furthermore, these data support GlycA as a novel marker for monitoring disease activity in sJIA.

## Electronic supplementary material

Below is the link to the electronic supplementary material.

**Supplementary Material 1**: **Supplementary Figure S1**. Heatmap for the correlation between 249 NMR biomarkers. The heatmap represents a correlation matrix computed using Spearman’s rank correlation coefficient, denoted as r, ranging from − 1 (perfect negative correlation) to 1 (perfect positive correlation), with 0 indicating no correlation. Each cell in the heatmap corresponds to the correlation between two biomarkers. The colour scale from blue to red represents the strength and direction of the correlation (Dark blue: -1 < *r*<-0.8, light blue: -0.79 < *r*<-0.5, white: -0.09 < *r* < 0, yellow: 0.11 < *r* < 0.49, red: 0.5 < *r* < 1).

**Supplementary Material 2**: **Supplementary Figure S2**. The distribution difference between JIA group and controls for all primary metabolomic measures (*n* = 90). Student’s T test for JIA-associated markers between JIA group and controls. *p*_*adj*_ values were marked above dendrogram. All models were adjusted for participants age and sex.

**Supplementary Material 3**: **Supplementary Figure S3**. Forest plot for the estimated metabolomic difference between each subtype and control for biomarkers not associated with sJIA (46 biomarkers) from adjusted linear regression models (*n* = 90). Error bars are 95% confidence intervals. Closed points represent *p*_*adj*_ values < 0.05 (BH). All models were adjusted for participants age and sex.

**Supplementary Material 4: Supplementary Data D1-D10.**

## Data Availability

The datasets analysed during the current study are available from the corresponding author on reasonable request, following approval from CLARITY data custodians.

## Abbreviations

JIA: Juvenile idiopathic arthritis (JIA)
ILAR: International League of Associations for Rheumatology
RF: Rheumatoid factor
oJIA: Oligoarticular JIA
pJIA: Polyarticular JIA
pJIA(RF-): RF negative pJIA
pJIA(RF+): RF positive pJIA
RA: Rheumatoid arthritis
sJIA: Systemic JIA
DMARDs: Disease modifying anti-rheumatic drugs
IBD: Inflammatory Bowel Disease
SLE: Systemic Lupus Erythematosus
NMR: Nuclear magnetic resonance
GlycA: Glycoprotein acetyls
CRP: C-reactive protein
CLARITY: ChiLdhood Arthritis Risk factor Identification sTudY
HREC: Human Research Ethics Committee
RCH: Royal Children’s Hospital
AMD: Adjusted mean difference
CI: Confidence interval
FDR: False discovery rate
BH: Benjamini-Hochberg
*p*_*adj*_: Adjusted *P* value
HDL: High-density lipoprotein
Phe: Phenylalanine
PUFA: Polyunsaturated fatty acids
LDL-TG: Low density lipoprotein triglycerides
NSAIDs: Non-steroid anti-inflammatory drugs
DHA: Docosahexaenoic acid
