# Supplementary figures and images for: The plasma metabolome of juvenile idiopathic arthritis varies according to subtype and underlying inflammatory status
Source: Pediatr Rheumatol Online J. 2024 Dec 30;22:113. doi: 10.1186/s12969-024-01041-8 (PMC11686955; doi:10.1186/s12969-024-01041-8)

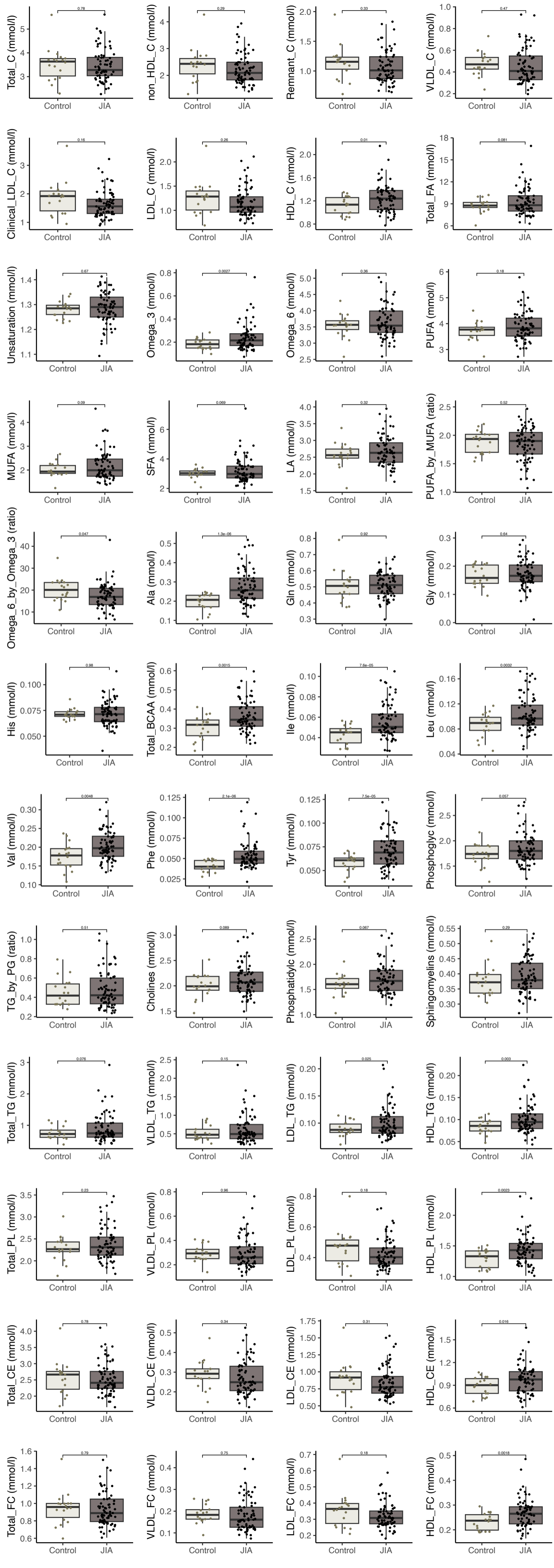

Supplement: Supplementary file 1 — Supplementary Material 1: Supplementary Figure S1. Heatmap for the correlation between 249 NMR biomarkers. The heatmap represents a correlation matrix computed using Spearman’s rank correlation coefficient, denoted as r, ranging from − 1 (perfect negative correlation) to 1 (perfect positive correlation), with 0 indicating no correlation. Each cell in the heatmap corresponds to the correlation between two biomarkers. The colour scale from blue to red represents the strength and direction of the correlation (Dark blue: -1 < r<-0.8, light blue: -0.79 < r<-0.5, white: -0.09 < r < 0, yellow: 0.11 < r < 0.49, red: 0.5 < r < 1). [file 12969_2024_1041_MOESM1_ESM.pdf]

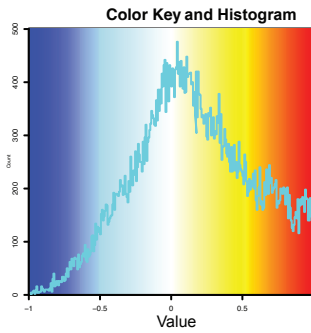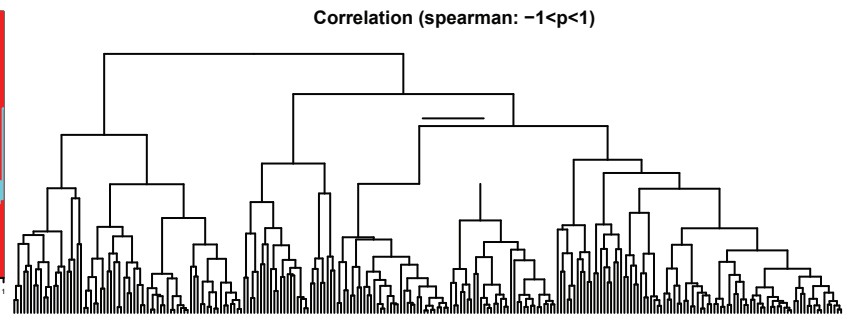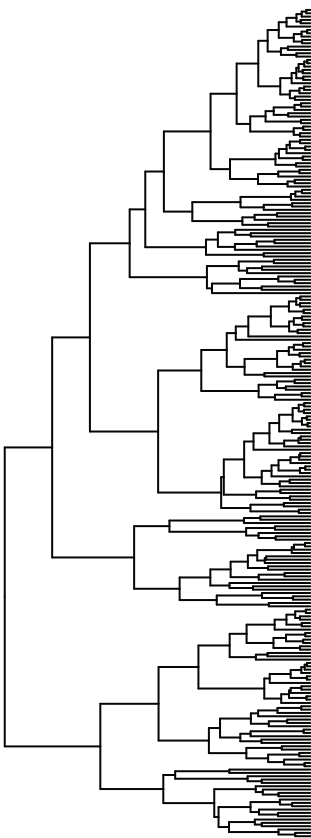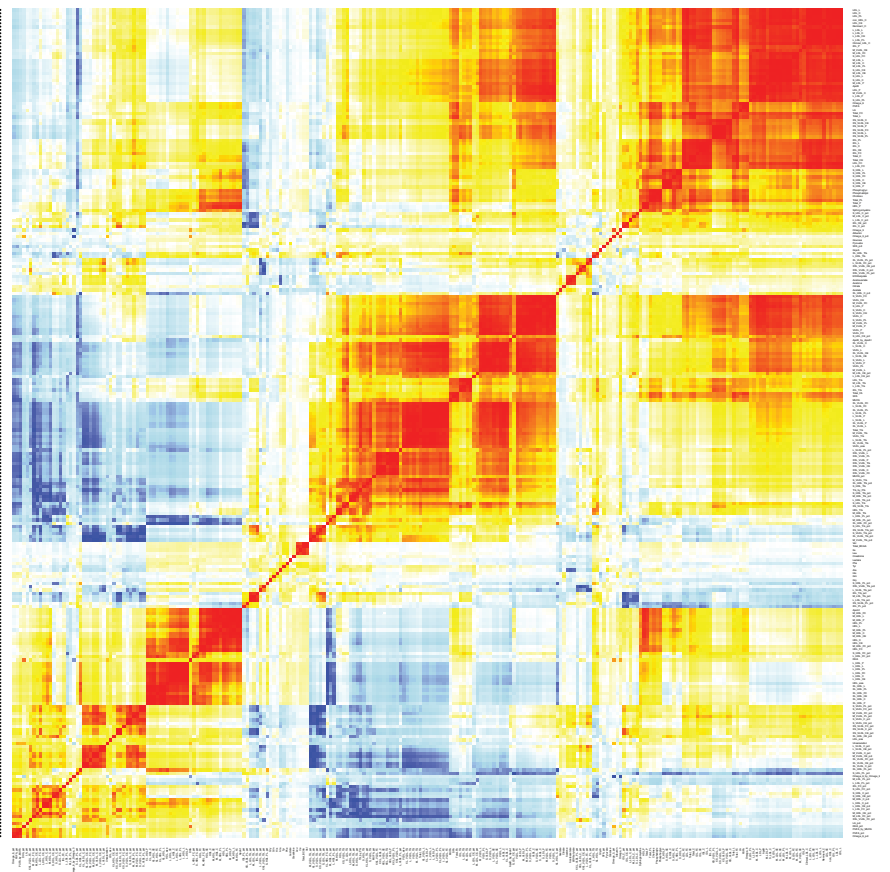

Supplement: Supplementary file 2 — Supplementary Material 2: Supplementary Figure S2. The distribution difference between JIA group and controls for all primary metabolomic measures (n = 90). Student’s T test for JIA-associated markers between JIA group and controls. padj values were marked above dendrogram. All models were adjusted for participants age and sex. [file 12969_2024_1041_MOESM2_ESM.pdf]

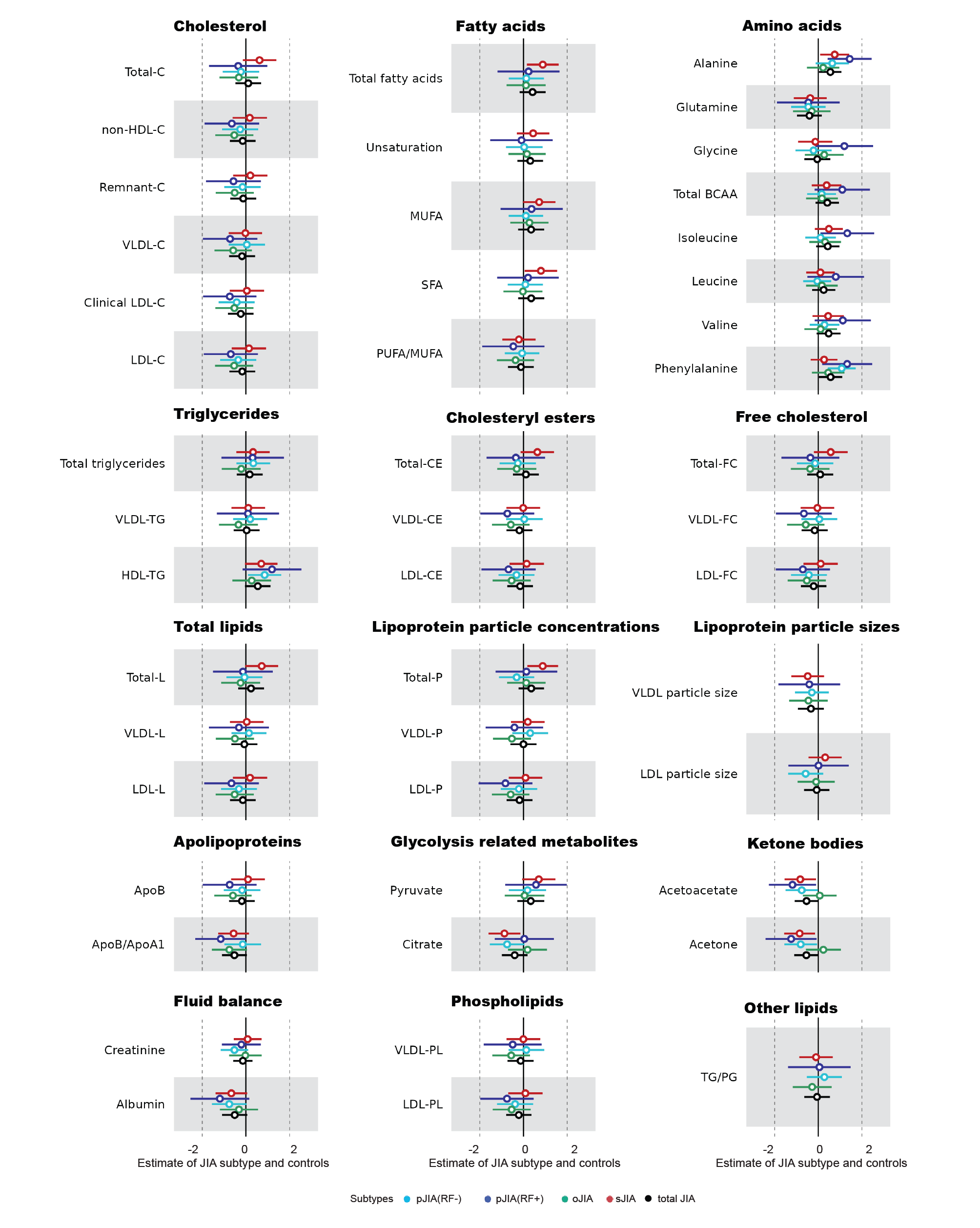

Supplement: Supplementary file 3 — Supplementary Material 3: Supplementary Figure S3. Forest plot for the estimated metabolomic difference between each subtype and control for biomarkers not associated with sJIA (46 biomarkers) from adjusted linear regression models (n = 90). Error bars are 95% confidence intervals. Closed points represent padj values < 0.05 (BH). All models were adjusted for participants age and sex. [file 12969_2024_1041_MOESM3_ESM.png]
