# Supplementary material for: The plasma metabolome of juvenile idiopathic arthritis varies according to subtype and underlying inflammatory status
Source: Pediatr Rheumatol Online J. 2024 Dec 30;22:113. doi: 10.1186/s12969-024-01041-8 (PMC11686955; doi:10.1186/s12969-024-01041-8)
Supplement: Supplementary file 4 — Supplementary Material 4: Supplementary Data D1-D10. [file 12969_2024_1041_MOESM4_ESM.docx]

**Supplementary Data D1.** List of initial 249 metabolites

| Biomarker | Abbreviation | Unit | Group |
| --- | --- | --- | --- |
| Total cholesterol | Total-C | mmol/l | Cholesterol |
| Total cholesterol minus HDL-C | non-HDL-C | mmol/l | Cholesterol |
| Remnant cholesterol (non-HDL, non-LDL -cholesterol) | Remnant-C | mmol/l | Cholesterol |
| VLDL cholesterol | VLDL-C | mmol/l | Cholesterol |
| Clinical LDL cholesterol | Clinical LDL-C | mmol/l | Cholesterol |
| LDL cholesterol | LDL-C | mmol/l | Cholesterol |
| HDL cholesterol | HDL-C | mmol/l | Cholesterol |
| Total triglycerides | Total-TG | mmol/l | Triglycerides |
| Triglycerides in VLDL | VLDL-TG | mmol/l | Triglycerides |
| Triglycerides in LDL | LDL-TG | mmol/l | Triglycerides |
| Triglycerides in HDL | HDL-TG | mmol/l | Triglycerides |
| Total phospholipids in lipoprotein particles | Total-PL | mmol/l | Phospholipids |
| Phospholipids in VLDL | VLDL-PL | mmol/l | Phospholipids |
| Phospholipids in LDL | LDL-PL | mmol/l | Phospholipids |
| Phospholipids in HDL | HDL-PL | mmol/l | Phospholipids |
| Total esterified cholesterol | Total-CE | mmol/l | Cholesteryl esters |
| Cholesteryl esters in VLDL | VLDL-CE | mmol/l | Cholesteryl esters |
| Cholesteryl esters in LDL | LDL-CE | mmol/l | Cholesteryl esters |
| Cholesteryl esters in HDL | HDL-CE | mmol/l | Cholesteryl esters |
| Total free cholesterol | Total-FC | mmol/l | Free cholesterol |
| Free cholesterol in VLDL | VLDL-FC | mmol/l | Free cholesterol |
| Free cholesterol in LDL | LDL-FC | mmol/l | Free cholesterol |
| Free cholesterol in HDL | HDL-FC | mmol/l | Free cholesterol |
| Total lipids in lipoprotein particles | Total-L | mmol/l | Total lipids |
| Total lipids in VLDL | VLDL-L | mmol/l | Total lipids |
| Total lipids in LDL | LDL-L | mmol/l | Total lipids |
| Total lipids in HDL | HDL-L | mmol/l | Total lipids |
| Total concentration of lipoprotein particles | Total-P | mmol/l | Lipoprotein particle concentrations |
| Concentration of VLDL particles | VLDL-P | mmol/l | Lipoprotein particle concentrations |
| Concentration of LDL particles | LDL-P | mmol/l | Lipoprotein particle concentrations |
| Concentration of HDL particles | HDL-P | mmol/l | Lipoprotein particle concentrations |
| Average diameter for VLDL particles | VLDL size | nm | Lipoprotein particle sizes |
| Average diameter for LDL particles | LDL size | nm | Lipoprotein particle sizes |
| Average diameter for HDL particles | HDL size | nm | Lipoprotein particle sizes |
| Phosphoglycerides | Phosphoglyc | mmol/l | Other lipids |
| Ratio of triglycerides to phosphoglycerides | TG/PG | ratio | Other lipids |
| Total cholines | Cholines | mmol/l | Other lipids |
| Phosphatidylcholines | Phosphatidylc | mmol/l | Other lipids |
| Sphingomyelins | Sphingomyelins | mmol/l | Other lipids |
| Apolipoprotein B | ApoB | g/l | Apolipoproteins |
| Apolipoprotein A1 | ApoA1 | g/l | Apolipoproteins |
| Ratio of apolipoprotein B to apolipoprotein A1 | ApoB/ApoA1 | ratio | Apolipoproteins |
| Total fatty acids | Total-FA | mmol/l | Fatty acids |
| Degree of unsaturation | Unsaturation | degree | Fatty acids |
| Omega-3 fatty acids | Omega-3 | mmol/l | Fatty acids |
| Omega-6 fatty acids | Omega-6 | mmol/l | Fatty acids |
| Polyunsaturated fatty acids | PUFA | mmol/l | Fatty acids |
| Monounsaturated fatty acids | MUFA | mmol/l | Fatty acids |
| Saturated fatty acids | SFA | mmol/l | Fatty acids |
| Linoleic acid | LA | mmol/l | Fatty acids |
| Docosahexaenoic acid | DHA | mmol/l | Fatty acids |
| Ratio of omega-3 fatty acids to total fatty acids | Omega-3 % | % | Fatty acids |
| Ratio of omega-6 fatty acids to total fatty acids | Omega-6 % | % | Fatty acids |
| Ratio of polyunsaturated fatty acids to total fatty acids | PUFA % | % | Fatty acids |
| Ratio of monounsaturated fatty acids to total fatty acids | MUFA % | % | Fatty acids |
| Ratio of saturated fatty acids to total fatty acids | SFA % | % | Fatty acids |
| Ratio of linoleic acid to total fatty acids | LA % | % | Fatty acids |
| Ratio of docosahexaenoic acid to total fatty acids | DHA % | % | Fatty acids |
| Ratio of polyunsaturated fatty acids to monounsaturated fatty acids | PUFA/MUFA | ratio | Fatty acids |
| Ratio of omega-6 fatty acids to omega-3 fatty acids | Omega-6/Omega-3 | ratio | Fatty acids |
| Alanine | Ala | mmol/l | Amino acids |
| Glutamine | Gln | mmol/l | Amino acids |
| Glycine | Gly | mmol/l | Amino acids |
| Histidine | His | mmol/l | Amino acids |
| Total concentration of branched-chain amino acids (leucine + isoleucine + valine) | Total BCAA | mmol/l | Amino acids |
| Isoleucine | Ile | mmol/l | Amino acids |
| Leucine | Leu | mmol/l | Amino acids |
| Valine | Val | mmol/l | Amino acids |
| Phenylalanine | Phe | mmol/l | Amino acids |
| Tyrosine | Tyr | mmol/l | Amino acids |
| Glucose | Glucose | mmol/l | Glycolysis related metabolites |
| Lactate | Lactate | mmol/l | Glycolysis related metabolites |
| Pyruvate | Pyruvate | mmol/l | Glycolysis related metabolites |
| Citrate | Citrate | mmol/l | Glycolysis related metabolites |
| 3-Hydroxybutyrate | bOHbutyrate | mmol/l | Ketone bodies |
| Acetate | Acetate | mmol/l | Ketone bodies |
| Acetoacetate | Acetoacetate | mmol/l | Ketone bodies |
| Acetone | Acetone | mmol/l | Ketone bodies |
| Creatinine | Creatinine | _mol/l | Fluid balance |
| Albumin | Albumin | g/l | Fluid balance |
| Glycoprotein acetyls | GlycA | mmol/l | Inflammation |
| Concentration of chylomicrons and extremely large VLDL particles | XXL-VLDL-P | mmol/l | Lipoprotein subclasses |
| Total lipids in chylomicrons and extremely large VLDL | XXL-VLDL-L | mmol/l | Lipoprotein subclasses |
| Phospholipids in chylomicrons and extremely large VLDL | XXL-VLDL-PL | mmol/l | Lipoprotein subclasses |
| Cholesterol in chylomicrons and extremely large VLDL | XXL-VLDL-C | mmol/l | Lipoprotein subclasses |
| Cholesteryl esters in chylomicrons and extremely large VLDL | XXL-VLDL-CE | mmol/l | Lipoprotein subclasses |
| Free cholesterol in chylomicrons and extremely large VLDL | XXL-VLDL-FC | mmol/l | Lipoprotein subclasses |
| Triglycerides in chylomicrons and extremely large VLDL | XXL-VLDL-TG | mmol/l | Lipoprotein subclasses |
| Concentration of very large VLDL particles | XL-VLDL-P | mmol/l | Lipoprotein subclasses |
| Total lipids in very large VLDL | XL-VLDL-L | mmol/l | Lipoprotein subclasses |
| Phospholipids in very large VLDL | XL-VLDL-PL | mmol/l | Lipoprotein subclasses |
| Cholesterol in very large VLDL | XL-VLDL-C | mmol/l | Lipoprotein subclasses |
| Cholesteryl esters in very large VLDL | XL-VLDL-CE | mmol/l | Lipoprotein subclasses |
| Free cholesterol in very large VLDL | XL-VLDL-FC | mmol/l | Lipoprotein subclasses |
| Triglycerides in very large VLDL | XL-VLDL-TG | mmol/l | Lipoprotein subclasses |
| Concentration of large VLDL particles | L-VLDL-P | mmol/l | Lipoprotein subclasses |
| Total lipids in large VLDL | L-VLDL-L | mmol/l | Lipoprotein subclasses |
| Phospholipids in large VLDL | L-VLDL-PL | mmol/l | Lipoprotein subclasses |
| Cholesterol in large VLDL | L-VLDL-C | mmol/l | Lipoprotein subclasses |
| Cholesteryl esters in large VLDL | L-VLDL-CE | mmol/l | Lipoprotein subclasses |
| Free cholesterol in large VLDL | L-VLDL-FC | mmol/l | Lipoprotein subclasses |
| Triglycerides in large VLDL | L-VLDL-TG | mmol/l | Lipoprotein subclasses |
| Concentration of medium VLDL particles | M-VLDL-P | mmol/l | Lipoprotein subclasses |
| Total lipids in medium VLDL | M-VLDL-L | mmol/l | Lipoprotein subclasses |
| Phospholipids in medium VLDL | M-VLDL-PL | mmol/l | Lipoprotein subclasses |
| Cholesterol in medium VLDL | M-VLDL-C | mmol/l | Lipoprotein subclasses |
| Cholesteryl esters in medium VLDL | M-VLDL-CE | mmol/l | Lipoprotein subclasses |
| Free cholesterol in medium VLDL | M-VLDL-FC | mmol/l | Lipoprotein subclasses |
| Triglycerides in medium VLDL | M-VLDL-TG | mmol/l | Lipoprotein subclasses |
| Concentration of small VLDL particles | S-VLDL-P | mmol/l | Lipoprotein subclasses |
| Total lipids in small VLDL | S-VLDL-L | mmol/l | Lipoprotein subclasses |
| Phospholipids in small VLDL | S-VLDL-PL | mmol/l | Lipoprotein subclasses |
| Cholesterol in small VLDL | S-VLDL-C | mmol/l | Lipoprotein subclasses |
| Cholesteryl esters in small VLDL | S-VLDL-CE | mmol/l | Lipoprotein subclasses |
| Free cholesterol in small VLDL | S-VLDL-FC | mmol/l | Lipoprotein subclasses |
| Triglycerides in small VLDL | S-VLDL-TG | mmol/l | Lipoprotein subclasses |
| Concentration of very small VLDL particles | XS-VLDL-P | mmol/l | Lipoprotein subclasses |
| Total lipids in very small VLDL | XS-VLDL-L | mmol/l | Lipoprotein subclasses |
| Phospholipids in very small VLDL | XS-VLDL-PL | mmol/l | Lipoprotein subclasses |
| Cholesterol in very small VLDL | XS-VLDL-C | mmol/l | Lipoprotein subclasses |
| Cholesteryl esters in very small VLDL | XS-VLDL-CE | mmol/l | Lipoprotein subclasses |
| Free cholesterol in very small VLDL | XS-VLDL-FC | mmol/l | Lipoprotein subclasses |
| Triglycerides in very small VLDL | XS-VLDL-TG | mmol/l | Lipoprotein subclasses |
| Concentration of IDL particles | IDL-P | mmol/l | Lipoprotein subclasses |
| Total lipids in IDL | IDL-L | mmol/l | Lipoprotein subclasses |
| Phospholipids in IDL | IDL-PL | mmol/l | Lipoprotein subclasses |
| Cholesterol in IDL | IDL-C | mmol/l | Lipoprotein subclasses |
| Cholesteryl esters in IDL | IDL-CE | mmol/l | Lipoprotein subclasses |
| Free cholesterol in IDL | IDL-FC | mmol/l | Lipoprotein subclasses |
| Triglycerides in IDL | IDL-TG | mmol/l | Lipoprotein subclasses |
| Concentration of large LDL particles | L-LDL-P | mmol/l | Lipoprotein subclasses |
| Total lipids in large LDL | L-LDL-L | mmol/l | Lipoprotein subclasses |
| Phospholipids in large LDL | L-LDL-PL | mmol/l | Lipoprotein subclasses |
| Cholesterol in large LDL | L-LDL-C | mmol/l | Lipoprotein subclasses |
| Cholesteryl esters in large LDL | L-LDL-CE | mmol/l | Lipoprotein subclasses |
| Free cholesterol in large LDL | L-LDL-FC | mmol/l | Lipoprotein subclasses |
| Triglycerides in large LDL | L-LDL-TG | mmol/l | Lipoprotein subclasses |
| Concentration of medium LDL particles | M-LDL-P | mmol/l | Lipoprotein subclasses |
| Total lipids in medium LDL | M-LDL-L | mmol/l | Lipoprotein subclasses |
| Phospholipids in medium LDL | M-LDL-PL | mmol/l | Lipoprotein subclasses |
| Cholesterol in medium LDL | M-LDL-C | mmol/l | Lipoprotein subclasses |
| Cholesteryl esters in medium LDL | M-LDL-CE | mmol/l | Lipoprotein subclasses |
| Free cholesterol in medium LDL | M-LDL-FC | mmol/l | Lipoprotein subclasses |
| Triglycerides in medium LDL | M-LDL-TG | mmol/l | Lipoprotein subclasses |
| Concentration of small LDL particles | S-LDL-P | mmol/l | Lipoprotein subclasses |
| Total lipids in small LDL | S-LDL-L | mmol/l | Lipoprotein subclasses |
| Phospholipids in small LDL | S-LDL-PL | mmol/l | Lipoprotein subclasses |
| Cholesterol in small LDL | S-LDL-C | mmol/l | Lipoprotein subclasses |
| Cholesteryl esters in small LDL | S-LDL-CE | mmol/l | Lipoprotein subclasses |
| Free cholesterol in small LDL | S-LDL-FC | mmol/l | Lipoprotein subclasses |
| Triglycerides in small LDL | S-LDL-TG | mmol/l | Lipoprotein subclasses |
| Concentration of very large HDL particles | XL-HDL-P | mmol/l | Lipoprotein subclasses |
| Total lipids in very large HDL | XL-HDL-L | mmol/l | Lipoprotein subclasses |
| Phospholipids in very large HDL | XL-HDL-PL | mmol/l | Lipoprotein subclasses |
| Cholesterol in very large HDL | XL-HDL-C | mmol/l | Lipoprotein subclasses |
| Cholesteryl esters in very large HDL | XL-HDL-CE | mmol/l | Lipoprotein subclasses |
| Free cholesterol in very large HDL | XL-HDL-FC | mmol/l | Lipoprotein subclasses |
| Triglycerides in very large HDL | XL-HDL-TG | mmol/l | Lipoprotein subclasses |
| Concentration of large HDL particles | L-HDL-P | mmol/l | Lipoprotein subclasses |
| Total lipids in large HDL | L-HDL-L | mmol/l | Lipoprotein subclasses |
| Phospholipids in large HDL | L-HDL-PL | mmol/l | Lipoprotein subclasses |
| Cholesterol in large HDL | L-HDL-C | mmol/l | Lipoprotein subclasses |
| Cholesteryl esters in large HDL | L-HDL-CE | mmol/l | Lipoprotein subclasses |
| Free cholesterol in large HDL | L-HDL-FC | mmol/l | Lipoprotein subclasses |
| Triglycerides in large HDL | L-HDL-TG | mmol/l | Lipoprotein subclasses |
| Concentration of medium HDL particles | M-HDL-P | mmol/l | Lipoprotein subclasses |
| Total lipids in medium HDL | M-HDL-L | mmol/l | Lipoprotein subclasses |
| Phospholipids in medium HDL | M-HDL-PL | mmol/l | Lipoprotein subclasses |
| Cholesterol in medium HDL | M-HDL-C | mmol/l | Lipoprotein subclasses |
| Cholesteryl esters in medium HDL | M-HDL-CE | mmol/l | Lipoprotein subclasses |
| Free cholesterol in medium HDL | M-HDL-FC | mmol/l | Lipoprotein subclasses |
| Triglycerides in medium HDL | M-HDL-TG | mmol/l | Lipoprotein subclasses |
| Concentration of small HDL particles | S-HDL-P | mmol/l | Lipoprotein subclasses |
| Total lipids in small HDL | S-HDL-L | mmol/l | Lipoprotein subclasses |
| Phospholipids in small HDL | S-HDL-PL | mmol/l | Lipoprotein subclasses |
| Cholesterol in small HDL | S-HDL-C | mmol/l | Lipoprotein subclasses |
| Cholesteryl esters in small HDL | S-HDL-CE | mmol/l | Lipoprotein subclasses |
| Free cholesterol in small HDL | S-HDL-FC | mmol/l | Lipoprotein subclasses |
| Triglycerides in small HDL | S-HDL-TG | mmol/l | Lipoprotein subclasses |
| Phospholipids to total lipids ratio in chylomicrons and extremely large VLDL | XXL-VLDL-PL % | % | Relative lipoprotein lipid concentrations |
| Cholesterol to total lipids ratio in chylomicrons and extremely large VLDL | XXL-VLDL-C % | % | Relative lipoprotein lipid concentrations |
| Cholesteryl esters to total lipids ratio in chylomicrons and extremely large VLDL | XXL-VLDL-CE % | % | Relative lipoprotein lipid concentrations |
| Free cholesterol to total lipids ratio in chylomicrons and extremely large VLDL | XXL-VLDL-FC % | % | Relative lipoprotein lipid concentrations |
| Triglycerides to total lipids ratio in chylomicrons and extremely large VLDL | XXL-VLDL-TG % | % | Relative lipoprotein lipid concentrations |
| Phospholipids to total lipids ratio in very large VLDL | XL-VLDL-PL % | % | Relative lipoprotein lipid concentrations |
| Cholesterol to total lipids ratio in very large VLDL | XL-VLDL-C % | % | Relative lipoprotein lipid concentrations |
| Cholesteryl esters to total lipids ratio in very large VLDL | XL-VLDL-CE % | % | Relative lipoprotein lipid concentrations |
| Free cholesterol to total lipids ratio in very large VLDL | XL-VLDL-FC % | % | Relative lipoprotein lipid concentrations |
| Triglycerides to total lipids ratio in very large VLDL | XL-VLDL-TG % | % | Relative lipoprotein lipid concentrations |
| Phospholipids to total lipids ratio in large VLDL | L-VLDL-PL % | % | Relative lipoprotein lipid concentrations |
| Cholesterol to total lipids ratio in large VLDL | L-VLDL-C % | % | Relative lipoprotein lipid concentrations |
| Cholesteryl esters to total lipids ratio in large VLDL | L-VLDL-CE % | % | Relative lipoprotein lipid concentrations |
| Free cholesterol to total lipids ratio in large VLDL | L-VLDL-FC % | % | Relative lipoprotein lipid concentrations |
| Triglycerides to total lipids ratio in large VLDL | L-VLDL-TG % | % | Relative lipoprotein lipid concentrations |
| Phospholipids to total lipids ratio in medium VLDL | M-VLDL-PL % | % | Relative lipoprotein lipid concentrations |
| Cholesterol to total lipids ratio in medium VLDL | M-VLDL-C % | % | Relative lipoprotein lipid concentrations |
| Cholesteryl esters to total lipids ratio in medium VLDL | M-VLDL-CE % | % | Relative lipoprotein lipid concentrations |
| Free cholesterol to total lipids ratio in medium VLDL | M-VLDL-FC % | % | Relative lipoprotein lipid concentrations |
| Triglycerides to total lipids ratio in medium VLDL | M-VLDL-TG % | % | Relative lipoprotein lipid concentrations |
| Phospholipids to total lipids ratio in small VLDL | S-VLDL-PL % | % | Relative lipoprotein lipid concentrations |
| Cholesterol to total lipids ratio in small VLDL | S-VLDL-C % | % | Relative lipoprotein lipid concentrations |
| Cholesteryl esters to total lipids ratio in small VLDL | S-VLDL-CE % | % | Relative lipoprotein lipid concentrations |
| Free cholesterol to total lipids ratio in small VLDL | S-VLDL-FC % | % | Relative lipoprotein lipid concentrations |
| Triglycerides to total lipids ratio in small VLDL | S-VLDL-TG % | % | Relative lipoprotein lipid concentrations |
| Phospholipids to total lipids ratio in very small VLDL | XS-VLDL-PL % | % | Relative lipoprotein lipid concentrations |
| Cholesterol to total lipids ratio in very small VLDL | XS-VLDL-C % | % | Relative lipoprotein lipid concentrations |
| Cholesteryl esters to total lipids ratio in very small VLDL | XS-VLDL-CE % | % | Relative lipoprotein lipid concentrations |
| Free cholesterol to total lipids ratio in very small VLDL | XS-VLDL-FC % | % | Relative lipoprotein lipid concentrations |
| Triglycerides to total lipids ratio in very small VLDL | XS-VLDL-TG % | % | Relative lipoprotein lipid concentrations |
| Phospholipids to total lipids ratio in IDL | IDL-PL % | % | Relative lipoprotein lipid concentrations |
| Cholesterol to total lipids ratio in IDL | IDL-C % | % | Relative lipoprotein lipid concentrations |
| Cholesteryl esters to total lipids ratio in IDL | IDL-CE % | % | Relative lipoprotein lipid concentrations |
| Free cholesterol to total lipids ratio in IDL | IDL-FC % | % | Relative lipoprotein lipid concentrations |
| Triglycerides to total lipids ratio in IDL | IDL-TG % | % | Relative lipoprotein lipid concentrations |
| Phospholipids to total lipids ratio in large LDL | L-LDL-PL % | % | Relative lipoprotein lipid concentrations |
| Cholesterol to total lipids ratio in large LDL | L-LDL-C % | % | Relative lipoprotein lipid concentrations |
| Cholesteryl esters to total lipids ratio in large LDL | L-LDL-CE % | % | Relative lipoprotein lipid concentrations |
| Free cholesterol to total lipids ratio in large LDL | L-LDL-FC % | % | Relative lipoprotein lipid concentrations |
| Triglycerides to total lipids ratio in large LDL | L-LDL-TG % | % | Relative lipoprotein lipid concentrations |
| Phospholipids to total lipids ratio in medium LDL | M-LDL-PL % | % | Relative lipoprotein lipid concentrations |
| Cholesterol to total lipids ratio in medium LDL | M-LDL-C % | % | Relative lipoprotein lipid concentrations |
| Cholesteryl esters to total lipids ratio in medium LDL | M-LDL-CE % | % | Relative lipoprotein lipid concentrations |
| Free cholesterol to total lipids ratio in medium LDL | M-LDL-FC % | % | Relative lipoprotein lipid concentrations |
| Triglycerides to total lipids ratio in medium LDL | M-LDL-TG % | % | Relative lipoprotein lipid concentrations |
| Phospholipids to total lipids ratio in small LDL | S-LDL-PL % | % | Relative lipoprotein lipid concentrations |
| Cholesterol to total lipids ratio in small LDL | S-LDL-C % | % | Relative lipoprotein lipid concentrations |
| Cholesteryl esters to total lipids ratio in small LDL | S-LDL-CE % | % | Relative lipoprotein lipid concentrations |
| Free cholesterol to total lipids ratio in small LDL | S-LDL-FC % | % | Relative lipoprotein lipid concentrations |
| Triglycerides to total lipids ratio in small LDL | S-LDL-TG % | % | Relative lipoprotein lipid concentrations |
| Phospholipids to total lipids ratio in very large HDL | XL-HDL-PL % | % | Relative lipoprotein lipid concentrations |
| Cholesterol to total lipids ratio in very large HDL | XL-HDL-C % | % | Relative lipoprotein lipid concentrations |
| Cholesteryl esters to total lipids ratio in very large HDL | XL-HDL-CE % | % | Relative lipoprotein lipid concentrations |
| Free cholesterol to total lipids ratio in very large HDL | XL-HDL-FC % | % | Relative lipoprotein lipid concentrations |
| Triglycerides to total lipids ratio in very large HDL | XL-HDL-TG % | % | Relative lipoprotein lipid concentrations |
| Phospholipids to total lipids ratio in large HDL | L-HDL-PL % | % | Relative lipoprotein lipid concentrations |
| Cholesterol to total lipids ratio in large HDL | L-HDL-C % | % | Relative lipoprotein lipid concentrations |
| Cholesteryl esters to total lipids ratio in large HDL | L-HDL-CE % | % | Relative lipoprotein lipid concentrations |
| Free cholesterol to total lipids ratio in large HDL | L-HDL-FC % | % | Relative lipoprotein lipid concentrations |
| Triglycerides to total lipids ratio in large HDL | L-HDL-TG % | % | Relative lipoprotein lipid concentrations |
| Phospholipids to total lipids ratio in medium HDL | M-HDL-PL % | % | Relative lipoprotein lipid concentrations |
| Cholesterol to total lipids ratio in medium HDL | M-HDL-C % | % | Relative lipoprotein lipid concentrations |
| Cholesteryl esters to total lipids ratio in medium HDL | M-HDL-CE % | % | Relative lipoprotein lipid concentrations |
| Free cholesterol to total lipids ratio in medium HDL | M-HDL-FC % | % | Relative lipoprotein lipid concentrations |
| Triglycerides to total lipids ratio in medium HDL | M-HDL-TG % | % | Relative lipoprotein lipid concentrations |
| Phospholipids to total lipids ratio in small HDL | S-HDL-PL % | % | Relative lipoprotein lipid concentrations |
| Cholesterol to total lipids ratio in small HDL | S-HDL-C % | % | Relative lipoprotein lipid concentrations |
| Cholesteryl esters to total lipids ratio in small HDL | S-HDL-CE % | % | Relative lipoprotein lipid concentrations |
| Free cholesterol to total lipids ratio in small HDL | S-HDL-FC % | % | Relative lipoprotein lipid concentrations |
| Triglycerides to total lipids ratio in small HDL | S-HDL-TG % | % | Relative lipoprotein lipid concentrations |

**Supplementary Data D2.** Mean level of 71 metabolomic biomarkers by group

| Biomarker | Group | Mean |
| --- | --- | --- |
| Acetate | Ketone bodies | 5.06E-02 |
| Acetoacetate | Ketone bodies | 8.00E-02 |
| Acetone | Ketone bodies | 2.75E-02 |
| Alanine | Amino acids | 2.60E-01 |
| Albumin | Fluid balance | 3.66E+01 |
| ApoA1 | Apolipoproteins | 1.30E+00 |
| ApoB | Apolipoproteins | 6.18E-01 |
| ApoB/ApoA1 | Apolipoproteins | 4.80E-01 |
| Total cholines | Other lipids | 2.10E+00 |
| Citrate | Glycolysis related metabolites | 7.18E-02 |
| Clinical LDL-C | Cholesterol | 1.69E+00 |
| Creatinine | Fluid balance | 4.18E+01 |
| DHA | Fatty acids | 1.47E-01 |
| Glutamine | Amino acids | 5.12E-01 |
| Glycine | Amino acids | 1.71E-01 |
| Glycoprotein acetyls | Inflammation | 8.20E-01 |
| HDL-C | Cholesterol | 1.22E+00 |
| HDL-CE | Cholesteryl esters | 9.58E-01 |
| HDL-FC | Free cholesterol | 2.60E-01 |
| HDL-L | Total lipids | 2.73E+00 |
| HDL-P | Lipoprotein particle concentrations | 1.31E-02 |
| HDL-PL | Phospholipids | 1.41E+00 |
| HDL particle size | Lipoprotein particle sizes | 9.70E+00 |
| HDL-TG | Triglycerides | 9.79E-02 |
| Histidine | Amino acids | 7.17E-02 |
| Isoleucine | Amino acids | 5.32E-02 |
| LA | Fatty acids | 2.65E+00 |
| LDL-C | Cholesterol | 1.18E+00 |
| LDL-CE | Cholesteryl esters | 8.56E-01 |
| LDL-FC | Free cholesterol | 3.27E-01 |
| LDL-L | Total lipids | 1.71E+00 |
| LDL-P | Lipoprotein particle concentrations | 9.00E-04 |
| LDL-PL | Phospholipids | 4.33E-01 |
| LDL particle size | Lipoprotein particle sizes | 2.39E+01 |
| LDL-TG | Triglycerides | 9.83E-02 |
| Leucine | Amino acids | 1.00E-01 |
| MUFA | Fatty acids | 2.14E+00 |
| non-HDL-C | Cholesterol | 2.26E+00 |
| Omega-3 | Fatty acids | 2.29E-01 |
| Omega-6 | Fatty acids | 3.62E+00 |
| Omega-6/Omega-3 | Fatty acids | 1.79E+01 |
| Phenylalanine | Amino acids | 5.16E-02 |
| Phosphatidylcholines | Other lipids | 1.69E+00 |
| Phosphoglycerides | Other lipids | 1.83E+00 |
| PUFA | Fatty acids | 3.85E+00 |
| PUFA/MUFA | Fatty acids | 1.85E+00 |
| Pyruvate | Glycolysis related metabolites | 8.25E-02 |
| Remnant-C | Cholesterol | 1.08E+00 |
| SFA | Fatty acids | 3.19E+00 |
| Sphingomyelins | Other lipids | 3.87E-01 |
| TG/PG | Other lipids | 4.72E-01 |
| Total BCAA | Amino acids | 3.52E-01 |
| Total-C | Cholesterol | 3.48E+00 |
| Total-CE | Cholesteryl esters | 2.55E+00 |
| Total fatty acids | Fatty acids | 9.18E+00 |
| Total-FC | Free cholesterol | 9.36E-01 |
| Total-L | Total lipids | 6.71E+00 |
| Total-P | Lipoprotein particle concentrations | 1.44E-02 |
| Total-PL | Phospholipids | 2.37E+00 |
| Total triglycerides | Triglycerides | 8.67E-01 |
| Tyrosine | Amino acids | 6.86E-02 |
| Unsaturation | Fatty acids | 1.28E+00 |
| Valine | Amino acids | 1.98E-01 |
| VLDL-C | Cholesterol | 4.59E-01 |
| VLDL-CE | Cholesteryl esters | 2.75E-01 |
| VLDL-FC | Free cholesterol | 1.83E-01 |
| VLDL-L | Total lipids | 1.35E+00 |
| VLDL-P | Lipoprotein particle concentrations | 1.00E-04 |
| VLDL-PL | Phospholipids | 2.97E-01 |
| VLDL particle size | Lipoprotein particle sizes | 3.80E+01 |
| VLDL-TG | Triglycerides | 5.98E-01 |

**Supplementary Data D3.** Estimated differences in 71 metabolomic biomarkers between JIA and controls from a linear regression model with JIA status as the exposure and the biomarker as the outcome, adjusting age and sex of participants (n=90).

| Biomarker | estimate | se | P-value | group | P_adj_-value^a^ |
| --- | --- | --- | --- | --- | --- |
| Total-C | 1.08E-01 | 3.01E-01 | 7.21E-01 | Cholesterol | 8.00E-01 |
| non-HDL-C | -1.47E-01 | 3.00E-01 | 6.26E-01 | Cholesterol | 7.66E-01 |
| Remnant-C | -1.23E-01 | 3.01E-01 | 6.84E-01 | Cholesterol | 7.97E-01 |
| VLDL-C | -1.80E-01 | 3.03E-01 | 5.55E-01 | Cholesterol | 7.41E-01 |
| Clinical LDL-C | -2.36E-01 | 2.97E-01 | 4.29E-01 | Cholesterol | 6.76E-01 |
| LDL-C | -1.68E-01 | 3.00E-01 | 5.76E-01 | Cholesterol | 7.41E-01 |
| HDL-C | 6.09E-01 | 2.93E-01 | 4.07E-02 | Cholesterol | 2.21E-01 |
| Total fatty acids | 4.17E-01 | 3.02E-01 | 1.71E-01 | Fatty acids | 3.74E-01 |
| Unsaturation | 3.11E-01 | 3.00E-01 | 3.03E-01 | Fatty acids | 5.00E-01 |
| Omega-3 | 7.98E-01 | 2.92E-01 | 7.60E-03 | Fatty acids | 9.95E-02 |
| Omega-6 | 3.79E-01 | 3.01E-01 | 2.12E-01 | Fatty acids | 4.06E-01 |
| PUFA | 4.84E-01 | 2.99E-01 | 1.10E-01 | Fatty acids | 2.78E-01 |
| MUFA | 3.50E-01 | 3.03E-01 | 2.50E-01 | Fatty acids | 4.44E-01 |
| SFA | 3.55E-01 | 3.03E-01 | 2.45E-01 | Fatty acids | 4.44E-01 |
| LA | 3.82E-01 | 3.02E-01 | 2.09E-01 | Fatty acids | 4.06E-01 |
| DHA | 1.06E+00 | 2.80E-01 | 2.99E-04 | Fatty acids | 2.12E-02 |
| PUFA/MUFA | -1.16E-01 | 3.02E-01 | 7.02E-01 | Fatty acids | 7.97E-01 |
| Omega-6/Omega-3 | -7.87E-01 | 2.92E-01 | 8.41E-03 | Fatty acids | 9.95E-02 |
| Alanine | 5.52E-01 | 2.62E-01 | 3.82E-02 | Amino acids | 2.21E-01 |
| Glutamine | -4.06E-01 | 2.90E-01 | 1.65E-01 | Amino acids | 3.74E-01 |
| Glycine | -4.88E-02 | 3.04E-01 | 8.73E-01 | Amino acids | 8.98E-01 |
| Histidine | -5.25E-01 | 2.88E-01 | 7.14E-02 | Amino acids | 2.21E-01 |
| Total BCAA | 4.12E-01 | 2.75E-01 | 1.37E-01 | Amino acids | 3.25E-01 |
| Isoleucine | 4.32E-01 | 2.73E-01 | 1.17E-01 | Amino acids | 2.88E-01 |
| Leucine | 2.46E-01 | 2.73E-01 | 3.70E-01 | Amino acids | 5.97E-01 |
| Valine | 4.73E-01 | 2.79E-01 | 9.38E-02 | Amino acids | 2.47E-01 |
| Phenylalanine | 5.64E-01 | 2.70E-01 | 3.95E-02 | Amino acids | 2.21E-01 |
| Tyrosine | 5.56E-01 | 2.92E-01 | 6.04E-02 | Amino acids | 2.21E-01 |
| Phosphoglycerides | 5.50E-01 | 2.97E-01 | 6.75E-02 | Other lipids | 2.21E-01 |
| TG/PG | -5.73E-02 | 3.02E-01 | 8.50E-01 | Other lipids | 8.87E-01 |
| Total cholines | 5.36E-01 | 2.97E-01 | 7.48E-02 | Other lipids | 2.21E-01 |
| Phosphatidylcholines | 5.19E-01 | 2.97E-01 | 8.36E-02 | Other lipids | 2.37E-01 |
| Sphingomyelins | 5.66E-01 | 2.95E-01 | 5.85E-02 | Other lipids | 2.21E-01 |
| Total triglycerides | 1.71E-01 | 3.03E-01 | 5.74E-01 | Triglycerides | 7.41E-01 |
| VLDL-TG | 3.29E-02 | 3.04E-01 | 9.14E-01 | Triglycerides | 9.27E-01 |
| LDL-TG | 5.76E-01 | 2.98E-01 | 5.62E-02 | Triglycerides | 2.21E-01 |
| HDL-TG | 5.42E-01 | 2.97E-01 | 7.18E-02 | Triglycerides | 2.21E-01 |
| Total-PL | 4.07E-01 | 3.00E-01 | 1.78E-01 | Phospholipids | 3.74E-01 |
| VLDL-PL | -1.30E-01 | 3.04E-01 | 6.71E-01 | Phospholipids | 7.94E-01 |
| LDL-PL | -2.12E-01 | 2.98E-01 | 4.78E-01 | Phospholipids | 7.06E-01 |
| HDL-PL | 7.02E-01 | 2.89E-01 | 1.72E-02 | Phospholipids | 1.53E-01 |
| Total-CE | 1.13E-01 | 3.00E-01 | 7.07E-01 | Cholesteryl esters | 7.97E-01 |
| VLDL-CE | -1.86E-01 | 3.02E-01 | 5.39E-01 | Cholesteryl esters | 7.41E-01 |
| LDL-CE | -1.51E-01 | 3.01E-01 | 6.17E-01 | Cholesteryl esters | 7.66E-01 |
| HDL-CE | 5.65E-01 | 2.94E-01 | 5.74E-02 | Cholesteryl esters | 2.21E-01 |
| Total-FC | 8.88E-02 | 3.02E-01 | 7.70E-01 | Free cholesterol | 8.41E-01 |
| VLDL-FC | -1.67E-01 | 3.04E-01 | 5.84E-01 | Free cholesterol | 7.41E-01 |
| LDL-FC | -2.12E-01 | 2.96E-01 | 4.75E-01 | Free cholesterol | 7.06E-01 |
| HDL-FC | 7.36E-01 | 2.91E-01 | 1.33E-02 | Free cholesterol | 1.35E-01 |
| Total-L | 2.33E-01 | 3.03E-01 | 4.44E-01 | Total lipids | 6.85E-01 |
| VLDL-L | -7.36E-02 | 3.05E-01 | 8.10E-01 | Total lipids | 8.58E-01 |
| LDL-L | -1.40E-01 | 3.00E-01 | 6.42E-01 | Total lipids | 7.73E-01 |
| HDL-L | 6.90E-01 | 2.90E-01 | 1.97E-02 | Total lipids | 1.55E-01 |
| Total-P | 3.58E-01 | 2.96E-01 | 2.29E-01 | Lipoprotein particle concentrations | 4.28E-01 |
| VLDL-P | -1.86E-04 | 3.04E-01 | 1.00E+00 | Lipoprotein particle concentrations | 1.00E+00 |
| LDL-P | -1.80E-01 | 3.00E-01 | 5.50E-01 | Lipoprotein particle concentrations | 7.41E-01 |
| HDL-P | 3.98E-01 | 2.94E-01 | 1.79E-01 | Lipoprotein particle concentrations | 3.74E-01 |
| VLDL particle size | -3.38E-01 | 3.01E-01 | 2.65E-01 | Lipoprotein particle sizes | 4.59E-01 |
| LDL particle size | -7.36E-02 | 2.97E-01 | 8.05E-01 | Lipoprotein particle sizes | 8.58E-01 |
| HDL particle size | 8.20E-01 | 2.89E-01 | 5.71E-03 | Lipoprotein particle sizes | 9.95E-02 |
| ApoB | -1.86E-01 | 3.00E-01 | 5.38E-01 | Apolipoproteins | 7.41E-01 |
| ApoA1 | 5.88E-01 | 2.92E-01 | 4.69E-02 | Apolipoproteins | 2.21E-01 |
| ApoB/ApoA1 | -5.29E-01 | 2.91E-01 | 7.28E-02 | Apolipoproteins | 2.21E-01 |
| Pyruvate | 3.34E-01 | 3.13E-01 | 2.90E-01 | Glycolysis related metabolites | 4.90E-01 |
| Citrate | -4.01E-01 | 3.01E-01 | 1.86E-01 | Glycolysis related metabolites | 3.78E-01 |
| Acetate | -9.22E-01 | 2.61E-01 | 6.77E-04 | Ketone bodies | 2.40E-02 |
| Acetoacetate | -5.40E-01 | 2.77E-01 | 5.48E-02 | Ketone bodies | 2.21E-01 |
| Acetone | -5.49E-01 | 2.81E-01 | 5.37E-02 | Ketone bodies | 2.21E-01 |
| Creatinine | -1.40E-01 | 2.25E-01 | 5.36E-01 | Fluid balance | 7.41E-01 |
| Albumin | -5.14E-01 | 2.98E-01 | 8.75E-02 | Fluid balance | 2.39E-01 |
| Glycoprotein acetyls | 9.32E-01 | 2.87E-01 | 1.66E-03 | Inflammation | 3.93E-02 |

^a^ Adjusted by BH

**Supplementary Data D4.** Estimated differences in 71 metabolomic biomarkers between active JIA and controls from a linear regression model with JIA status as the exposure and the biomarker as the outcome, adjusting age and sex of participants (n=61).

| Biomarker | estimate | se | P-value | group | P*_adj_^a^* |
| --- | --- | --- | --- | --- | --- |
| Total-C | 2.26E-01 | 3.30E-01 | 4.95E-01 | Cholesterol | 6.86E-01 |
| non-HDL-C | 5.92E-02 | 3.30E-01 | 8.58E-01 | Cholesterol | 8.97E-01 |
| Remnant-C | 1.01E-01 | 3.30E-01 | 7.61E-01 | Cholesterol | 8.86E-01 |
| VLDL-C | 1.59E-01 | 3.30E-01 | 6.31E-01 | Cholesterol | 8.00E-01 |
| Clinical LDL-C | -7.87E-02 | 3.27E-01 | 8.11E-01 | Cholesterol | 8.86E-01 |
| LDL-C | 1.74E-02 | 3.29E-01 | 9.58E-01 | Cholesterol | 9.64E-01 |
| HDL-C | 4.50E-01 | 3.22E-01 | 1.68E-01 | Cholesterol | 3.22E-01 |
| Total fatty acids | 6.58E-01 | 3.24E-01 | 4.73E-02 | Fatty acids | 2.23E-01 |
| Unsaturation | 5.43E-02 | 3.34E-01 | 8.71E-01 | Fatty acids | 8.97E-01 |
| Omega-3 | 7.81E-01 | 3.18E-01 | 1.73E-02 | Fatty acids | 2.05E-01 |
| Omega-6 | 5.28E-01 | 3.27E-01 | 1.11E-01 | Fatty acids | 2.63E-01 |
| PUFA | 6.12E-01 | 3.25E-01 | 6.45E-02 | Fatty acids | 2.37E-01 |
| MUFA | 6.55E-01 | 3.24E-01 | 4.75E-02 | Fatty acids | 2.23E-01 |
| SFA | 6.19E-01 | 3.25E-01 | 6.23E-02 | Fatty acids | 2.37E-01 |
| LA | 5.30E-01 | 3.27E-01 | 1.11E-01 | Fatty acids | 2.63E-01 |
| DHA | 8.68E-01 | 3.15E-01 | 7.96E-03 | Fatty acids | 1.41E-01 |
| PUFA/MUFA | -4.60E-01 | 3.27E-01 | 1.65E-01 | Fatty acids | 3.22E-01 |
| Omega-6/Omega-3 | -7.31E-01 | 3.19E-01 | 2.57E-02 | Fatty acids | 2.23E-01 |
| Alanine | 4.78E-01 | 2.75E-01 | 8.75E-02 | Amino acids | 2.39E-01 |
| Glutamine | -5.59E-01 | 3.15E-01 | 8.11E-02 | Amino acids | 2.39E-01 |
| Glycine | -4.02E-01 | 3.09E-01 | 1.99E-01 | Amino acids | 3.62E-01 |
| Histidine | -7.84E-01 | 3.10E-01 | 1.42E-02 | Amino acids | 2.02E-01 |
| Total BCAA | 3.94E-01 | 2.90E-01 | 1.79E-01 | Amino acids | 3.34E-01 |
| Isoleucine | 4.59E-01 | 2.96E-01 | 1.26E-01 | Amino acids | 2.89E-01 |
| Leucine | 1.96E-01 | 2.85E-01 | 4.95E-01 | Amino acids | 6.86E-01 |
| Valine | 4.48E-01 | 2.95E-01 | 1.34E-01 | Amino acids | 2.89E-01 |
| Phenylalanine | 5.77E-01 | 2.85E-01 | 4.78E-02 | Amino acids | 2.23E-01 |
| Tyrosine | 6.41E-01 | 3.20E-01 | 5.02E-02 | Amino acids | 2.23E-01 |
| Phosphoglycerides | 5.97E-01 | 3.23E-01 | 7.01E-02 | Other lipids | 2.37E-01 |
| TG/PG | 2.56E-01 | 3.32E-01 | 4.44E-01 | Other lipids | 6.43E-01 |
| Total cholines | 5.82E-01 | 3.24E-01 | 7.72E-02 | Other lipids | 2.38E-01 |
| Phosphatidylcholines | 5.44E-01 | 3.24E-01 | 9.80E-02 | Other lipids | 2.48E-01 |
| Sphingomyelins | 6.87E-01 | 3.20E-01 | 3.61E-02 | Other lipids | 2.23E-01 |
| Total triglycerides | 4.78E-01 | 3.29E-01 | 1.52E-01 | Triglycerides | 3.17E-01 |
| VLDL-TG | 3.20E-01 | 3.33E-01 | 3.40E-01 | Triglycerides | 5.40E-01 |
| LDL-TG | 9.33E-01 | 3.11E-01 | 3.96E-03 | Triglycerides | 9.37E-02 |
| HDL-TG | 6.64E-01 | 3.21E-01 | 4.30E-02 | Triglycerides | 2.23E-01 |
| Total-PL | 5.00E-01 | 3.26E-01 | 1.31E-01 | Phospholipids | 2.89E-01 |
| VLDL-PL | 2.24E-01 | 3.32E-01 | 5.03E-01 | Phospholipids | 6.86E-01 |
| LDL-PL | -1.50E-02 | 3.28E-01 | 9.64E-01 | Phospholipids | 9.64E-01 |
| HDL-PL | 5.70E-01 | 3.16E-01 | 7.70E-02 | Phospholipids | 2.38E-01 |
| Total-CE | 2.12E-01 | 3.29E-01 | 5.22E-01 | Cholesteryl esters | 6.87E-01 |
| VLDL-CE | 1.37E-01 | 3.29E-01 | 6.80E-01 | Cholesteryl esters | 8.47E-01 |
| LDL-CE | 5.39E-02 | 3.30E-01 | 8.71E-01 | Cholesteryl esters | 8.97E-01 |
| HDL-CE | 3.94E-01 | 3.23E-01 | 2.27E-01 | Cholesteryl esters | 3.93E-01 |
| Total-FC | 2.59E-01 | 3.31E-01 | 4.37E-01 | Free cholesterol | 6.43E-01 |
| VLDL-FC | 1.83E-01 | 3.32E-01 | 5.84E-01 | Free cholesterol | 7.54E-01 |
| LDL-FC | -8.81E-02 | 3.27E-01 | 7.88E-01 | Free cholesterol | 8.86E-01 |
| HDL-FC | 6.23E-01 | 3.19E-01 | 5.58E-02 | Free cholesterol | 2.33E-01 |
| Total-L | 4.13E-01 | 3.30E-01 | 2.16E-01 | Total lipids | 3.83E-01 |
| VLDL-L | 2.60E-01 | 3.33E-01 | 4.38E-01 | Total lipids | 6.43E-01 |
| LDL-L | 6.34E-02 | 3.30E-01 | 8.48E-01 | Total lipids | 8.97E-01 |
| HDL-L | 5.53E-01 | 3.18E-01 | 8.73E-02 | Total lipids | 2.39E-01 |
| Total-P | 3.05E-01 | 3.24E-01 | 3.50E-01 | Lipoprotein particle concentrations | 5.40E-01 |
| VLDL-P | 3.61E-01 | 3.30E-01 | 2.78E-01 | Lipoprotein particle concentrations | 4.59E-01 |
| LDL-P | 1.00E-01 | 3.29E-01 | 7.62E-01 | Lipoprotein particle concentrations | 8.86E-01 |
| HDL-P | 3.05E-01 | 3.22E-01 | 3.48E-01 | Lipoprotein particle concentrations | 5.40E-01 |
| VLDL particle size | -9.48E-02 | 3.35E-01 | 7.78E-01 | Lipoprotein particle sizes | 8.86E-01 |
| LDL particle size | -1.26E-01 | 3.28E-01 | 7.01E-01 | Lipoprotein particle sizes | 8.58E-01 |
| HDL particle size | 6.51E-01 | 3.18E-01 | 4.50E-02 | Lipoprotein particle sizes | 2.23E-01 |
| ApoB | 9.05E-02 | 3.29E-01 | 7.84E-01 | Apolipoproteins | 8.86E-01 |
| ApoA1 | 4.58E-01 | 3.19E-01 | 1.57E-01 | Apolipoproteins | 3.18E-01 |
| ApoB/ApoA1 | -2.07E-01 | 3.20E-01 | 5.21E-01 | Apolipoproteins | 6.87E-01 |
| Pyruvate | 3.81E-01 | 3.34E-01 | 2.58E-01 | Glycolysis related metabolites | 4.36E-01 |
| Citrate | -6.52E-01 | 3.20E-01 | 4.62E-02 | Glycolysis related metabolites | 2.23E-01 |
| Acetoacetate | -4.98E-01 | 2.91E-01 | 9.24E-02 | Ketone bodies | 2.43E-01 |
| Acetone | -6.39E-01 | 2.97E-01 | 3.55E-02 | Ketone bodies | 2.23E-01 |
| Creatinine | -6.27E-02 | 2.48E-01 | 8.01E-01 | Fluid balance | 8.86E-01 |
| Albumin | -6.04E-01 | 3.25E-01 | 6.82E-02 | Fluid balance | 2.37E-01 |
| Acetate | -9.16E-01 | 2.54E-01 | 6.66E-04 | Ketone bodies | 2.36E-02 |
| Glycoprotein acetyls | 1.26E+00 | 2.90E-01 | 5.85E-05 | Inflammation | 4.15E-03 |

^a^ Adjusted by BH

**Supplementary Data D5.** Estimated differences in 71 metabolomic biomarkers between inactive JIA and controls from a linear regression model with JIA status as the exposure and the biomarker as the outcome, adjusting age and sex of participants (n=31).

| Biomarker | estimate | se | p-value | group | P*_adj_^a^* |
| --- | --- | --- | --- | --- | --- |
| Total-C | -1.63E-01 | 4.93E-01 | 7.43E-01 | Cholesterol | 8.74E-01 |
| non-HDL-C | -5.35E-01 | 4.85E-01 | 2.80E-01 | Cholesterol | 5.77E-01 |
| Remnant-C | -5.61E-01 | 4.85E-01 | 2.57E-01 | Cholesterol | 5.77E-01 |
| VLDL-C | -7.52E-01 | 4.70E-01 | 1.22E-01 | Cholesterol | 3.93E-01 |
| Clinical LDL-C | -6.24E-01 | 4.81E-01 | 2.05E-01 | Cholesterol | 5.21E-01 |
| LDL-C | -5.01E-01 | 4.86E-01 | 3.12E-01 | Cholesterol | 5.77E-01 |
| HDL-C | 8.55E-01 | 4.42E-01 | 6.35E-02 | Cholesterol | 2.90E-01 |
| Total fatty acids | -2.16E-01 | 4.94E-01 | 6.65E-01 | Fatty acids | 8.47E-01 |
| Unsaturation | 4.31E-01 | 4.85E-01 | 3.82E-01 | Fatty acids | 6.03E-01 |
| Omega-3 | 8.89E-01 | 4.63E-01 | 6.54E-02 | Fatty acids | 2.90E-01 |
| Omega-6 | -2.00E-01 | 4.85E-01 | 6.84E-01 | Fatty acids | 8.47E-01 |
| PUFA | -2.30E-02 | 4.90E-01 | 9.63E-01 | Fatty acids | 9.63E-01 |
| MUFA | -3.22E-01 | 4.93E-01 | 5.20E-01 | Fatty acids | 7.35E-01 |
| SFA | -2.95E-01 | 4.93E-01 | 5.55E-01 | Fatty acids | 7.51E-01 |
| LA | 5.10E-02 | 4.82E-01 | 9.17E-01 | Fatty acids | 9.63E-01 |
| DHA | 1.35E+00 | 4.23E-01 | 3.46E-03 | Fatty acids | 4.09E-02 |
| PUFA/MUFA | 4.68E-01 | 4.85E-01 | 3.43E-01 | Fatty acids | 5.94E-01 |
| Omega-6/Omega-3 | -1.03E+00 | 4.45E-01 | 2.87E-02 | Fatty acids | 2.26E-01 |
| Alanine | 1.57E+00 | 3.51E-01 | 1.26E-04 | Amino acids | 8.91E-03 |
| Glutamine | 6.07E-01 | 4.75E-01 | 2.13E-01 | Amino acids | 5.21E-01 |
| Glycine | 5.04E-01 | 4.69E-01 | 2.92E-01 | Amino acids | 5.77E-01 |
| Histidine | 9.68E-01 | 4.57E-01 | 4.33E-02 | Amino acids | 2.79E-01 |
| Total BCAA | 2.74E-01 | 4.66E-01 | 5.61E-01 | Amino acids | 7.51E-01 |
| Isoleucine | 2.97E-01 | 4.43E-01 | 5.08E-01 | Amino acids | 7.35E-01 |
| Leucine | 1.49E-01 | 4.64E-01 | 7.51E-01 | Amino acids | 8.74E-01 |
| Valine | 3.04E-01 | 4.75E-01 | 5.28E-01 | Amino acids | 7.35E-01 |
| Phenylalanine | 1.23E+00 | 3.76E-01 | 2.85E-03 | Amino acids | 4.09E-02 |
| Tyrosine | 1.12E+00 | 4.17E-01 | 1.20E-02 | Amino acids | 1.21E-01 |
| Phosphoglycerides | 3.74E-01 | 4.77E-01 | 4.40E-01 | Other lipids | 6.67E-01 |
| TG/PG | -1.22E-01 | 4.85E-01 | 8.03E-01 | Other lipids | 9.05E-01 |
| Total cholines | 3.74E-01 | 4.79E-01 | 4.42E-01 | Other lipids | 6.67E-01 |
| Phosphatidylcholines | 5.62E-01 | 4.67E-01 | 2.39E-01 | Other lipids | 5.67E-01 |
| Sphingomyelins | -2.99E-02 | 4.96E-01 | 9.52E-01 | Other lipids | 9.63E-01 |
| Total triglycerides | 5.90E-02 | 4.92E-01 | 9.05E-01 | Triglycerides | 9.63E-01 |
| VLDL-TG | -7.15E-02 | 4.89E-01 | 8.85E-01 | Triglycerides | 9.63E-01 |
| LDL-TG | 1.73E-01 | 4.92E-01 | 7.28E-01 | Triglycerides | 8.74E-01 |
| HDL-TG | 9.04E-01 | 4.62E-01 | 6.09E-02 | Triglycerides | 2.90E-01 |
| Total-PL | 2.00E-01 | 4.87E-01 | 6.85E-01 | Phospholipids | 8.47E-01 |
| VLDL-PL | -6.27E-01 | 4.76E-01 | 1.99E-01 | Phospholipids | 5.21E-01 |
| LDL-PL | -6.31E-01 | 4.80E-01 | 1.99E-01 | Phospholipids | 5.21E-01 |
| HDL-PL | 8.52E-01 | 4.35E-01 | 6.05E-02 | Phospholipids | 2.90E-01 |
| Total-CE | -1.45E-01 | 4.93E-01 | 7.71E-01 | Cholesteryl esters | 8.82E-01 |
| VLDL-CE | -7.58E-01 | 4.71E-01 | 1.19E-01 | Cholesteryl esters | 3.93E-01 |
| LDL-CE | -4.96E-01 | 4.87E-01 | 3.17E-01 | Cholesteryl esters | 5.77E-01 |
| HDL-CE | 8.16E-01 | 4.44E-01 | 7.69E-02 | Cholesteryl esters | 3.21E-01 |
| Total-FC | -2.11E-01 | 4.95E-01 | 6.72E-01 | Free cholesterol | 8.47E-01 |
| VLDL-FC | -7.21E-01 | 4.71E-01 | 1.37E-01 | Free cholesterol | 4.23E-01 |
| LDL-FC | -5.01E-01 | 4.85E-01 | 3.11E-01 | Free cholesterol | 5.77E-01 |
| HDL-FC | 9.63E-01 | 4.38E-01 | 3.66E-02 | Free cholesterol | 2.60E-01 |
| Total-L | -2.61E-02 | 4.95E-01 | 9.58E-01 | Total lipids | 9.63E-01 |
| VLDL-L | -4.51E-01 | 4.83E-01 | 3.59E-01 | Total lipids | 6.03E-01 |
| LDL-L | -5.15E-01 | 4.86E-01 | 2.98E-01 | Total lipids | 5.77E-01 |
| HDL-L | 8.98E-01 | 4.35E-01 | 4.88E-02 | Total lipids | 2.89E-01 |
| Total-P | 3.03E-01 | 4.69E-01 | 5.24E-01 | Lipoprotein particle concentrations | 7.35E-01 |
| VLDL-P | -4.95E-01 | 4.83E-01 | 3.15E-01 | Lipoprotein particle concentrations | 5.77E-01 |
| LDL-P | -6.64E-01 | 4.78E-01 | 1.76E-01 | Lipoprotein particle concentrations | 4.99E-01 |
| HDL-P | 4.09E-01 | 4.61E-01 | 3.82E-01 | Lipoprotein particle concentrations | 6.03E-01 |
| VLDL particle size | -5.21E-01 | 4.75E-01 | 2.82E-01 | Lipoprotein particle sizes | 5.77E-01 |
| LDL particle size | -2.90E-02 | 4.82E-01 | 9.52E-01 | Lipoprotein particle sizes | 9.63E-01 |
| HDL particle size | 1.45E+00 | 3.95E-01 | 1.03E-03 | Lipoprotein particle sizes | 2.78E-02 |
| ApoB | -6.76E-01 | 4.78E-01 | 1.69E-01 | Apolipoproteins | 4.99E-01 |
| ApoA1 | 7.67E-01 | 4.43E-01 | 9.49E-02 | Apolipoproteins | 3.37E-01 |
| ApoB/ApoA1 | -1.07E+00 | 4.31E-01 | 1.96E-02 | Apolipoproteins | 1.74E-01 |
| Pyruvate | 4.28E-01 | 4.79E-01 | 3.79E-01 | Glycolysis related metabolites | 6.03E-01 |
| Citrate | 1.96E-01 | 4.90E-01 | 6.92E-01 | Glycolysis related metabolites | 8.47E-01 |
| Acetate | -7.79E-01 | 4.48E-01 | 9.34E-02 | Ketone bodies | 3.37E-01 |
| Acetoacetate | -1.39E+00 | 3.83E-01 | 1.18E-03 | Ketone bodies | 2.78E-02 |
| Acetone | -1.29E+00 | 4.01E-01 | 3.33E-03 | Ketone bodies | 4.09E-02 |
| Creatinine | -6.07E-01 | 3.40E-01 | 8.58E-02 | Fluid balance | 3.37E-01 |
| Albumin | 2.52E-02 | 4.85E-01 | 9.59E-01 | Fluid balance | 9.63E-01 |
| Glycoprotein acetyls | -4.85E-01 | 4.85E-01 | 3.27E-01 | Inflammation | 5.80E-01 |

^a^ Adjusted by BH

**Supplementary Data D6.** Estimated differences in 71 metabolomic biomarkers between JIA subtypes and controls from a linear regression model with JIA subtypes status as the exposure and the biomarker as the outcome, adjusting age and sex of participants (n=90).

| Biomarker | Subtypes | estimate | se | P-value | P*_adj_ ^a^* |
| --- | --- | --- | --- | --- | --- |
| Total-C | sJIA | 6.23E-01 | 3.90E-01 | 1.19E-01 | 2.23E-01 |
| Total-C | pJIA(RF+) | -3.54E-01 | 6.80E-01 | 6.07E-01 | 8.22E-01 |
| Total-C | oJIA | -3.26E-01 | 4.55E-01 | 4.79E-01 | 8.79E-01 |
| Total-C | pJIA(RF-) | -2.29E-01 | 4.23E-01 | 5.93E-01 | 9.68E-01 |
| non-HDL-C | pJIA(RF+) | -6.46E-01 | 6.35E-01 | 3.18E-01 | 6.68E-01 |
| non-HDL-C | oJIA | -5.32E-01 | 4.45E-01 | 2.41E-01 | 7.70E-01 |
| non-HDL-C | sJIA | 1.73E-01 | 4.01E-01 | 6.68E-01 | 8.79E-01 |
| non-HDL-C | pJIA(RF-) | -2.65E-01 | 4.24E-01 | 5.36E-01 | 9.68E-01 |
| Remnant-C | pJIA(RF+) | -5.70E-01 | 6.42E-01 | 3.82E-01 | 7.53E-01 |
| Remnant-C | oJIA | -5.16E-01 | 4.46E-01 | 2.55E-01 | 7.70E-01 |
| Remnant-C | sJIA | 2.01E-01 | 4.01E-01 | 6.20E-01 | 8.46E-01 |
| Remnant-C | pJIA(RF-) | -1.54E-01 | 4.26E-01 | 7.20E-01 | 9.68E-01 |
| VLDL-C | pJIA(RF+) | -7.24E-01 | 6.35E-01 | 2.64E-01 | 6.68E-01 |
| VLDL-C | oJIA | -5.83E-01 | 4.38E-01 | 1.92E-01 | 7.70E-01 |
| VLDL-C | pJIA(RF-) | 4.18E-02 | 4.26E-01 | 9.22E-01 | 9.68E-01 |
| VLDL-C | sJIA | -2.37E-02 | 3.92E-01 | 9.52E-01 | 9.80E-01 |
| Clinical LDL-C | pJIA(RF+) | -7.38E-01 | 6.22E-01 | 2.45E-01 | 6.68E-01 |
| Clinical LDL-C | oJIA | -5.30E-01 | 4.46E-01 | 2.43E-01 | 7.70E-01 |
| Clinical LDL-C | sJIA | 4.46E-02 | 4.01E-01 | 9.12E-01 | 9.62E-01 |
| Clinical LDL-C | pJIA(RF-) | -4.24E-01 | 4.20E-01 | 3.20E-01 | 9.68E-01 |
| LDL-C | pJIA(RF+) | -6.95E-01 | 6.33E-01 | 2.82E-01 | 6.68E-01 |
| LDL-C | oJIA | -5.40E-01 | 4.46E-01 | 2.34E-01 | 7.70E-01 |
| LDL-C | sJIA | 1.40E-01 | 4.01E-01 | 7.28E-01 | 9.03E-01 |
| LDL-C | pJIA(RF-) | -3.53E-01 | 4.20E-01 | 4.07E-01 | 9.68E-01 |
| HDL-C | sJIA | 1.16E+00 | 3.30E-01 | 1.25E-03 | 9.85E-03 |
| HDL-C | pJIA(RF+) | 7.47E-01 | 7.11E-01 | 3.03E-01 | 6.68E-01 |
| HDL-C | oJIA | 4.31E-01 | 4.33E-01 | 3.26E-01 | 8.00E-01 |
| HDL-C | pJIA(RF-) | -2.44E-02 | 4.24E-01 | 9.54E-01 | 9.68E-01 |
| Total triglycerides | sJIA | 3.25E-01 | 3.90E-01 | 4.10E-01 | 6.06E-01 |
| Total triglycerides | pJIA(RF+) | 3.06E-01 | 7.28E-01 | 6.77E-01 | 8.59E-01 |
| Total triglycerides | oJIA | -2.13E-01 | 4.57E-01 | 6.44E-01 | 8.84E-01 |
| Total triglycerides | pJIA(RF-) | 3.38E-01 | 3.95E-01 | 3.99E-01 | 9.68E-01 |
| VLDL-TG | oJIA | -3.43E-01 | 4.54E-01 | 4.56E-01 | 8.79E-01 |
| VLDL-TG | sJIA | 1.08E-01 | 3.93E-01 | 7.85E-01 | 9.03E-01 |
| VLDL-TG | pJIA(RF+) | 9.15E-02 | 7.28E-01 | 9.01E-01 | 9.67E-01 |
| VLDL-TG | pJIA(RF-) | 1.94E-01 | 3.97E-01 | 6.29E-01 | 9.68E-01 |
| LDL-TG | sJIA | 1.00E+00 | 3.66E-01 | 9.76E-03 | 4.12E-02 |
| LDL-TG | pJIA(RF-) | 7.27E-01 | 3.93E-01 | 7.38E-02 | 3.74E-01 |
| LDL-TG | pJIA(RF+) | 7.26E-01 | 7.18E-01 | 3.20E-01 | 6.68E-01 |
| LDL-TG | oJIA | 2.24E-01 | 4.44E-01 | 6.17E-01 | 8.84E-01 |
| HDL-TG | sJIA | 7.03E-01 | 3.79E-01 | 7.21E-02 | 1.51E-01 |
| HDL-TG | pJIA(RF-) | 8.57E-01 | 3.88E-01 | 3.46E-02 | 3.74E-01 |
| HDL-TG | pJIA(RF+) | 1.19E+00 | 6.86E-01 | 9.26E-02 | 5.10E-01 |
| HDL-TG | oJIA | 2.66E-01 | 4.53E-01 | 5.62E-01 | 8.79E-01 |
| Total-PL | sJIA | 9.82E-01 | 3.65E-01 | 1.07E-02 | 4.12E-02 |
| Total-PL | pJIA(RF+) | 1.86E-01 | 7.16E-01 | 7.97E-01 | 9.28E-01 |
| Total-PL | oJIA | -3.55E-02 | 4.56E-01 | 9.38E-01 | 9.67E-01 |
| Total-PL | pJIA(RF-) | -2.63E-02 | 4.20E-01 | 9.50E-01 | 9.68E-01 |
| VLDL-PL | oJIA | -5.59E-01 | 4.42E-01 | 2.14E-01 | 7.70E-01 |
| VLDL-PL | pJIA(RF+) | -4.93E-01 | 6.78E-01 | 4.73E-01 | 7.94E-01 |
| VLDL-PL | pJIA(RF-) | 1.31E-01 | 4.17E-01 | 7.55E-01 | 9.68E-01 |
| VLDL-PL | sJIA | -4.80E-03 | 3.93E-01 | 9.90E-01 | 9.90E-01 |
| LDL-PL | pJIA(RF+) | -7.53E-01 | 6.22E-01 | 2.36E-01 | 6.68E-01 |
| LDL-PL | oJIA | -5.41E-01 | 4.44E-01 | 2.32E-01 | 7.70E-01 |
| LDL-PL | sJIA | 8.82E-02 | 4.00E-01 | 8.27E-01 | 9.03E-01 |
| LDL-PL | pJIA(RF-) | -3.82E-01 | 4.21E-01 | 3.71E-01 | 9.68E-01 |
| HDL-PL | sJIA | 1.20E+00 | 3.19E-01 | 6.23E-04 | 7.37E-03 |
| HDL-PL | pJIA(RF+) | 1.05E+00 | 6.91E-01 | 1.42E-01 | 6.68E-01 |
| HDL-PL | oJIA | 4.98E-01 | 4.18E-01 | 2.43E-01 | 7.70E-01 |
| HDL-PL | pJIA(RF-) | 1.11E-01 | 4.19E-01 | 7.93E-01 | 9.68E-01 |
| Total-CE | sJIA | 6.39E-01 | 3.89E-01 | 1.09E-01 | 2.10E-01 |
| Total-CE | pJIA(RF+) | -3.48E-01 | 6.82E-01 | 6.14E-01 | 8.22E-01 |
| Total-CE | oJIA | -3.07E-01 | 4.55E-01 | 5.04E-01 | 8.79E-01 |
| Total-CE | pJIA(RF-) | -2.58E-01 | 4.22E-01 | 5.46E-01 | 9.68E-01 |
| VLDL-CE | pJIA(RF+) | -7.37E-01 | 6.29E-01 | 2.51E-01 | 6.68E-01 |
| VLDL-CE | oJIA | -5.78E-01 | 4.38E-01 | 1.95E-01 | 7.70E-01 |
| VLDL-CE | pJIA(RF-) | 3.84E-02 | 4.28E-01 | 9.29E-01 | 9.68E-01 |
| VLDL-CE | sJIA | -1.16E-02 | 3.93E-01 | 9.77E-01 | 9.90E-01 |
| LDL-CE | pJIA(RF+) | -6.83E-01 | 6.34E-01 | 2.91E-01 | 6.68E-01 |
| LDL-CE | oJIA | -5.42E-01 | 4.46E-01 | 2.33E-01 | 7.70E-01 |
| LDL-CE | sJIA | 1.48E-01 | 4.01E-01 | 7.14E-01 | 9.03E-01 |
| LDL-CE | pJIA(RF-) | -3.19E-01 | 4.20E-01 | 4.54E-01 | 9.68E-01 |
| HDL-CE | sJIA | 1.10E+00 | 3.35E-01 | 2.33E-03 | 1.59E-02 |
| HDL-CE | pJIA(RF+) | 6.82E-01 | 7.15E-01 | 3.48E-01 | 7.06E-01 |
| HDL-CE | oJIA | 4.22E-01 | 4.34E-01 | 3.38E-01 | 8.00E-01 |
| HDL-CE | pJIA(RF-) | -9.14E-02 | 4.24E-01 | 8.31E-01 | 9.68E-01 |
| Total-FC | sJIA | 5.68E-01 | 3.93E-01 | 1.57E-01 | 2.71E-01 |
| Total-FC | pJIA(RF+) | -3.63E-01 | 6.76E-01 | 5.95E-01 | 8.22E-01 |
| Total-FC | oJIA | -3.73E-01 | 4.53E-01 | 4.16E-01 | 8.79E-01 |
| Total-FC | pJIA(RF-) | -1.47E-01 | 4.25E-01 | 7.32E-01 | 9.68E-01 |
| VLDL-FC | pJIA(RF+) | -6.60E-01 | 6.52E-01 | 3.20E-01 | 6.68E-01 |
| VLDL-FC | oJIA | -5.75E-01 | 4.39E-01 | 2.00E-01 | 7.70E-01 |
| VLDL-FC | sJIA | -4.16E-02 | 3.92E-01 | 9.16E-01 | 9.62E-01 |
| VLDL-FC | pJIA(RF-) | 4.49E-02 | 4.20E-01 | 9.16E-01 | 9.68E-01 |
| LDL-FC | pJIA(RF+) | -7.07E-01 | 6.33E-01 | 2.74E-01 | 6.68E-01 |
| LDL-FC | oJIA | -5.26E-01 | 4.47E-01 | 2.48E-01 | 7.70E-01 |
| LDL-FC | sJIA | 1.10E-01 | 4.00E-01 | 7.85E-01 | 9.03E-01 |
| LDL-FC | pJIA(RF-) | -4.33E-01 | 4.19E-01 | 3.10E-01 | 9.68E-01 |
| HDL-FC | sJIA | 1.32E+00 | 3.18E-01 | 2.01E-04 | 5.33E-03 |
| HDL-FC | pJIA(RF+) | 9.47E-01 | 6.97E-01 | 1.85E-01 | 6.68E-01 |
| HDL-FC | oJIA | 4.51E-01 | 4.29E-01 | 3.01E-01 | 8.00E-01 |
| HDL-FC | pJIA(RF-) | 2.14E-01 | 4.23E-01 | 6.17E-01 | 9.68E-01 |
| Total-L | sJIA | 7.23E-01 | 3.84E-01 | 6.75E-02 | 1.45E-01 |
| Total-L | oJIA | -2.45E-01 | 4.56E-01 | 5.94E-01 | 8.79E-01 |
| Total-L | pJIA(RF+) | -1.36E-01 | 6.98E-01 | 8.47E-01 | 9.61E-01 |
| Total-L | pJIA(RF-) | -6.63E-02 | 4.19E-01 | 8.75E-01 | 9.68E-01 |
| VLDL-L | oJIA | -5.06E-01 | 4.48E-01 | 2.67E-01 | 7.70E-01 |
| VLDL-L | pJIA(RF+) | -3.20E-01 | 7.02E-01 | 6.52E-01 | 8.41E-01 |
| VLDL-L | sJIA | 3.92E-02 | 3.92E-01 | 9.21E-01 | 9.62E-01 |
| VLDL-L | pJIA(RF-) | 1.40E-01 | 4.08E-01 | 7.33E-01 | 9.68E-01 |
| LDL-L | pJIA(RF+) | -6.61E-01 | 6.38E-01 | 3.08E-01 | 6.68E-01 |
| LDL-L | oJIA | -5.18E-01 | 4.46E-01 | 2.53E-01 | 7.70E-01 |
| LDL-L | sJIA | 1.86E-01 | 4.01E-01 | 6.45E-01 | 8.64E-01 |
| LDL-L | pJIA(RF-) | -3.20E-01 | 4.22E-01 | 4.54E-01 | 9.68E-01 |
| HDL-L | sJIA | 1.21E+00 | 3.21E-01 | 5.86E-04 | 7.37E-03 |
| HDL-L | pJIA(RF+) | 9.59E-01 | 6.98E-01 | 1.80E-01 | 6.68E-01 |
| HDL-L | oJIA | 4.94E-01 | 4.24E-01 | 2.52E-01 | 7.70E-01 |
| HDL-L | pJIA(RF-) | 9.30E-02 | 4.21E-01 | 8.27E-01 | 9.68E-01 |
| Total-P | sJIA | 8.83E-01 | 3.59E-01 | 1.90E-02 | 5.39E-02 |
| Total-P | oJIA | 1.28E-01 | 4.48E-01 | 7.76E-01 | 9.26E-01 |
| Total-P | pJIA(RF+) | 1.35E-01 | 7.18E-01 | 8.53E-01 | 9.61E-01 |
| Total-P | pJIA(RF-) | -3.12E-01 | 4.12E-01 | 4.55E-01 | 9.68E-01 |
| VLDL-P | oJIA | -5.31E-01 | 4.44E-01 | 2.40E-01 | 7.70E-01 |
| VLDL-P | pJIA(RF+) | -4.13E-01 | 6.72E-01 | 5.44E-01 | 8.04E-01 |
| VLDL-P | sJIA | 1.99E-01 | 3.93E-01 | 6.15E-01 | 8.46E-01 |
| VLDL-P | pJIA(RF-) | 3.07E-01 | 4.18E-01 | 4.68E-01 | 9.68E-01 |
| LDL-P | pJIA(RF+) | -8.24E-01 | 6.27E-01 | 2.00E-01 | 6.68E-01 |
| LDL-P | oJIA | -5.85E-01 | 4.36E-01 | 1.89E-01 | 7.70E-01 |
| LDL-P | sJIA | 9.52E-02 | 3.92E-01 | 8.09E-01 | 9.03E-01 |
| LDL-P | pJIA(RF-) | -2.08E-01 | 4.26E-01 | 6.29E-01 | 9.68E-01 |
| HDL-P | sJIA | 8.96E-01 | 3.52E-01 | 1.55E-02 | 4.58E-02 |
| HDL-P | pJIA(RF+) | 2.73E-01 | 7.23E-01 | 7.08E-01 | 8.82E-01 |
| HDL-P | oJIA | 2.19E-01 | 4.42E-01 | 6.24E-01 | 8.84E-01 |
| HDL-P | pJIA(RF-) | -3.11E-01 | 4.11E-01 | 4.55E-01 | 9.68E-01 |
| VLDL particle size | sJIA | -4.86E-01 | 3.84E-01 | 2.13E-01 | 3.61E-01 |
| VLDL particle size | oJIA | -4.53E-01 | 4.51E-01 | 3.22E-01 | 8.00E-01 |
| VLDL particle size | pJIA(RF+) | -4.12E-01 | 7.19E-01 | 5.72E-01 | 8.22E-01 |
| VLDL particle size | pJIA(RF-) | -2.96E-01 | 3.96E-01 | 4.60E-01 | 9.68E-01 |
| LDL particle size | sJIA | 3.13E-01 | 3.88E-01 | 4.24E-01 | 6.15E-01 |
| LDL particle size | pJIA(RF-) | -5.80E-01 | 4.10E-01 | 1.67E-01 | 6.98E-01 |
| LDL particle size | oJIA | -1.00E-01 | 4.34E-01 | 8.19E-01 | 9.38E-01 |
| LDL particle size | pJIA(RF+) | 9.80E-03 | 7.06E-01 | 9.89E-01 | 9.89E-01 |
| HDL particle size | sJIA | 1.30E+00 | 3.26E-01 | 3.24E-04 | 5.75E-03 |
| HDL particle size | pJIA(RF-) | 7.61E-01 | 4.08E-01 | 7.18E-02 | 3.74E-01 |
| HDL particle size | pJIA(RF+) | 1.29E+00 | 5.92E-01 | 3.83E-02 | 4.11E-01 |
| HDL particle size | oJIA | 6.84E-01 | 4.25E-01 | 1.17E-01 | 7.70E-01 |
| Phosphoglycerides | sJIA | 1.03E+00 | 3.57E-01 | 6.56E-03 | 3.11E-02 |
| Phosphoglycerides | pJIA(RF+) | 5.38E-01 | 7.20E-01 | 4.61E-01 | 7.94E-01 |
| Phosphoglycerides | oJIA | 1.74E-01 | 4.50E-01 | 7.01E-01 | 8.90E-01 |
| Phosphoglycerides | pJIA(RF-) | 1.55E-01 | 4.16E-01 | 7.12E-01 | 9.68E-01 |
| TG/PG | oJIA | -2.82E-01 | 4.55E-01 | 5.40E-01 | 8.79E-01 |
| TG/PG | sJIA | -1.03E-01 | 3.90E-01 | 7.93E-01 | 9.03E-01 |
| TG/PG | pJIA(RF+) | 4.88E-02 | 7.32E-01 | 9.47E-01 | 9.67E-01 |
| TG/PG | pJIA(RF-) | 2.77E-01 | 4.10E-01 | 5.04E-01 | 9.68E-01 |
| Total cholines | sJIA | 1.05E+00 | 3.56E-01 | 5.41E-03 | 2.75E-02 |
| Total cholines | pJIA(RF+) | 4.54E-01 | 7.22E-01 | 5.35E-01 | 8.04E-01 |
| Total cholines | oJIA | 1.35E-01 | 4.53E-01 | 7.67E-01 | 9.26E-01 |
| Total cholines | pJIA(RF-) | 1.26E-01 | 4.19E-01 | 7.65E-01 | 9.68E-01 |
| Phosphatidylcholines | sJIA | 9.59E-01 | 3.61E-01 | 1.16E-02 | 4.12E-02 |
| Phosphatidylcholines | pJIA(RF+) | 6.20E-01 | 7.17E-01 | 3.94E-01 | 7.56E-01 |
| Phosphatidylcholines | oJIA | 6.41E-02 | 4.49E-01 | 8.87E-01 | 9.67E-01 |
| Phosphatidylcholines | pJIA(RF-) | 2.41E-01 | 4.16E-01 | 5.66E-01 | 9.68E-01 |
| Sphingomyelins | sJIA | 1.24E+00 | 3.45E-01 | 9.79E-04 | 8.69E-03 |
| Sphingomyelins | oJIA | 1.70E-01 | 4.57E-01 | 7.13E-01 | 8.90E-01 |
| Sphingomyelins | pJIA(RF+) | 2.21E-01 | 7.23E-01 | 7.62E-01 | 9.14E-01 |
| Sphingomyelins | pJIA(RF-) | 4.61E-02 | 4.26E-01 | 9.14E-01 | 9.68E-01 |
| ApoB | pJIA(RF+) | -7.44E-01 | 6.30E-01 | 2.47E-01 | 6.68E-01 |
| ApoB | oJIA | -5.97E-01 | 4.37E-01 | 1.81E-01 | 7.70E-01 |
| ApoB | sJIA | 9.52E-02 | 3.95E-01 | 8.11E-01 | 9.03E-01 |
| ApoB | pJIA(RF-) | -1.76E-01 | 4.27E-01 | 6.84E-01 | 9.68E-01 |
| ApoA1 | sJIA | 1.08E+00 | 3.33E-01 | 2.47E-03 | 1.59E-02 |
| ApoA1 | pJIA(RF+) | 7.75E-01 | 7.14E-01 | 2.87E-01 | 6.68E-01 |
| ApoA1 | oJIA | 4.19E-01 | 4.30E-01 | 3.38E-01 | 8.00E-01 |
| ApoA1 | pJIA(RF-) | -6.30E-02 | 4.18E-01 | 8.81E-01 | 9.68E-01 |
| ApoB/ApoA1 | sJIA | -5.64E-01 | 3.62E-01 | 1.28E-01 | 2.33E-01 |
| ApoB/ApoA1 | pJIA(RF+) | -1.15E+00 | 5.94E-01 | 6.20E-02 | 4.89E-01 |
| ApoB/ApoA1 | oJIA | -7.59E-01 | 4.11E-01 | 7.40E-02 | 7.70E-01 |
| ApoB/ApoA1 | pJIA(RF-) | -1.45E-01 | 4.29E-01 | 7.37E-01 | 9.68E-01 |
| Total fatty acids | sJIA | 8.85E-01 | 3.73E-01 | 2.32E-02 | 6.33E-02 |
| Total fatty acids | pJIA(RF+) | 2.33E-01 | 7.23E-01 | 7.50E-01 | 9.14E-01 |
| Total fatty acids | oJIA | 1.19E-01 | 4.54E-01 | 7.95E-01 | 9.26E-01 |
| Total fatty acids | pJIA(RF-) | 1.32E-01 | 4.09E-01 | 7.49E-01 | 9.68E-01 |
| Unsaturation | sJIA | 4.43E-01 | 3.76E-01 | 2.47E-01 | 3.98E-01 |
| Unsaturation | oJIA | 1.60E-01 | 4.35E-01 | 7.15E-01 | 8.90E-01 |
| Unsaturation | pJIA(RF+) | -8.90E-02 | 7.29E-01 | 9.04E-01 | 9.67E-01 |
| Unsaturation | pJIA(RF-) | 3.43E-02 | 4.28E-01 | 9.37E-01 | 9.68E-01 |
| Omega-3 | sJIA | 1.07E+00 | 3.43E-01 | 3.62E-03 | 2.14E-02 |
| Omega-3 | pJIA(RF-) | 7.67E-01 | 3.99E-01 | 6.40E-02 | 3.74E-01 |
| Omega-3 | pJIA(RF+) | 5.02E-01 | 7.14E-01 | 4.88E-01 | 7.94E-01 |
| Omega-3 | oJIA | 3.13E-01 | 4.33E-01 | 4.75E-01 | 8.79E-01 |
| Omega-6 | sJIA | 9.96E-01 | 3.68E-01 | 1.03E-02 | 4.12E-02 |
| Omega-6 | oJIA | 2.54E-02 | 4.56E-01 | 9.56E-01 | 9.67E-01 |
| Omega-6 | pJIA(RF+) | -5.86E-02 | 7.16E-01 | 9.35E-01 | 9.67E-01 |
| Omega-6 | pJIA(RF-) | 5.63E-03 | 4.21E-01 | 9.89E-01 | 9.89E-01 |
| PUFA | sJIA | 1.09E+00 | 3.61E-01 | 4.68E-03 | 2.56E-02 |
| PUFA | oJIA | 8.75E-02 | 4.56E-01 | 8.49E-01 | 9.57E-01 |
| PUFA | pJIA(RF+) | 9.31E-02 | 7.22E-01 | 8.98E-01 | 9.67E-01 |
| PUFA | pJIA(RF-) | 1.10E-01 | 4.21E-01 | 7.96E-01 | 9.68E-01 |
| MUFA | sJIA | 7.21E-01 | 3.81E-01 | 6.63E-02 | 1.45E-01 |
| MUFA | pJIA(RF+) | 3.72E-01 | 7.26E-01 | 6.13E-01 | 8.22E-01 |
| MUFA | oJIA | 2.73E-01 | 4.47E-01 | 5.46E-01 | 8.79E-01 |
| MUFA | pJIA(RF-) | 1.13E-01 | 4.03E-01 | 7.80E-01 | 9.68E-01 |
| SFA | sJIA | 8.00E-01 | 3.77E-01 | 4.06E-02 | 9.31E-02 |
| SFA | pJIA(RF+) | 2.10E-01 | 7.20E-01 | 7.72E-01 | 9.14E-01 |
| SFA | oJIA | -2.38E-02 | 4.55E-01 | 9.59E-01 | 9.67E-01 |
| SFA | pJIA(RF-) | 8.80E-02 | 4.09E-01 | 8.31E-01 | 9.68E-01 |
| LA | sJIA | 9.57E-01 | 3.70E-01 | 1.39E-02 | 4.38E-02 |
| LA | oJIA | -1.92E-02 | 4.56E-01 | 9.67E-01 | 9.67E-01 |
| LA | pJIA(RF+) | 7.93E-02 | 7.21E-01 | 9.13E-01 | 9.67E-01 |
| LA | pJIA(RF-) | 1.73E-01 | 4.23E-01 | 6.86E-01 | 9.68E-01 |
| DHA | sJIA | 1.32E+00 | 3.22E-01 | 2.25E-04 | 5.33E-03 |
| DHA | pJIA(RF-) | 8.12E-01 | 4.04E-01 | 5.34E-02 | 3.74E-01 |
| DHA | pJIA(RF+) | 7.38E-01 | 6.96E-01 | 2.98E-01 | 6.68E-01 |
| DHA | oJIA | 7.96E-01 | 4.16E-01 | 6.43E-02 | 7.70E-01 |
| PUFA/MUFA | pJIA(RF+) | -4.66E-01 | 7.27E-01 | 5.27E-01 | 8.04E-01 |
| PUFA/MUFA | sJIA | -2.06E-01 | 3.90E-01 | 6.00E-01 | 8.46E-01 |
| PUFA/MUFA | oJIA | -3.63E-01 | 4.37E-01 | 4.13E-01 | 8.79E-01 |
| PUFA/MUFA | pJIA(RF-) | -6.04E-02 | 4.05E-01 | 8.82E-01 | 9.68E-01 |
| Omega-6/Omega-3 | sJIA | -8.97E-01 | 3.44E-01 | 1.32E-02 | 4.38E-02 |
| Omega-6/Omega-3 | pJIA(RF-) | -9.01E-01 | 3.89E-01 | 2.74E-02 | 3.74E-01 |
| Omega-6/Omega-3 | pJIA(RF+) | -5.34E-01 | 7.01E-01 | 4.53E-01 | 7.94E-01 |
| Omega-6/Omega-3 | oJIA | -3.37E-01 | 4.25E-01 | 4.33E-01 | 8.79E-01 |
| Alanine | sJIA | 7.51E-01 | 3.39E-01 | 3.31E-02 | 7.84E-02 |
| Alanine | pJIA(RF+) | 1.44E+00 | 5.16E-01 | 9.50E-03 | 4.11E-01 |
| Alanine | pJIA(RF-) | 6.39E-01 | 3.88E-01 | 1.09E-01 | 4.88E-01 |
| Alanine | oJIA | 2.21E-01 | 3.79E-01 | 5.63E-01 | 8.79E-01 |
| Glutamine | sJIA | -3.72E-01 | 3.82E-01 | 3.37E-01 | 5.21E-01 |
| Glutamine | pJIA(RF+) | -4.57E-01 | 7.30E-01 | 5.36E-01 | 8.04E-01 |
| Glutamine | oJIA | -3.01E-01 | 4.37E-01 | 4.96E-01 | 8.79E-01 |
| Glutamine | pJIA(RF-) | -4.59E-01 | 4.04E-01 | 2.64E-01 | 9.68E-01 |
| Glycine | pJIA(RF+) | 1.19E+00 | 6.75E-01 | 8.85E-02 | 5.10E-01 |
| Glycine | oJIA | 2.71E-01 | 4.51E-01 | 5.52E-01 | 8.79E-01 |
| Glycine | sJIA | -1.35E-01 | 4.00E-01 | 7.37E-01 | 9.03E-01 |
| Glycine | pJIA(RF-) | -2.17E-01 | 4.21E-01 | 6.09E-01 | 9.68E-01 |
| Histidine | sJIA | -1.23E+00 | 3.38E-01 | 8.31E-04 | 8.43E-03 |
| Histidine | oJIA | -4.24E-01 | 3.79E-01 | 2.71E-01 | 7.70E-01 |
| Histidine | pJIA(RF+) | 5.04E-01 | 7.23E-01 | 4.92E-01 | 7.94E-01 |
| Histidine | pJIA(RF-) | -4.60E-01 | 4.12E-01 | 2.73E-01 | 9.68E-01 |
| Total BCAA | sJIA | 3.82E-01 | 3.48E-01 | 2.79E-01 | 4.41E-01 |
| Total BCAA | pJIA(RF+) | 1.10E+00 | 6.46E-01 | 1.01E-01 | 5.10E-01 |
| Total BCAA | oJIA | 1.68E-01 | 3.72E-01 | 6.54E-01 | 8.84E-01 |
| Total BCAA | pJIA(RF-) | 1.56E-01 | 3.38E-01 | 6.48E-01 | 9.68E-01 |
| Isoleucine | sJIA | 4.85E-01 | 3.27E-01 | 1.46E-01 | 2.60E-01 |
| Isoleucine | pJIA(RF+) | 1.33E+00 | 6.25E-01 | 4.23E-02 | 4.11E-01 |
| Isoleucine | oJIA | 3.12E-01 | 3.72E-01 | 4.08E-01 | 8.79E-01 |
| Isoleucine | pJIA(RF-) | 1.04E-01 | 3.59E-01 | 7.74E-01 | 9.68E-01 |
| Leucine | pJIA(RF+) | 8.01E-01 | 6.61E-01 | 2.36E-01 | 6.68E-01 |
| Leucine | oJIA | 1.65E-01 | 3.71E-01 | 6.60E-01 | 8.84E-01 |
| Leucine | sJIA | 9.84E-02 | 3.33E-01 | 7.69E-01 | 9.03E-01 |
| Leucine | pJIA(RF-) | -4.75E-02 | 3.27E-01 | 8.85E-01 | 9.68E-01 |
| Valine | sJIA | 4.51E-01 | 3.64E-01 | 2.24E-01 | 3.70E-01 |
| Valine | pJIA(RF+) | 1.12E+00 | 6.55E-01 | 9.78E-02 | 5.10E-01 |
| Valine | oJIA | 1.06E-01 | 3.82E-01 | 7.83E-01 | 9.26E-01 |
| Valine | pJIA(RF-) | 2.82E-01 | 3.48E-01 | 4.23E-01 | 9.68E-01 |
| Phenylalanine | pJIA(RF-) | 1.07E+00 | 3.25E-01 | 2.45E-03 | 1.74E-01 |
| Phenylalanine | pJIA(RF+) | 1.32E+00 | 5.84E-01 | 3.14E-02 | 4.11E-01 |
| Phenylalanine | sJIA | 2.65E-01 | 3.13E-01 | 4.03E-01 | 6.06E-01 |
| Phenylalanine | oJIA | 4.52E-01 | 3.81E-01 | 2.44E-01 | 7.70E-01 |
| Tyrosine | sJIA | 9.65E-01 | 3.62E-01 | 1.14E-02 | 4.12E-02 |
| Tyrosine | pJIA(RF-) | 7.11E-01 | 3.68E-01 | 6.24E-02 | 3.74E-01 |
| Tyrosine | pJIA(RF+) | 1.39E+00 | 6.53E-01 | 4.20E-02 | 4.11E-01 |
| Tyrosine | oJIA | -2.20E-01 | 4.08E-01 | 5.92E-01 | 8.79E-01 |
| Pyruvate | sJIA | 7.05E-01 | 3.88E-01 | 7.85E-02 | 1.59E-01 |
| Pyruvate | pJIA(RF+) | 5.75E-01 | 7.17E-01 | 4.30E-01 | 7.85E-01 |
| Pyruvate | oJIA | 5.17E-02 | 4.64E-01 | 9.12E-01 | 9.67E-01 |
| Pyruvate | pJIA(RF-) | 1.86E-01 | 4.35E-01 | 6.73E-01 | 9.68E-01 |
| Citrate | sJIA | -8.71E-01 | 3.72E-01 | 2.50E-02 | 6.34E-02 |
| Citrate | pJIA(RF-) | -7.49E-01 | 4.04E-01 | 7.33E-02 | 3.74E-01 |
| Citrate | oJIA | 1.87E-01 | 4.56E-01 | 6.84E-01 | 8.90E-01 |
| Citrate | pJIA(RF+) | 4.06E-02 | 6.91E-01 | 9.54E-01 | 9.67E-01 |
| Acetate | sJIA | -9.10E-01 | 3.53E-01 | 1.42E-02 | 4.38E-02 |
| Acetate | pJIA(RF-) | -7.09E-01 | 3.30E-01 | 3.92E-02 | 3.74E-01 |
| Acetate | pJIA(RF+) | -9.88E-01 | 4.73E-01 | 4.58E-02 | 4.11E-01 |
| Acetate | oJIA | -7.09E-01 | 3.52E-01 | 5.22E-02 | 7.70E-01 |
| Acetoacetate | sJIA | -8.25E-01 | 3.65E-01 | 2.99E-02 | 7.33E-02 |
| Acetoacetate | pJIA(RF-) | -7.59E-01 | 3.79E-01 | 5.40E-02 | 3.74E-01 |
| Acetoacetate | pJIA(RF+) | -1.18E+00 | 5.51E-01 | 4.04E-02 | 4.11E-01 |
| Acetoacetate | oJIA | 6.48E-02 | 3.95E-01 | 8.71E-01 | 9.66E-01 |
| Acetone | sJIA | -8.54E-01 | 3.62E-01 | 2.41E-02 | 6.33E-02 |
| Acetone | pJIA(RF-) | -8.07E-01 | 3.81E-01 | 4.25E-02 | 3.74E-01 |
| Acetone | pJIA(RF+) | -1.24E+00 | 5.97E-01 | 4.63E-02 | 4.11E-01 |
| Acetone | oJIA | 2.30E-01 | 4.10E-01 | 5.78E-01 | 8.79E-01 |
| Creatinine | pJIA(RF-) | -5.29E-01 | 3.21E-01 | 1.10E-01 | 4.88E-01 |
| Creatinine | pJIA(RF+) | -2.09E-01 | 4.56E-01 | 6.51E-01 | 8.41E-01 |
| Creatinine | sJIA | 7.69E-02 | 3.24E-01 | 8.14E-01 | 9.03E-01 |
| Creatinine | oJIA | -2.62E-02 | 3.80E-01 | 9.45E-01 | 9.67E-01 |
| Albumin | sJIA | -6.68E-01 | 3.71E-01 | 8.04E-02 | 1.59E-01 |
| Albumin | pJIA(RF-) | -7.63E-01 | 4.06E-01 | 6.96E-02 | 3.74E-01 |
| Albumin | pJIA(RF+) | -1.19E+00 | 6.88E-01 | 9.37E-02 | 5.10E-01 |
| Albumin | oJIA | -3.19E-01 | 4.47E-01 | 4.81E-01 | 8.79E-01 |
| Glycoprotein acetyls | sJIA | 1.47E+00 | 3.20E-01 | 5.44E-05 | 3.87E-03 |
| Glycoprotein acetyls | pJIA(RF-) | 8.09E-01 | 3.91E-01 | 4.71E-02 | 3.74E-01 |
| Glycoprotein acetyls | oJIA | 7.13E-01 | 4.41E-01 | 1.15E-01 | 7.70E-01 |
| Glycoprotein acetyls | pJIA(RF+) | 5.28E-01 | 6.61E-01 | 4.31E-01 | 7.85E-01 |

^a^ Adjusted by BH

**Supplementary Data D7.** Estimated differences in 71 metabolomic biomarkers between active JIA subtypes and controls from a linear regression model with JIA subtype status as the exposure and the biomarker as the outcome, adjusting age and sex of participants (n=61).

| Biomarker | Subtypes | estimate | se | P-value | P*_adj_* ^a^ |
| --- | --- | --- | --- | --- | --- |
| Total-C | sJIA | 7.21E-01 | 4.28E-01 | 1.05E-01 | 2.01E-01 |
| Total-C | pJIA(RF+) | -1.07E+00 | 8.35E-01 | 2.14E-01 | 6.91E-01 |
| Total-C | pJIA(RF-) | -1.07E-01 | 4.88E-01 | 8.28E-01 | 9.27E-01 |
| Total-C | oJIA | -1.07E-01 | 5.57E-01 | 8.49E-01 | 9.84E-01 |
| non-HDL-C | sJIA | 2.78E-01 | 4.47E-01 | 5.41E-01 | 6.74E-01 |
| non-HDL-C | pJIA(RF+) | -1.03E+00 | 8.04E-01 | 2.12E-01 | 6.91E-01 |
| non-HDL-C | oJIA | -2.90E-01 | 5.57E-01 | 6.07E-01 | 9.77E-01 |
| non-HDL-C | pJIA(RF-) | 9.95E-03 | 4.88E-01 | 9.84E-01 | 9.84E-01 |
| Remnant-C | sJIA | 2.71E-01 | 4.48E-01 | 5.50E-01 | 6.74E-01 |
| Remnant-C | pJIA(RF+) | -8.90E-01 | 8.13E-01 | 2.85E-01 | 7.49E-01 |
| Remnant-C | pJIA(RF-) | 1.42E-01 | 4.90E-01 | 7.74E-01 | 9.27E-01 |
| Remnant-C | oJIA | -1.37E-01 | 5.59E-01 | 8.09E-01 | 9.84E-01 |
| VLDL-C | pJIA(RF-) | 5.82E-01 | 4.79E-01 | 2.35E-01 | 6.10E-01 |
| VLDL-C | pJIA(RF+) | -8.29E-01 | 8.04E-01 | 3.13E-01 | 7.94E-01 |
| VLDL-C | sJIA | 1.31E-01 | 4.39E-01 | 7.69E-01 | 7.97E-01 |
| VLDL-C | oJIA | -4.10E-02 | 5.54E-01 | 9.42E-01 | 9.84E-01 |
| Clinical LDL-C | pJIA(RF+) | -1.15E+00 | 7.91E-01 | 1.61E-01 | 6.91E-01 |
| Clinical LDL-C | sJIA | 1.29E-01 | 4.50E-01 | 7.77E-01 | 7.97E-01 |
| Clinical LDL-C | pJIA(RF-) | -1.88E-01 | 4.86E-01 | 7.01E-01 | 9.14E-01 |
| Clinical LDL-C | oJIA | -4.48E-01 | 5.52E-01 | 4.25E-01 | 9.77E-01 |
| LDL-C | sJIA | 2.72E-01 | 4.48E-01 | 5.49E-01 | 6.74E-01 |
| LDL-C | pJIA(RF+) | -1.14E+00 | 7.98E-01 | 1.68E-01 | 6.91E-01 |
| LDL-C | pJIA(RF-) | -1.03E-01 | 4.85E-01 | 8.33E-01 | 9.27E-01 |
| LDL-C | oJIA | -4.25E-01 | 5.53E-01 | 4.50E-01 | 9.77E-01 |
| HDL-C | pJIA(RF-) | -3.66E-01 | 4.85E-01 | 4.56E-01 | 7.90E-01 |
| HDL-C | pJIA(RF+) | -5.28E-01 | 8.80E-01 | 5.54E-01 | 8.12E-01 |
| HDL-C | oJIA | 3.56E-01 | 5.30E-01 | 5.08E-01 | 9.77E-01 |
| Total triglycerides | sJIA | 4.65E-01 | 4.31E-01 | 2.91E-01 | 4.53E-01 |
| Total triglycerides | pJIA(RF-) | 7.47E-01 | 4.31E-01 | 9.45E-02 | 5.05E-01 |
| Total triglycerides | pJIA(RF+) | 5.21E-01 | 8.91E-01 | 5.65E-01 | 8.12E-01 |
| Total triglycerides | oJIA | 3.73E-01 | 5.54E-01 | 5.07E-01 | 9.77E-01 |
| VLDL-TG | pJIA(RF-) | 5.98E-01 | 4.34E-01 | 1.80E-01 | 5.33E-01 |
| VLDL-TG | sJIA | 2.37E-01 | 4.43E-01 | 5.97E-01 | 6.95E-01 |
| VLDL-TG | pJIA(RF+) | 2.88E-01 | 8.87E-01 | 7.48E-01 | 8.71E-01 |
| VLDL-TG | oJIA | 1.05E-01 | 5.59E-01 | 8.53E-01 | 9.84E-01 |
| LDL-TG | pJIA(RF-) | 1.26E+00 | 4.16E-01 | 5.52E-03 | 1.41E-01 |
| LDL-TG | pJIA(RF+) | 7.24E-01 | 8.96E-01 | 4.28E-01 | 8.12E-01 |
| LDL-TG | oJIA | 1.09E+00 | 5.11E-01 | 4.35E-02 | 9.77E-01 |
| HDL-TG | sJIA | 7.04E-01 | 4.08E-01 | 9.70E-02 | 1.99E-01 |
| HDL-TG | pJIA(RF-) | 9.60E-01 | 4.47E-01 | 4.11E-02 | 3.65E-01 |
| HDL-TG | pJIA(RF+) | 1.29E+00 | 8.66E-01 | 1.51E-01 | 6.91E-01 |
| HDL-TG | oJIA | 9.21E-01 | 5.24E-01 | 9.19E-02 | 9.77E-01 |
| Total-PL | pJIA(RF+) | -6.56E-01 | 8.75E-01 | 4.61E-01 | 8.12E-01 |
| Total-PL | pJIA(RF-) | 3.42E-02 | 4.85E-01 | 9.44E-01 | 9.65E-01 |
| Total-PL | oJIA | 3.50E-01 | 5.44E-01 | 5.26E-01 | 9.77E-01 |
| VLDL-PL | pJIA(RF-) | 6.82E-01 | 4.64E-01 | 1.53E-01 | 5.05E-01 |
| VLDL-PL | sJIA | 1.69E-01 | 4.41E-01 | 7.04E-01 | 7.58E-01 |
| VLDL-PL | pJIA(RF+) | -4.23E-01 | 8.37E-01 | 6.18E-01 | 8.12E-01 |
| VLDL-PL | oJIA | 2.20E-02 | 5.55E-01 | 9.69E-01 | 9.84E-01 |
| LDL-PL | pJIA(RF+) | -1.03E+00 | 7.95E-01 | 2.06E-01 | 6.91E-01 |
| LDL-PL | sJIA | 1.94E-01 | 4.48E-01 | 6.69E-01 | 7.31E-01 |
| LDL-PL | pJIA(RF-) | -1.02E-01 | 4.87E-01 | 8.36E-01 | 9.27E-01 |
| LDL-PL | oJIA | -4.49E-01 | 5.52E-01 | 4.24E-01 | 9.77E-01 |
| HDL-PL | pJIA(RF-) | -1.98E-01 | 4.83E-01 | 6.86E-01 | 9.14E-01 |
| HDL-PL | pJIA(RF+) | -6.43E-02 | 8.79E-01 | 9.42E-01 | 9.68E-01 |
| HDL-PL | oJIA | 5.96E-01 | 4.99E-01 | 2.45E-01 | 9.77E-01 |
| Total-CE | sJIA | 7.31E-01 | 4.28E-01 | 1.00E-01 | 1.99E-01 |
| Total-CE | pJIA(RF+) | -1.09E+00 | 8.36E-01 | 2.07E-01 | 6.91E-01 |
| Total-CE | pJIA(RF-) | -1.57E-01 | 4.88E-01 | 7.50E-01 | 9.27E-01 |
| Total-CE | oJIA | -1.37E-01 | 5.56E-01 | 8.07E-01 | 9.84E-01 |
| VLDL-CE | pJIA(RF-) | 5.45E-01 | 4.83E-01 | 2.69E-01 | 6.10E-01 |
| VLDL-CE | pJIA(RF+) | -9.30E-01 | 8.03E-01 | 2.59E-01 | 7.35E-01 |
| VLDL-CE | sJIA | 1.34E-01 | 4.39E-01 | 7.62E-01 | 7.97E-01 |
| VLDL-CE | oJIA | -6.22E-02 | 5.55E-01 | 9.12E-01 | 9.84E-01 |
| LDL-CE | sJIA | 2.92E-01 | 4.47E-01 | 5.19E-01 | 6.74E-01 |
| LDL-CE | pJIA(RF+) | -1.10E+00 | 7.98E-01 | 1.80E-01 | 6.91E-01 |
| LDL-CE | pJIA(RF-) | -4.42E-02 | 4.84E-01 | 9.28E-01 | 9.65E-01 |
| LDL-CE | oJIA | -3.91E-01 | 5.54E-01 | 4.87E-01 | 9.77E-01 |
| HDL-CE | pJIA(RF-) | -4.47E-01 | 4.82E-01 | 3.61E-01 | 6.82E-01 |
| HDL-CE | pJIA(RF+) | -6.04E-01 | 8.81E-01 | 5.00E-01 | 8.12E-01 |
| HDL-CE | oJIA | 3.04E-01 | 5.32E-01 | 5.73E-01 | 9.77E-01 |
| Total-FC | sJIA | 6.82E-01 | 4.30E-01 | 1.25E-01 | 2.17E-01 |
| Total-FC | pJIA(RF+) | -1.01E+00 | 8.35E-01 | 2.39E-01 | 7.08E-01 |
| Total-FC | pJIA(RF-) | 2.98E-02 | 4.90E-01 | 9.52E-01 | 9.65E-01 |
| Total-FC | oJIA | -2.94E-02 | 5.59E-01 | 9.59E-01 | 9.84E-01 |
| VLDL-FC | pJIA(RF-) | 6.07E-01 | 4.70E-01 | 2.08E-01 | 5.68E-01 |
| VLDL-FC | sJIA | 1.21E-01 | 4.40E-01 | 7.86E-01 | 7.97E-01 |
| VLDL-FC | pJIA(RF+) | -6.58E-01 | 8.11E-01 | 4.26E-01 | 8.12E-01 |
| VLDL-FC | oJIA | -1.77E-02 | 5.54E-01 | 9.75E-01 | 9.84E-01 |
| LDL-FC | pJIA(RF+) | -1.20E+00 | 8.01E-01 | 1.47E-01 | 6.91E-01 |
| LDL-FC | sJIA | 2.04E-01 | 4.49E-01 | 6.55E-01 | 7.31E-01 |
| LDL-FC | pJIA(RF-) | -2.48E-01 | 4.85E-01 | 6.14E-01 | 9.14E-01 |
| LDL-FC | oJIA | -5.07E-01 | 5.50E-01 | 3.65E-01 | 9.77E-01 |
| HDL-FC | pJIA(RF+) | -2.55E-01 | 8.77E-01 | 7.73E-01 | 8.79E-01 |
| HDL-FC | pJIA(RF-) | -6.76E-02 | 4.90E-01 | 8.91E-01 | 9.65E-01 |
| HDL-FC | oJIA | 5.41E-01 | 5.22E-01 | 3.10E-01 | 9.77E-01 |
| Total-L | sJIA | 8.20E-01 | 4.09E-01 | 5.57E-02 | 1.24E-01 |
| Total-L | pJIA(RF+) | -7.47E-01 | 8.56E-01 | 3.92E-01 | 8.12E-01 |
| Total-L | pJIA(RF-) | 1.27E-01 | 4.80E-01 | 7.94E-01 | 9.27E-01 |
| Total-L | oJIA | 1.87E-01 | 5.56E-01 | 7.40E-01 | 9.84E-01 |
| VLDL-L | pJIA(RF-) | 6.55E-01 | 4.50E-01 | 1.57E-01 | 5.05E-01 |
| VLDL-L | sJIA | 1.92E-01 | 4.40E-01 | 6.67E-01 | 7.31E-01 |
| VLDL-L | pJIA(RF+) | -2.01E-01 | 8.55E-01 | 8.16E-01 | 8.92E-01 |
| VLDL-L | oJIA | 5.02E-02 | 5.57E-01 | 9.29E-01 | 9.84E-01 |
| LDL-L | sJIA | 3.24E-01 | 4.47E-01 | 4.74E-01 | 6.48E-01 |
| LDL-L | pJIA(RF+) | -1.05E+00 | 8.07E-01 | 2.08E-01 | 6.91E-01 |
| LDL-L | pJIA(RF-) | -4.50E-02 | 4.86E-01 | 9.27E-01 | 9.65E-01 |
| LDL-L | oJIA | -3.84E-01 | 5.54E-01 | 4.95E-01 | 9.77E-01 |
| HDL-L | pJIA(RF+) | -2.19E-01 | 8.82E-01 | 8.06E-01 | 8.92E-01 |
| HDL-L | pJIA(RF-) | -2.23E-01 | 4.84E-01 | 6.48E-01 | 9.14E-01 |
| HDL-L | oJIA | 5.43E-01 | 5.12E-01 | 3.00E-01 | 9.77E-01 |
| Total-P | sJIA | 9.18E-01 | 3.86E-01 | 2.54E-02 | 6.92E-02 |
| Total-P | pJIA(RF-) | -4.21E-01 | 4.70E-01 | 3.79E-01 | 6.89E-01 |
| Total-P | pJIA(RF+) | -8.06E-01 | 8.67E-01 | 3.62E-01 | 8.12E-01 |
| Total-P | oJIA | 7.40E-02 | 5.35E-01 | 8.91E-01 | 9.84E-01 |
| VLDL-P | pJIA(RF-) | 8.71E-01 | 4.57E-01 | 6.74E-02 | 5.05E-01 |
| VLDL-P | sJIA | 3.89E-01 | 4.37E-01 | 3.81E-01 | 5.31E-01 |
| VLDL-P | pJIA(RF+) | -4.59E-01 | 8.42E-01 | 5.91E-01 | 8.12E-01 |
| VLDL-P | oJIA | 9.82E-02 | 5.56E-01 | 8.61E-01 | 9.84E-01 |
| LDL-P | sJIA | 2.61E-01 | 4.35E-01 | 5.55E-01 | 6.74E-01 |
| LDL-P | pJIA(RF+) | -1.15E+00 | 8.00E-01 | 1.63E-01 | 6.91E-01 |
| LDL-P | pJIA(RF-) | 1.74E-01 | 4.91E-01 | 7.26E-01 | 9.20E-01 |
| LDL-P | oJIA | -2.80E-01 | 5.52E-01 | 6.17E-01 | 9.77E-01 |
| HDL-P | sJIA | 9.12E-01 | 3.78E-01 | 2.33E-02 | 6.71E-02 |
| HDL-P | pJIA(RF-) | -4.80E-01 | 4.66E-01 | 3.13E-01 | 6.73E-01 |
| HDL-P | pJIA(RF+) | -7.00E-01 | 8.77E-01 | 4.33E-01 | 8.12E-01 |
| HDL-P | oJIA | 1.10E-01 | 5.29E-01 | 8.37E-01 | 9.84E-01 |
| VLDL particle size | sJIA | -4.03E-01 | 4.37E-01 | 3.66E-01 | 5.20E-01 |
| VLDL particle size | pJIA(RF-) | 3.87E-02 | 4.46E-01 | 9.32E-01 | 9.65E-01 |
| VLDL particle size | pJIA(RF+) | -6.34E-02 | 8.68E-01 | 9.42E-01 | 9.68E-01 |
| VLDL particle size | oJIA | -2.47E-01 | 5.54E-01 | 6.59E-01 | 9.77E-01 |
| LDL particle size | sJIA | 4.42E-01 | 4.04E-01 | 2.84E-01 | 4.53E-01 |
| LDL particle size | pJIA(RF-) | -7.38E-01 | 4.71E-01 | 1.29E-01 | 5.05E-01 |
| LDL particle size | pJIA(RF+) | -5.98E-01 | 8.72E-01 | 5.00E-01 | 8.12E-01 |
| LDL particle size | oJIA | -3.69E-01 | 5.43E-01 | 5.04E-01 | 9.77E-01 |
| HDL particle size | pJIA(RF+) | 2.83E-01 | 7.38E-01 | 7.04E-01 | 8.48E-01 |
| HDL particle size | pJIA(RF-) | 2.80E-01 | 4.88E-01 | 5.71E-01 | 9.02E-01 |
| HDL particle size | oJIA | 8.84E-01 | 5.24E-01 | 1.05E-01 | 9.77E-01 |
| Phosphoglycerides | pJIA(RF+) | -3.44E-01 | 8.91E-01 | 7.03E-01 | 8.48E-01 |
| Phosphoglycerides | pJIA(RF-) | 1.86E-01 | 4.82E-01 | 7.03E-01 | 9.14E-01 |
| Phosphoglycerides | oJIA | 5.09E-01 | 5.31E-01 | 3.47E-01 | 9.77E-01 |
| TG/PG | pJIA(RF-) | 6.84E-01 | 4.54E-01 | 1.44E-01 | 5.05E-01 |
| TG/PG | pJIA(RF+) | 6.70E-01 | 8.92E-01 | 4.60E-01 | 8.12E-01 |
| TG/PG | sJIA | 3.25E-02 | 4.46E-01 | 9.42E-01 | 9.42E-01 |
| TG/PG | oJIA | 1.50E-01 | 5.54E-01 | 7.88E-01 | 9.84E-01 |
| Total cholines | pJIA(RF+) | -4.63E-01 | 8.91E-01 | 6.09E-01 | 8.12E-01 |
| Total cholines | pJIA(RF-) | 1.46E-01 | 4.85E-01 | 7.65E-01 | 9.27E-01 |
| Total cholines | oJIA | 4.41E-01 | 5.37E-01 | 4.20E-01 | 9.77E-01 |
| Phosphatidylcholines | sJIA | 9.59E-01 | 3.88E-01 | 2.05E-02 | 6.33E-02 |
| Phosphatidylcholines | pJIA(RF+) | -3.23E-01 | 8.89E-01 | 7.19E-01 | 8.51E-01 |
| Phosphatidylcholines | pJIA(RF-) | 2.31E-01 | 4.82E-01 | 6.35E-01 | 9.14E-01 |
| Phosphatidylcholines | oJIA | 4.49E-01 | 5.34E-01 | 4.09E-01 | 9.77E-01 |
| Sphingomyelins | pJIA(RF+) | -4.80E-01 | 9.01E-01 | 5.99E-01 | 8.12E-01 |
| Sphingomyelins | pJIA(RF-) | 3.00E-01 | 4.91E-01 | 5.46E-01 | 9.02E-01 |
| Sphingomyelins | oJIA | 2.45E-01 | 5.52E-01 | 6.61E-01 | 9.77E-01 |
| ApoB | sJIA | 2.54E-01 | 4.40E-01 | 5.70E-01 | 6.74E-01 |
| ApoB | pJIA(RF+) | -1.03E+00 | 8.06E-01 | 2.14E-01 | 6.91E-01 |
| ApoB | pJIA(RF-) | 1.93E-01 | 4.91E-01 | 6.98E-01 | 9.14E-01 |
| ApoB | oJIA | -2.75E-01 | 5.54E-01 | 6.25E-01 | 9.77E-01 |
| ApoA1 | pJIA(RF-) | -3.35E-01 | 4.77E-01 | 4.88E-01 | 8.25E-01 |
| ApoA1 | pJIA(RF+) | -3.46E-01 | 8.93E-01 | 7.02E-01 | 8.48E-01 |
| ApoA1 | oJIA | 4.11E-01 | 5.18E-01 | 4.35E-01 | 9.77E-01 |
| ApoB/ApoA1 | sJIA | -4.26E-01 | 4.01E-01 | 2.98E-01 | 4.53E-01 |
| ApoB/ApoA1 | pJIA(RF-) | 4.57E-01 | 4.84E-01 | 3.54E-01 | 6.82E-01 |
| ApoB/ApoA1 | pJIA(RF+) | -8.77E-01 | 7.95E-01 | 2.81E-01 | 7.49E-01 |
| ApoB/ApoA1 | oJIA | -4.80E-01 | 5.27E-01 | 3.72E-01 | 9.77E-01 |
| Total fatty acids | sJIA | 9.59E-01 | 3.85E-01 | 1.96E-02 | 6.33E-02 |
| Total fatty acids | pJIA(RF-) | 5.24E-01 | 4.64E-01 | 2.68E-01 | 6.10E-01 |
| Total fatty acids | pJIA(RF+) | -5.90E-02 | 8.89E-01 | 9.48E-01 | 9.68E-01 |
| Total fatty acids | oJIA | 6.33E-01 | 5.42E-01 | 2.54E-01 | 9.77E-01 |
| Unsaturation | sJIA | 3.57E-01 | 3.64E-01 | 3.37E-01 | 4.88E-01 |
| Unsaturation | pJIA(RF-) | -5.53E-01 | 4.86E-01 | 2.65E-01 | 6.10E-01 |
| Unsaturation | pJIA(RF+) | -1.63E+00 | 7.94E-01 | 5.19E-02 | 6.91E-01 |
| Unsaturation | oJIA | 2.45E-02 | 5.16E-01 | 9.63E-01 | 9.84E-01 |
| Omega-3 | sJIA | 9.05E-01 | 3.91E-01 | 2.91E-02 | 7.13E-02 |
| Omega-3 | pJIA(RF-) | 6.95E-01 | 4.69E-01 | 1.50E-01 | 5.05E-01 |
| Omega-3 | pJIA(RF+) | -5.69E-01 | 8.73E-01 | 5.21E-01 | 8.12E-01 |
| Omega-3 | oJIA | 7.26E-01 | 5.10E-01 | 1.67E-01 | 9.77E-01 |
| Omega-6 | pJIA(RF+) | -5.50E-01 | 8.79E-01 | 5.38E-01 | 8.12E-01 |
| Omega-6 | pJIA(RF-) | 1.21E-01 | 4.88E-01 | 8.06E-01 | 9.27E-01 |
| Omega-6 | oJIA | 2.97E-01 | 5.54E-01 | 5.97E-01 | 9.77E-01 |
| PUFA | pJIA(RF+) | -6.05E-01 | 8.85E-01 | 5.01E-01 | 8.12E-01 |
| PUFA | pJIA(RF-) | 2.05E-01 | 4.87E-01 | 6.77E-01 | 9.14E-01 |
| PUFA | oJIA | 4.22E-01 | 5.48E-01 | 4.48E-01 | 9.77E-01 |
| MUFA | sJIA | 8.15E-01 | 4.02E-01 | 5.36E-02 | 1.23E-01 |
| MUFA | pJIA(RF-) | 7.11E-01 | 4.46E-01 | 1.23E-01 | 5.05E-01 |
| MUFA | pJIA(RF+) | 5.03E-01 | 8.85E-01 | 5.75E-01 | 8.12E-01 |
| MUFA | oJIA | 7.12E-01 | 5.34E-01 | 1.95E-01 | 9.77E-01 |
| SFA | sJIA | 8.56E-01 | 3.69E-01 | 2.89E-02 | 7.13E-02 |
| SFA | pJIA(RF-) | 5.40E-01 | 4.62E-01 | 2.52E-01 | 6.10E-01 |
| SFA | pJIA(RF+) | -5.09E-02 | 8.77E-01 | 9.54E-01 | 9.68E-01 |
| SFA | oJIA | 6.53E-01 | 5.39E-01 | 2.37E-01 | 9.77E-01 |
| LA | pJIA(RF+) | -2.52E-01 | 8.92E-01 | 7.80E-01 | 8.79E-01 |
| LA | pJIA(RF-) | 2.49E-01 | 4.90E-01 | 6.15E-01 | 9.14E-01 |
| LA | oJIA | 2.91E-01 | 5.54E-01 | 6.04E-01 | 9.77E-01 |
| DHA | pJIA(RF-) | 7.01E-01 | 4.77E-01 | 1.53E-01 | 5.05E-01 |
| DHA | pJIA(RF+) | -4.54E-01 | 8.44E-01 | 5.96E-01 | 8.12E-01 |
| DHA | oJIA | 6.83E-01 | 5.03E-01 | 1.87E-01 | 9.77E-01 |
| PUFA/MUFA | pJIA(RF-) | -7.83E-01 | 4.45E-01 | 8.98E-02 | 5.05E-01 |
| PUFA/MUFA | sJIA | -2.82E-01 | 4.13E-01 | 5.02E-01 | 6.72E-01 |
| PUFA/MUFA | pJIA(RF+) | -1.35E+00 | 8.39E-01 | 1.22E-01 | 6.91E-01 |
| PUFA/MUFA | oJIA | -7.19E-01 | 5.17E-01 | 1.77E-01 | 9.77E-01 |
| Omega-6/Omega-3 | sJIA | -6.76E-01 | 3.97E-01 | 1.01E-01 | 1.99E-01 |
| Omega-6/Omega-3 | pJIA(RF-) | -7.81E-01 | 4.62E-01 | 1.02E-01 | 5.05E-01 |
| Omega-6/Omega-3 | pJIA(RF+) | 3.72E-01 | 8.51E-01 | 6.66E-01 | 8.48E-01 |
| Omega-6/Omega-3 | oJIA | -7.03E-01 | 5.08E-01 | 1.79E-01 | 9.77E-01 |
| Alanine | sJIA | 8.61E-01 | 3.69E-01 | 2.79E-02 | 7.13E-02 |
| Alanine | pJIA(RF+) | 1.52E+00 | 6.39E-01 | 2.65E-02 | 6.19E-01 |
| Alanine | pJIA(RF-) | 4.07E-01 | 4.41E-01 | 3.65E-01 | 6.82E-01 |
| Alanine | oJIA | 2.94E-01 | 5.03E-01 | 5.65E-01 | 9.77E-01 |
| Glutamine | sJIA | -4.41E-01 | 4.45E-01 | 3.31E-01 | 4.88E-01 |
| Glutamine | pJIA(RF-) | -8.01E-01 | 4.43E-01 | 8.22E-02 | 5.05E-01 |
| Glutamine | pJIA(RF+) | -6.02E-01 | 9.06E-01 | 5.13E-01 | 8.12E-01 |
| Glutamine | oJIA | -2.65E-01 | 5.46E-01 | 6.32E-01 | 9.77E-01 |
| Glycine | sJIA | -6.92E-01 | 4.28E-01 | 1.19E-01 | 2.10E-01 |
| Glycine | pJIA(RF-) | -7.26E-01 | 4.69E-01 | 1.33E-01 | 5.05E-01 |
| Glycine | pJIA(RF+) | 1.25E+00 | 8.19E-01 | 1.40E-01 | 6.91E-01 |
| Glycine | oJIA | 7.19E-01 | 5.24E-01 | 1.83E-01 | 9.77E-01 |
| Histidine | pJIA(RF-) | -7.28E-01 | 4.67E-01 | 1.30E-01 | 5.05E-01 |
| Histidine | pJIA(RF+) | -4.77E-01 | 8.85E-01 | 5.95E-01 | 8.12E-01 |
| Histidine | oJIA | -3.44E-01 | 5.25E-01 | 5.19E-01 | 9.77E-01 |
| Total BCAA | sJIA | 5.13E-01 | 3.89E-01 | 2.00E-01 | 3.30E-01 |
| Total BCAA | pJIA(RF-) | 3.86E-01 | 4.02E-01 | 3.46E-01 | 6.82E-01 |
| Total BCAA | pJIA(RF+) | -1.77E-02 | 7.63E-01 | 9.82E-01 | 9.82E-01 |
| Total BCAA | oJIA | 4.49E-02 | 4.87E-01 | 9.27E-01 | 9.84E-01 |
| Isoleucine | sJIA | 6.04E-01 | 3.71E-01 | 1.16E-01 | 2.10E-01 |
| Isoleucine | pJIA(RF-) | 3.74E-01 | 4.28E-01 | 3.90E-01 | 6.92E-01 |
| Isoleucine | pJIA(RF+) | 3.15E-01 | 7.50E-01 | 6.79E-01 | 8.48E-01 |
| Isoleucine | oJIA | 4.20E-01 | 4.88E-01 | 3.98E-01 | 9.77E-01 |
| Leucine | sJIA | 2.16E-01 | 3.72E-01 | 5.67E-01 | 6.74E-01 |
| Leucine | pJIA(RF+) | -4.38E-01 | 7.54E-01 | 5.67E-01 | 8.12E-01 |
| Leucine | pJIA(RF-) | 2.31E-01 | 3.90E-01 | 5.60E-01 | 9.02E-01 |
| Leucine | oJIA | -1.38E-01 | 4.77E-01 | 7.76E-01 | 9.84E-01 |
| Valine | sJIA | 5.39E-01 | 4.05E-01 | 1.95E-01 | 3.30E-01 |
| Valine | pJIA(RF-) | 4.59E-01 | 4.12E-01 | 2.75E-01 | 6.10E-01 |
| Valine | pJIA(RF+) | 1.13E-01 | 7.97E-01 | 8.88E-01 | 9.55E-01 |
| Valine | oJIA | 4.41E-02 | 4.97E-01 | 9.30E-01 | 9.84E-01 |
| Phenylalanine | pJIA(RF-) | 1.16E+00 | 3.87E-01 | 5.97E-03 | 1.41E-01 |
| Phenylalanine | sJIA | 3.84E-01 | 3.62E-01 | 3.00E-01 | 4.53E-01 |
| Phenylalanine | pJIA(RF+) | 4.23E-01 | 7.03E-01 | 5.53E-01 | 8.12E-01 |
| Phenylalanine | oJIA | 4.78E-01 | 4.87E-01 | 3.36E-01 | 9.77E-01 |
| Tyrosine | pJIA(RF-) | 1.03E+00 | 4.02E-01 | 1.68E-02 | 1.98E-01 |
| Tyrosine | pJIA(RF+) | 8.24E-01 | 8.60E-01 | 3.48E-01 | 8.12E-01 |
| Tyrosine | oJIA | -5.42E-01 | 4.96E-01 | 2.86E-01 | 9.77E-01 |
| Pyruvate | sJIA | 9.38E-01 | 3.66E-01 | 1.75E-02 | 6.11E-02 |
| Pyruvate | pJIA(RF+) | 1.14E+00 | 8.45E-01 | 1.91E-01 | 6.91E-01 |
| Pyruvate | pJIA(RF-) | 1.88E-01 | 4.95E-01 | 7.08E-01 | 9.14E-01 |
| Pyruvate | oJIA | -2.68E-01 | 5.69E-01 | 6.43E-01 | 9.77E-01 |
| Citrate | sJIA | -9.70E-01 | 3.83E-01 | 1.81E-02 | 6.11E-02 |
| Citrate | pJIA(RF-) | -1.23E+00 | 4.31E-01 | 8.26E-03 | 1.47E-01 |
| Citrate | pJIA(RF+) | -9.92E-01 | 8.09E-01 | 2.33E-01 | 7.08E-01 |
| Citrate | oJIA | 1.70E-01 | 5.52E-01 | 7.61E-01 | 9.84E-01 |
| Acetate | sJIA | -8.39E-01 | 3.48E-01 | 2.36E-02 | 6.71E-02 |
| Acetate | pJIA(RF-) | -9.31E-01 | 3.86E-01 | 2.31E-02 | 2.34E-01 |
| Acetate | pJIA(RF+) | -8.06E-01 | 5.49E-01 | 1.56E-01 | 6.91E-01 |
| Acetate | oJIA | -7.98E-01 | 4.34E-01 | 7.83E-02 | 9.77E-01 |
| Acetoacetate | sJIA | -7.67E-01 | 4.15E-01 | 7.65E-02 | 1.65E-01 |
| Acetoacetate | pJIA(RF-) | -5.89E-01 | 4.47E-01 | 1.99E-01 | 5.65E-01 |
| Acetoacetate | pJIA(RF+) | -1.65E+00 | 6.28E-01 | 1.50E-02 | 6.19E-01 |
| Acetoacetate | oJIA | -6.96E-02 | 5.00E-01 | 8.90E-01 | 9.84E-01 |
| Acetone | sJIA | -8.64E-01 | 4.07E-01 | 4.35E-02 | 1.03E-01 |
| Acetone | pJIA(RF-) | -6.50E-01 | 4.54E-01 | 1.64E-01 | 5.05E-01 |
| Acetone | pJIA(RF+) | -1.61E+00 | 7.19E-01 | 3.49E-02 | 6.19E-01 |
| Acetone | oJIA | -8.96E-02 | 5.15E-01 | 8.63E-01 | 9.84E-01 |
| Creatinine | pJIA(RF-) | -3.74E-01 | 3.89E-01 | 3.45E-01 | 6.82E-01 |
| Creatinine | sJIA | 1.65E-01 | 3.68E-01 | 6.58E-01 | 7.31E-01 |
| Creatinine | pJIA(RF+) | -3.57E-01 | 5.94E-01 | 5.54E-01 | 8.12E-01 |
| Creatinine | oJIA | -9.74E-03 | 4.80E-01 | 9.84E-01 | 9.84E-01 |
| Albumin | pJIA(RF-) | -1.17E+00 | 4.37E-01 | 1.24E-02 | 1.76E-01 |
| Albumin | sJIA | -6.68E-01 | 4.08E-01 | 1.14E-01 | 2.10E-01 |
| Albumin | pJIA(RF+) | -1.92E+00 | 7.95E-01 | 2.42E-02 | 6.19E-01 |
| Albumin | oJIA | -3.51E-01 | 5.45E-01 | 5.26E-01 | 9.77E-01 |
| Glycoprotein acetyls | pJIA(RF-) | 1.48E+00 | 3.99E-01 | 9.51E-04 | 6.75E-02 |
| Glycoprotein acetyls | pJIA(RF+) | 1.48E+00 | 7.76E-01 | 6.83E-02 | 6.91E-01 |
| Glycoprotein acetyls | oJIA | 7.45E-01 | 5.31E-01 | 1.74E-01 | 9.77E-01 |
| HDL-C | sJIA | 1.21E+00 | 3.43E-01 | 1.61E-03 | 1.43E-02 |
| LDL-TG | sJIA | 1.21E+00 | 3.73E-01 | 3.33E-03 | 2.27E-02 |
| Total-PL | sJIA | 1.04E+00 | 3.82E-01 | 1.19E-02 | 4.44E-02 |
| HDL-PL | sJIA | 1.19E+00 | 3.19E-01 | 9.92E-04 | 1.01E-02 |
| HDL-CE | sJIA | 1.15E+00 | 3.54E-01 | 3.29E-03 | 2.27E-02 |
| HDL-FC | sJIA | 1.38E+00 | 3.14E-01 | 1.83E-04 | 4.33E-03 |
| HDL-L | sJIA | 1.23E+00 | 3.23E-01 | 7.88E-04 | 1.01E-02 |
| HDL particle size | sJIA | 1.37E+00 | 3.30E-01 | 3.48E-04 | 6.18E-03 |
| Phosphoglycerides | sJIA | 1.04E+00 | 3.74E-01 | 1.01E-02 | 4.21E-02 |
| Total cholines | sJIA | 1.09E+00 | 3.74E-01 | 7.44E-03 | 3.52E-02 |
| Sphingomyelins | sJIA | 1.35E+00 | 3.59E-01 | 9.18E-04 | 1.01E-02 |
| ApoA1 | sJIA | 1.09E+00 | 3.43E-01 | 3.86E-03 | 2.28E-02 |
| Omega-6 | sJIA | 1.11E+00 | 3.95E-01 | 9.71E-03 | 4.21E-02 |
| PUFA | sJIA | 1.17E+00 | 3.89E-01 | 6.00E-03 | 3.04E-02 |
| LA | sJIA | 1.08E+00 | 3.99E-01 | 1.18E-02 | 4.44E-02 |
| DHA | sJIA | 1.15E+00 | 3.69E-01 | 4.69E-03 | 2.56E-02 |
| Histidine | sJIA | -1.41E+00 | 3.17E-01 | 1.61E-04 | 4.33E-03 |
| Tyrosine | sJIA | 1.19E+00 | 3.71E-01 | 3.52E-03 | 2.27E-02 |
| Glycoprotein acetyls | sJIA | 1.61E+00 | 3.13E-01 | 2.46E-05 | 1.75E-03 |

^a^ Adjusted by BH

**Supplementary Data D8.** Estimated differences in 71 metabolomic biomarkers between inactive JIA subtypes and controls from a linear regression model with JIA subtype status as the exposure and the biomarker as the outcome, adjusting age and sex of participants (n=31).

| Biomarker | Subtypes | estimate | se | P-value | P*_adj_* ^a^ |
| --- | --- | --- | --- | --- | --- |
| Total-C | oJIA | -1.08E+00 | 7.91E-01 | 1.90E-01 | 4.72E-01 |
| Total-C | sJIA | 6.61E-01 | 7.63E-01 | 3.96E-01 | 6.69E-01 |
| Total-C | pJIA(RF-) | -3.14E-01 | 1.29E+00 | 8.12E-01 | 9.45E-01 |
| Total-C | pJIA(RF+) | 1.19E-01 | 1.17E+00 | 9.20E-01 | 9.92E-01 |
| non-HDL-C | oJIA | -1.12E+00 | 7.80E-01 | 1.69E-01 | 4.69E-01 |
| non-HDL-C | pJIA(RF-) | -1.01E+00 | 1.27E+00 | 4.40E-01 | 7.52E-01 |
| non-HDL-C | sJIA | 3.32E-01 | 7.77E-01 | 6.73E-01 | 8.85E-01 |
| non-HDL-C | pJIA(RF+) | -3.61E-01 | 1.16E+00 | 7.61E-01 | 9.92E-01 |
| Remnant-C | oJIA | -1.32E+00 | 7.50E-01 | 9.68E-02 | 4.59E-01 |
| Remnant-C | pJIA(RF-) | -1.22E+00 | 1.26E+00 | 3.48E-01 | 7.26E-01 |
| Remnant-C | sJIA | 5.09E-01 | 7.73E-01 | 5.17E-01 | 7.65E-01 |
| Remnant-C | pJIA(RF+) | -2.17E-01 | 1.16E+00 | 8.54E-01 | 9.92E-01 |
| VLDL-C | oJIA | -1.45E+00 | 7.11E-01 | 5.75E-02 | 4.00E-01 |
| VLDL-C | pJIA(RF-) | -1.89E+00 | 1.20E+00 | 1.36E-01 | 6.09E-01 |
| VLDL-C | sJIA | 2.62E-01 | 7.76E-01 | 7.39E-01 | 8.89E-01 |
| VLDL-C | pJIA(RF+) | -3.94E-01 | 1.13E+00 | 7.32E-01 | 9.92E-01 |
| Clinical LDL-C | oJIA | -1.02E+00 | 7.96E-01 | 2.18E-01 | 4.72E-01 |
| Clinical LDL-C | pJIA(RF-) | -9.35E-01 | 1.26E+00 | 4.71E-01 | 7.52E-01 |
| Clinical LDL-C | sJIA | 8.52E-02 | 7.77E-01 | 9.14E-01 | 9.30E-01 |
| Clinical LDL-C | pJIA(RF+) | -6.46E-01 | 1.16E+00 | 5.85E-01 | 9.92E-01 |
| LDL-C | oJIA | -9.26E-01 | 8.04E-01 | 2.65E-01 | 4.72E-01 |
| LDL-C | pJIA(RF-) | -7.97E-01 | 1.27E+00 | 5.41E-01 | 7.68E-01 |
| LDL-C | sJIA | 1.69E-01 | 7.78E-01 | 8.30E-01 | 9.07E-01 |
| LDL-C | pJIA(RF+) | -4.73E-01 | 1.16E+00 | 6.90E-01 | 9.92E-01 |
| HDL-C | sJIA | 1.09E+00 | 6.87E-01 | 1.28E-01 | 3.65E-01 |
| HDL-C | pJIA(RF+) | 1.66E+00 | 1.04E+00 | 1.31E-01 | 5.16E-01 |
| HDL-C | pJIA(RF-) | 1.91E+00 | 1.18E+00 | 1.25E-01 | 6.09E-01 |
| HDL-C | oJIA | -4.13E-01 | 8.18E-01 | 6.20E-01 | 7.59E-01 |
| Total triglycerides | sJIA | 9.80E-01 | 7.49E-01 | 2.04E-01 | 4.35E-01 |
| Total triglycerides | oJIA | -6.19E-01 | 8.09E-01 | 4.55E-01 | 6.09E-01 |
| Total triglycerides | pJIA(RF-) | -1.13E+00 | 1.25E+00 | 3.82E-01 | 7.45E-01 |
| Total triglycerides | pJIA(RF+) | 5.76E-01 | 1.13E+00 | 6.17E-01 | 9.92E-01 |
| VLDL-TG | oJIA | -6.31E-01 | 8.21E-01 | 4.53E-01 | 6.09E-01 |
| VLDL-TG | sJIA | 5.56E-01 | 7.64E-01 | 4.75E-01 | 7.38E-01 |
| VLDL-TG | pJIA(RF-) | -8.80E-01 | 1.27E+00 | 5.01E-01 | 7.52E-01 |
| VLDL-TG | pJIA(RF+) | 3.84E-01 | 1.15E+00 | 7.44E-01 | 9.92E-01 |
| LDL-TG | sJIA | 1.90E+00 | 6.64E-01 | 8.96E-03 | 2.31E-01 |
| LDL-TG | pJIA(RF-) | -1.95E+00 | 1.11E+00 | 9.81E-02 | 6.09E-01 |
| LDL-TG | oJIA | -4.03E-01 | 7.28E-01 | 5.87E-01 | 7.44E-01 |
| LDL-TG | pJIA(RF+) | 5.06E-01 | 1.01E+00 | 6.24E-01 | 9.92E-01 |
| HDL-TG | sJIA | 2.15E+00 | 6.22E-01 | 2.24E-03 | 1.59E-01 |
| HDL-TG | pJIA(RF+) | 1.92E+00 | 9.72E-01 | 6.72E-02 | 2.98E-01 |
| HDL-TG | pJIA(RF-) | -8.03E-01 | 1.14E+00 | 4.92E-01 | 7.52E-01 |
| HDL-TG | oJIA | 9.52E-02 | 8.00E-01 | 9.07E-01 | 9.38E-01 |
| Total-PL | sJIA | 1.30E+00 | 7.07E-01 | 7.90E-02 | 2.95E-01 |
| Total-PL | oJIA | -1.29E+00 | 7.67E-01 | 1.10E-01 | 4.59E-01 |
| Total-PL | pJIA(RF+) | 8.56E-01 | 1.14E+00 | 4.66E-01 | 9.92E-01 |
| Total-PL | pJIA(RF-) | 1.21E-02 | 1.29E+00 | 9.93E-01 | 9.93E-01 |
| VLDL-PL | oJIA | -1.35E+00 | 7.24E-01 | 7.97E-02 | 4.35E-01 |
| VLDL-PL | pJIA(RF-) | -1.82E+00 | 1.21E+00 | 1.53E-01 | 6.09E-01 |
| VLDL-PL | sJIA | 3.11E-01 | 7.74E-01 | 6.92E-01 | 8.85E-01 |
| VLDL-PL | pJIA(RF+) | -2.55E-01 | 1.12E+00 | 8.23E-01 | 9.92E-01 |
| LDL-PL | oJIA | -9.54E-01 | 7.98E-01 | 2.48E-01 | 4.72E-01 |
| LDL-PL | pJIA(RF-) | -1.01E+00 | 1.26E+00 | 4.34E-01 | 7.52E-01 |
| LDL-PL | sJIA | 1.13E-01 | 7.76E-01 | 8.85E-01 | 9.30E-01 |
| LDL-PL | pJIA(RF+) | -7.35E-01 | 1.15E+00 | 5.33E-01 | 9.92E-01 |
| HDL-PL | sJIA | 1.48E+00 | 6.31E-01 | 2.88E-02 | 2.49E-01 |
| HDL-PL | pJIA(RF+) | 2.00E+00 | 1.01E+00 | 6.57E-02 | 2.98E-01 |
| HDL-PL | oJIA | -8.41E-01 | 7.90E-01 | 3.02E-01 | 4.72E-01 |
| HDL-PL | pJIA(RF-) | 1.59E+00 | 1.18E+00 | 1.97E-01 | 6.09E-01 |
| Total-CE | oJIA | -1.05E+00 | 7.96E-01 | 2.04E-01 | 4.72E-01 |
| Total-CE | sJIA | 6.32E-01 | 7.62E-01 | 4.16E-01 | 6.86E-01 |
| Total-CE | pJIA(RF-) | -2.28E-01 | 1.29E+00 | 8.62E-01 | 9.49E-01 |
| Total-CE | pJIA(RF+) | 1.07E-01 | 1.17E+00 | 9.28E-01 | 9.92E-01 |
| VLDL-CE | oJIA | -1.41E+00 | 7.17E-01 | 6.52E-02 | 4.00E-01 |
| VLDL-CE | pJIA(RF-) | -1.85E+00 | 1.21E+00 | 1.46E-01 | 6.09E-01 |
| VLDL-CE | sJIA | 2.63E-01 | 7.77E-01 | 7.38E-01 | 8.89E-01 |
| VLDL-CE | pJIA(RF+) | -3.27E-01 | 1.13E+00 | 7.77E-01 | 9.92E-01 |
| LDL-CE | oJIA | -9.47E-01 | 8.01E-01 | 2.54E-01 | 4.72E-01 |
| LDL-CE | pJIA(RF-) | -8.42E-01 | 1.27E+00 | 5.19E-01 | 7.52E-01 |
| LDL-CE | sJIA | 2.00E-01 | 7.79E-01 | 7.99E-01 | 9.01E-01 |
| LDL-CE | pJIA(RF+) | -4.39E-01 | 1.16E+00 | 7.11E-01 | 9.92E-01 |
| HDL-CE | sJIA | 9.74E-01 | 6.96E-01 | 1.76E-01 | 4.35E-01 |
| HDL-CE | pJIA(RF+) | 1.54E+00 | 1.04E+00 | 1.60E-01 | 5.44E-01 |
| HDL-CE | pJIA(RF-) | 1.94E+00 | 1.17E+00 | 1.19E-01 | 6.09E-01 |
| HDL-CE | oJIA | -3.77E-01 | 8.16E-01 | 6.50E-01 | 7.69E-01 |
| Total-FC | oJIA | -1.15E+00 | 7.78E-01 | 1.59E-01 | 4.69E-01 |
| Total-FC | sJIA | 7.20E-01 | 7.65E-01 | 3.57E-01 | 6.18E-01 |
| Total-FC | pJIA(RF-) | -5.42E-01 | 1.29E+00 | 6.81E-01 | 8.65E-01 |
| Total-FC | pJIA(RF+) | 1.52E-01 | 1.17E+00 | 8.98E-01 | 9.92E-01 |
| VLDL-FC | oJIA | -1.46E+00 | 7.11E-01 | 5.64E-02 | 4.00E-01 |
| VLDL-FC | pJIA(RF-) | -1.88E+00 | 1.21E+00 | 1.40E-01 | 6.09E-01 |
| VLDL-FC | sJIA | 2.41E-01 | 7.74E-01 | 7.58E-01 | 8.89E-01 |
| VLDL-FC | pJIA(RF+) | -4.68E-01 | 1.13E+00 | 6.84E-01 | 9.92E-01 |
| LDL-FC | oJIA | -8.57E-01 | 8.11E-01 | 3.06E-01 | 4.72E-01 |
| LDL-FC | pJIA(RF-) | -6.70E-01 | 1.27E+00 | 6.07E-01 | 8.42E-01 |
| LDL-FC | sJIA | 8.15E-02 | 7.77E-01 | 9.17E-01 | 9.30E-01 |
| LDL-FC | pJIA(RF+) | -5.41E-01 | 1.16E+00 | 6.48E-01 | 9.92E-01 |
| HDL-FC | sJIA | 1.45E+00 | 6.56E-01 | 3.81E-02 | 2.49E-01 |
| HDL-FC | pJIA(RF+) | 2.03E+00 | 1.02E+00 | 6.56E-02 | 2.98E-01 |
| HDL-FC | pJIA(RF-) | 1.76E+00 | 1.19E+00 | 1.58E-01 | 6.09E-01 |
| HDL-FC | oJIA | -5.35E-01 | 8.22E-01 | 5.24E-01 | 6.77E-01 |
| Total-L | sJIA | 1.12E+00 | 7.39E-01 | 1.45E-01 | 3.96E-01 |
| Total-L | oJIA | -1.24E+00 | 7.69E-01 | 1.26E-01 | 4.64E-01 |
| Total-L | pJIA(RF-) | -4.77E-01 | 1.29E+00 | 7.17E-01 | 8.78E-01 |
| Total-L | pJIA(RF+) | 4.09E-01 | 1.16E+00 | 7.30E-01 | 9.92E-01 |
| VLDL-L | oJIA | -1.21E+00 | 7.55E-01 | 1.27E-01 | 4.64E-01 |
| VLDL-L | pJIA(RF-) | -1.60E+00 | 1.23E+00 | 2.13E-01 | 6.22E-01 |
| VLDL-L | sJIA | 4.50E-01 | 7.71E-01 | 5.66E-01 | 7.88E-01 |
| VLDL-L | pJIA(RF+) | -2.66E-02 | 1.14E+00 | 9.82E-01 | 9.92E-01 |
| LDL-L | oJIA | -9.26E-01 | 8.01E-01 | 2.64E-01 | 4.72E-01 |
| LDL-L | pJIA(RF-) | -8.95E-01 | 1.27E+00 | 4.92E-01 | 7.52E-01 |
| LDL-L | sJIA | 2.18E-01 | 7.77E-01 | 7.82E-01 | 8.95E-01 |
| LDL-L | pJIA(RF+) | -5.11E-01 | 1.16E+00 | 6.66E-01 | 9.92E-01 |
| HDL-L | sJIA | 1.40E+00 | 6.49E-01 | 4.15E-02 | 2.49E-01 |
| HDL-L | pJIA(RF+) | 1.94E+00 | 1.02E+00 | 7.66E-02 | 3.20E-01 |
| HDL-L | oJIA | -6.34E-01 | 8.08E-01 | 4.44E-01 | 6.09E-01 |
| HDL-L | pJIA(RF-) | 1.73E+00 | 1.18E+00 | 1.63E-01 | 6.09E-01 |
| Total-P | sJIA | 9.24E-01 | 7.13E-01 | 2.08E-01 | 4.35E-01 |
| Total-P | oJIA | -8.66E-01 | 8.02E-01 | 2.96E-01 | 4.72E-01 |
| Total-P | pJIA(RF-) | 6.42E-01 | 1.25E+00 | 6.16E-01 | 8.42E-01 |
| Total-P | pJIA(RF+) | 5.79E-01 | 1.12E+00 | 6.12E-01 | 9.92E-01 |
| VLDL-P | oJIA | -1.25E+00 | 7.29E-01 | 1.06E-01 | 4.59E-01 |
| VLDL-P | pJIA(RF-) | -1.86E+00 | 1.20E+00 | 1.42E-01 | 6.09E-01 |
| VLDL-P | sJIA | 6.20E-01 | 7.68E-01 | 4.28E-01 | 6.91E-01 |
| VLDL-P | pJIA(RF+) | -1.20E-02 | 1.12E+00 | 9.92E-01 | 9.92E-01 |
| LDL-P | oJIA | -1.08E+00 | 7.75E-01 | 1.81E-01 | 4.72E-01 |
| LDL-P | pJIA(RF-) | -1.19E+00 | 1.26E+00 | 3.61E-01 | 7.32E-01 |
| LDL-P | sJIA | 1.07E-01 | 7.80E-01 | 8.93E-01 | 9.30E-01 |
| LDL-P | pJIA(RF+) | -6.39E-01 | 1.16E+00 | 5.89E-01 | 9.92E-01 |
| HDL-P | sJIA | 9.41E-01 | 7.03E-01 | 1.94E-01 | 4.35E-01 |
| HDL-P | oJIA | -7.51E-01 | 8.04E-01 | 3.63E-01 | 5.49E-01 |
| HDL-P | pJIA(RF-) | 8.81E-01 | 1.24E+00 | 4.87E-01 | 7.52E-01 |
| HDL-P | pJIA(RF+) | 7.22E-01 | 1.10E+00 | 5.23E-01 | 9.92E-01 |
| VLDL particle size | oJIA | -5.94E-01 | 8.30E-01 | 4.84E-01 | 6.36E-01 |
| VLDL particle size | pJIA(RF-) | -8.50E-01 | 1.28E+00 | 5.15E-01 | 7.52E-01 |
| VLDL particle size | sJIA | -3.15E-01 | 7.65E-01 | 6.84E-01 | 8.85E-01 |
| VLDL particle size | pJIA(RF+) | -6.24E-01 | 1.16E+00 | 5.98E-01 | 9.92E-01 |
| LDL particle size | sJIA | -4.71E-01 | 7.59E-01 | 5.41E-01 | 7.84E-01 |
| LDL particle size | oJIA | -2.92E-01 | 8.09E-01 | 7.22E-01 | 8.14E-01 |
| LDL particle size | pJIA(RF-) | 5.69E-01 | 1.24E+00 | 6.54E-01 | 8.60E-01 |
| LDL particle size | pJIA(RF+) | 4.66E-01 | 1.12E+00 | 6.84E-01 | 9.92E-01 |
| HDL particle size | pJIA(RF+) | 3.01E+00 | 8.50E-01 | 2.95E-03 | 5.24E-02 |
| HDL particle size | sJIA | 1.52E+00 | 6.42E-01 | 2.72E-02 | 2.49E-01 |
| HDL particle size | pJIA(RF-) | 2.72E+00 | 9.70E-01 | 1.33E-02 | 3.16E-01 |
| HDL particle size | oJIA | 6.39E-01 | 8.20E-01 | 4.47E-01 | 6.09E-01 |
| Phosphoglycerides | sJIA | 1.42E+00 | 6.81E-01 | 4.83E-02 | 2.49E-01 |
| Phosphoglycerides | oJIA | -1.18E+00 | 7.73E-01 | 1.46E-01 | 4.69E-01 |
| Phosphoglycerides | pJIA(RF+) | 1.21E+00 | 1.12E+00 | 2.97E-01 | 7.82E-01 |
| Phosphoglycerides | pJIA(RF-) | 1.79E-01 | 1.28E+00 | 8.90E-01 | 9.58E-01 |
| TG/PG | pJIA(RF-) | -1.10E+00 | 1.26E+00 | 3.95E-01 | 7.45E-01 |
| TG/PG | sJIA | 2.88E-01 | 7.66E-01 | 7.11E-01 | 8.85E-01 |
| TG/PG | oJIA | -2.30E-02 | 8.34E-01 | 9.78E-01 | 9.78E-01 |
| TG/PG | pJIA(RF+) | -3.91E-02 | 1.14E+00 | 9.73E-01 | 9.92E-01 |
| Total cholines | sJIA | 1.36E+00 | 6.93E-01 | 6.21E-02 | 2.49E-01 |
| Total cholines | oJIA | -1.12E+00 | 7.83E-01 | 1.72E-01 | 4.69E-01 |
| Total cholines | pJIA(RF+) | 1.10E+00 | 1.13E+00 | 3.44E-01 | 8.71E-01 |
| Total cholines | pJIA(RF-) | 2.68E-01 | 1.28E+00 | 8.37E-01 | 9.49E-01 |
| Phosphatidylcholines | sJIA | 1.53E+00 | 6.65E-01 | 3.19E-02 | 2.49E-01 |
| Phosphatidylcholines | oJIA | -9.03E-01 | 7.93E-01 | 2.70E-01 | 4.72E-01 |
| Phosphatidylcholines | pJIA(RF+) | 1.38E+00 | 1.11E+00 | 2.32E-01 | 6.38E-01 |
| Phosphatidylcholines | pJIA(RF-) | 4.04E-01 | 1.26E+00 | 7.53E-01 | 8.98E-01 |
| Sphingomyelins | sJIA | 1.07E+00 | 7.44E-01 | 1.65E-01 | 4.34E-01 |
| Sphingomyelins | oJIA | -1.15E+00 | 7.85E-01 | 1.60E-01 | 4.69E-01 |
| Sphingomyelins | pJIA(RF-) | -5.31E-01 | 1.28E+00 | 6.83E-01 | 8.65E-01 |
| Sphingomyelins | pJIA(RF+) | 3.64E-01 | 1.16E+00 | 7.58E-01 | 9.92E-01 |
| ApoB | oJIA | -1.12E+00 | 7.69E-01 | 1.63E-01 | 4.69E-01 |
| ApoB | pJIA(RF-) | -1.26E+00 | 1.26E+00 | 3.33E-01 | 7.17E-01 |
| ApoB | sJIA | 1.47E-01 | 7.80E-01 | 8.52E-01 | 9.17E-01 |
| ApoB | pJIA(RF+) | -5.62E-01 | 1.15E+00 | 6.33E-01 | 9.92E-01 |
| ApoA1 | sJIA | 1.32E+00 | 6.63E-01 | 5.86E-02 | 2.49E-01 |
| ApoA1 | pJIA(RF+) | 1.54E+00 | 1.06E+00 | 1.66E-01 | 5.44E-01 |
| ApoA1 | oJIA | -6.39E-01 | 8.09E-01 | 4.40E-01 | 6.09E-01 |
| ApoA1 | pJIA(RF-) | 1.41E+00 | 1.21E+00 | 2.65E-01 | 6.88E-01 |
| ApoB/ApoA1 | oJIA | -8.59E-01 | 7.71E-01 | 2.81E-01 | 4.72E-01 |
| ApoB/ApoA1 | pJIA(RF-) | -2.00E+00 | 1.18E+00 | 1.10E-01 | 6.09E-01 |
| ApoB/ApoA1 | sJIA | -6.97E-01 | 7.27E-01 | 3.48E-01 | 6.18E-01 |
| ApoB/ApoA1 | pJIA(RF+) | -1.40E+00 | 1.08E+00 | 2.13E-01 | 6.29E-01 |
| Total fatty acids | sJIA | 1.40E+00 | 7.17E-01 | 6.31E-02 | 2.49E-01 |
| Total fatty acids | oJIA | -1.65E+00 | 7.00E-01 | 3.07E-02 | 4.00E-01 |
| Total fatty acids | pJIA(RF-) | -1.27E+00 | 1.25E+00 | 3.29E-01 | 7.17E-01 |
| Total fatty acids | pJIA(RF+) | 2.44E-01 | 1.16E+00 | 8.36E-01 | 9.92E-01 |
| Unsaturation | pJIA(RF+) | 2.23E+00 | 7.73E-01 | 1.14E-02 | 7.35E-02 |
| Unsaturation | pJIA(RF-) | 3.14E+00 | 9.30E-01 | 4.13E-03 | 2.93E-01 |
| Unsaturation | sJIA | -8.96E-01 | 7.29E-01 | 2.32E-01 | 4.71E-01 |
| Unsaturation | oJIA | -8.69E-01 | 7.16E-01 | 2.42E-01 | 4.72E-01 |
| Omega-3 | pJIA(RF+) | 2.66E+00 | 8.65E-01 | 7.76E-03 | 6.47E-02 |
| Omega-3 | sJIA | 1.42E+00 | 7.18E-01 | 6.01E-02 | 2.49E-01 |
| Omega-3 | oJIA | -9.54E-01 | 8.06E-01 | 2.53E-01 | 4.72E-01 |
| Omega-3 | pJIA(RF-) | 1.35E+00 | 1.25E+00 | 2.97E-01 | 7.17E-01 |
| Omega-6 | oJIA | -1.48E+00 | 7.39E-01 | 6.12E-02 | 4.00E-01 |
| Omega-6 | sJIA | 8.75E-01 | 7.38E-01 | 2.49E-01 | 4.78E-01 |
| Omega-6 | pJIA(RF-) | -6.90E-02 | 1.28E+00 | 9.58E-01 | 9.71E-01 |
| Omega-6 | pJIA(RF+) | -1.95E-01 | 1.15E+00 | 8.67E-01 | 9.92E-01 |
| PUFA | oJIA | -1.54E+00 | 7.34E-01 | 5.11E-02 | 4.00E-01 |
| PUFA | sJIA | 1.01E+00 | 7.34E-01 | 1.84E-01 | 4.35E-01 |
| PUFA | pJIA(RF-) | 9.22E-02 | 1.28E+00 | 9.44E-01 | 9.71E-01 |
| PUFA | pJIA(RF+) | 5.41E-01 | 1.15E+00 | 6.45E-01 | 9.92E-01 |
| MUFA | sJIA | 1.44E+00 | 7.20E-01 | 5.74E-02 | 2.49E-01 |
| MUFA | oJIA | -1.24E+00 | 7.34E-01 | 1.10E-01 | 4.59E-01 |
| MUFA | pJIA(RF-) | -2.19E+00 | 1.17E+00 | 8.17E-02 | 6.09E-01 |
| MUFA | pJIA(RF+) | -1.52E-01 | 1.12E+00 | 8.94E-01 | 9.92E-01 |
| SFA | sJIA | 1.51E+00 | 7.05E-01 | 4.30E-02 | 2.49E-01 |
| SFA | oJIA | -1.87E+00 | 6.62E-01 | 1.18E-02 | 3.77E-01 |
| SFA | pJIA(RF-) | -1.66E+00 | 1.22E+00 | 1.93E-01 | 6.09E-01 |
| SFA | pJIA(RF+) | 2.29E-01 | 1.16E+00 | 8.47E-01 | 9.92E-01 |
| LA | sJIA | 1.01E+00 | 7.25E-01 | 1.79E-01 | 4.35E-01 |
| LA | oJIA | -1.04E+00 | 7.70E-01 | 1.93E-01 | 4.72E-01 |
| LA | pJIA(RF-) | 2.11E-01 | 1.26E+00 | 8.69E-01 | 9.49E-01 |
| LA | pJIA(RF+) | -4.82E-02 | 1.14E+00 | 9.67E-01 | 9.92E-01 |
| DHA | pJIA(RF+) | 2.53E+00 | 8.96E-01 | 1.29E-02 | 7.63E-02 |
| DHA | sJIA | 1.51E+00 | 7.09E-01 | 4.45E-02 | 2.49E-01 |
| DHA | pJIA(RF-) | 1.69E+00 | 1.22E+00 | 1.86E-01 | 6.09E-01 |
| DHA | oJIA | 3.67E-01 | 8.30E-01 | 6.64E-01 | 7.72E-01 |
| PUFA/MUFA | pJIA(RF-) | 3.09E+00 | 1.02E+00 | 8.27E-03 | 2.94E-01 |
| PUFA/MUFA | sJIA | -1.24E+00 | 7.28E-01 | 1.03E-01 | 3.47E-01 |
| PUFA/MUFA | oJIA | 4.13E-01 | 7.76E-01 | 6.02E-01 | 7.49E-01 |
| PUFA/MUFA | pJIA(RF+) | 7.46E-01 | 1.04E+00 | 4.84E-01 | 9.92E-01 |
| Omega-6/Omega-3 | pJIA(RF+) | -2.70E+00 | 8.32E-01 | 5.37E-03 | 5.45E-02 |
| Omega-6/Omega-3 | sJIA | -1.20E+00 | 7.29E-01 | 1.14E-01 | 3.65E-01 |
| Omega-6/Omega-3 | pJIA(RF-) | -1.41E+00 | 1.23E+00 | 2.71E-01 | 6.88E-01 |
| Omega-6/Omega-3 | oJIA | 3.25E-01 | 8.24E-01 | 6.98E-01 | 7.99E-01 |
| Alanine | sJIA | 1.68E+00 | 5.99E-01 | 1.05E-02 | 2.31E-01 |
| Alanine | pJIA(RF+) | 2.10E+00 | 1.04E+00 | 6.04E-02 | 2.98E-01 |
| Alanine | oJIA | 1.89E+00 | 6.73E-01 | 1.22E-02 | 3.77E-01 |
| Alanine | pJIA(RF-) | 1.54E+00 | 1.14E+00 | 1.95E-01 | 6.09E-01 |
| Glutamine | oJIA | 9.87E-01 | 8.06E-01 | 2.38E-01 | 4.72E-01 |
| Glutamine | sJIA | 4.45E-01 | 7.58E-01 | 5.64E-01 | 7.88E-01 |
| Glutamine | pJIA(RF-) | 5.44E-01 | 1.36E+00 | 6.95E-01 | 8.65E-01 |
| Glutamine | pJIA(RF+) | 8.25E-02 | 1.16E+00 | 9.44E-01 | 9.92E-01 |
| Glycine | oJIA | 1.48E+00 | 7.56E-01 | 6.77E-02 | 4.00E-01 |
| Glycine | pJIA(RF+) | 1.50E+00 | 1.05E+00 | 1.74E-01 | 5.44E-01 |
| Glycine | sJIA | 5.12E-01 | 7.47E-01 | 5.00E-01 | 7.56E-01 |
| Glycine | pJIA(RF-) | 3.73E-01 | 1.19E+00 | 7.59E-01 | 8.98E-01 |
| Histidine | pJIA(RF+) | 3.47E+00 | 7.49E-01 | 3.25E-04 | 1.15E-02 |
| Histidine | oJIA | 1.76E+00 | 6.59E-01 | 1.59E-02 | 3.77E-01 |
| Histidine | sJIA | -3.24E-02 | 7.82E-01 | 9.67E-01 | 9.67E-01 |
| Histidine | pJIA(RF-) | 7.32E-02 | 1.16E+00 | 9.50E-01 | 9.71E-01 |
| Total BCAA | pJIA(RF+) | 2.74E+00 | 8.43E-01 | 5.36E-03 | 5.45E-02 |
| Total BCAA | pJIA(RF-) | -1.12E+00 | 1.12E+00 | 3.33E-01 | 7.17E-01 |
| Total BCAA | oJIA | -1.56E-01 | 7.29E-01 | 8.34E-01 | 8.98E-01 |
| Total BCAA | sJIA | 1.67E-01 | 6.96E-01 | 8.13E-01 | 9.02E-01 |
| Isoleucine | pJIA(RF+) | 3.11E+00 | 7.86E-01 | 1.27E-03 | 3.01E-02 |
| Isoleucine | pJIA(RF-) | -1.54E+00 | 1.10E+00 | 1.81E-01 | 6.09E-01 |
| Isoleucine | sJIA | 4.58E-01 | 6.35E-01 | 4.78E-01 | 7.38E-01 |
| Isoleucine | oJIA | -2.44E-01 | 7.40E-01 | 7.45E-01 | 8.27E-01 |
| Leucine | pJIA(RF+) | 2.52E+00 | 8.27E-01 | 8.21E-03 | 6.47E-02 |
| Leucine | pJIA(RF-) | -1.30E+00 | 1.02E+00 | 2.19E-01 | 6.22E-01 |
| Leucine | sJIA | -2.60E-01 | 6.77E-01 | 7.04E-01 | 8.85E-01 |
| Leucine | oJIA | 1.48E-01 | 6.96E-01 | 8.35E-01 | 8.98E-01 |
| Valine | pJIA(RF+) | 2.62E+00 | 8.95E-01 | 1.05E-02 | 7.35E-02 |
| Valine | pJIA(RF-) | -8.32E-01 | 1.18E+00 | 4.93E-01 | 7.52E-01 |
| Valine | oJIA | -3.54E-01 | 7.53E-01 | 6.44E-01 | 7.69E-01 |
| Valine | sJIA | 3.16E-01 | 7.24E-01 | 6.67E-01 | 8.85E-01 |
| Phenylalanine | pJIA(RF+) | 3.43E+00 | 6.41E-01 | 8.04E-05 | 5.71E-03 |
| Phenylalanine | sJIA | 7.63E-01 | 5.75E-01 | 1.98E-01 | 4.35E-01 |
| Phenylalanine | oJIA | 7.14E-01 | 6.34E-01 | 2.76E-01 | 4.72E-01 |
| Phenylalanine | pJIA(RF-) | 1.94E+00 | 1.10E+00 | 9.87E-02 | 6.09E-01 |
| Tyrosine | pJIA(RF+) | 2.99E+00 | 8.75E-01 | 3.79E-03 | 5.38E-02 |
| Tyrosine | sJIA | 1.18E+00 | 6.85E-01 | 9.78E-02 | 3.47E-01 |
| Tyrosine | oJIA | 1.34E+00 | 6.60E-01 | 5.83E-02 | 4.00E-01 |
| Tyrosine | pJIA(RF-) | -1.30E-01 | 1.25E+00 | 9.18E-01 | 9.71E-01 |
| Pyruvate | sJIA | 1.15E+00 | 7.18E-01 | 1.26E-01 | 3.65E-01 |
| Pyruvate | oJIA | -8.16E-02 | 8.48E-01 | 9.25E-01 | 9.38E-01 |
| Pyruvate | pJIA(RF-) | 2.41E-01 | 1.34E+00 | 8.60E-01 | 9.49E-01 |
| Pyruvate | pJIA(RF+) | -1.09E-01 | 1.16E+00 | 9.26E-01 | 9.92E-01 |
| Citrate | sJIA | -8.57E-01 | 7.35E-01 | 2.56E-01 | 4.78E-01 |
| Citrate | pJIA(RF+) | 1.53E+00 | 1.08E+00 | 1.76E-01 | 5.44E-01 |
| Citrate | pJIA(RF-) | 8.55E-01 | 1.27E+00 | 5.11E-01 | 7.52E-01 |
| Citrate | oJIA | 8.10E-02 | 8.37E-01 | 9.24E-01 | 9.38E-01 |
| Acetate | sJIA | -1.13E+00 | 7.08E-01 | 1.26E-01 | 3.65E-01 |
| Acetate | oJIA | -5.42E-01 | 6.47E-01 | 4.13E-01 | 6.09E-01 |
| Acetate | pJIA(RF+) | -1.10E+00 | 8.82E-01 | 2.34E-01 | 6.38E-01 |
| Acetate | pJIA(RF-) | -4.67E-01 | 1.02E+00 | 6.54E-01 | 8.60E-01 |
| Acetoacetate | sJIA | -1.70E+00 | 6.31E-01 | 1.30E-02 | 2.31E-01 |
| Acetoacetate | oJIA | -1.51E+00 | 6.76E-01 | 3.88E-02 | 4.00E-01 |
| Acetoacetate | pJIA(RF-) | -1.26E+00 | 1.19E+00 | 3.06E-01 | 7.17E-01 |
| Acetoacetate | pJIA(RF+) | -9.94E-01 | 1.12E+00 | 3.91E-01 | 9.25E-01 |
| Acetone | sJIA | -1.44E+00 | 6.65E-01 | 4.09E-02 | 2.49E-01 |
| Acetone | oJIA | -1.20E+00 | 7.57E-01 | 1.31E-01 | 4.64E-01 |
| Acetone | pJIA(RF-) | -1.06E+00 | 1.22E+00 | 3.99E-01 | 7.45E-01 |
| Acetone | pJIA(RF+) | -1.05E+00 | 1.13E+00 | 3.69E-01 | 9.03E-01 |
| Creatinine | oJIA | -7.65E-01 | 7.01E-01 | 2.91E-01 | 4.72E-01 |
| Creatinine | pJIA(RF-) | -1.80E+00 | 9.54E-01 | 7.86E-02 | 6.09E-01 |
| Creatinine | sJIA | -1.73E-01 | 5.70E-01 | 7.64E-01 | 8.89E-01 |
| Creatinine | pJIA(RF+) | -3.99E-02 | 1.02E+00 | 9.69E-01 | 9.92E-01 |
| Albumin | sJIA | -7.00E-01 | 7.26E-01 | 3.45E-01 | 6.18E-01 |
| Albumin | pJIA(RF-) | 1.43E+00 | 1.19E+00 | 2.48E-01 | 6.78E-01 |
| Albumin | oJIA | 1.19E-01 | 8.36E-01 | 8.89E-01 | 9.38E-01 |
| Albumin | pJIA(RF+) | -1.66E-01 | 1.08E+00 | 8.79E-01 | 9.92E-01 |
| Glycoprotein acetyls | oJIA | -1.02E+00 | 8.00E-01 | 2.21E-01 | 4.72E-01 |
| Glycoprotein acetyls | sJIA | 8.86E-01 | 7.60E-01 | 2.56E-01 | 4.78E-01 |
| Glycoprotein acetyls | pJIA(RF+) | -1.63E+00 | 1.09E+00 | 1.55E-01 | 5.44E-01 |
| Glycoprotein acetyls | pJIA(RF-) | -1.66E+00 | 1.18E+00 | 1.78E-01 | 6.09E-01 |

^a^ Adjusted by BH

**Supplementary Data D9.** The association between GlycA and 70 primary measures from a linear regression model with 70 primary measures as the exposure and GlycA as an outcome adjusting age and sex of participants (n=90)

| Biomarker | estimate | se | P-value | P*_adj_ ^a^* |
| --- | --- | --- | --- | --- |
| Total-C | 4.92E-02 | 1.07E-01 | 6.45E-01 | 8.53E-01 |
| non-HDL-C | 2.11E-04 | 1.07E-01 | 9.98E-01 | 9.98E-01 |
| Remnant-C | 1.73E-02 | 1.07E-01 | 8.72E-01 | 9.55E-01 |
| VLDL-C | 5.19E-02 | 1.07E-01 | 6.30E-01 | 8.53E-01 |
| Clinical LDL-C | -2.30E-02 | 1.06E-01 | 8.28E-01 | 9.40E-01 |
| LDL-C | -1.58E-02 | 1.06E-01 | 8.82E-01 | 9.55E-01 |
| HDL-C | 1.16E-01 | 1.06E-01 | 2.75E-01 | 6.38E-01 |
| Total fatty acids | 3.58E-01 | 1.01E-01 | 6.45E-04 | 5.02E-03 |
| Unsaturation | -5.13E-02 | 1.07E-01 | 6.33E-01 | 8.53E-01 |
| Omega-3 | 2.28E-01 | 1.05E-01 | 3.27E-02 | 1.43E-01 |
| Omega-6 | 2.80E-01 | 1.03E-01 | 8.19E-03 | 4.41E-02 |
| PUFA | 2.89E-01 | 1.03E-01 | 6.27E-03 | 3.66E-02 |
| MUFA | 4.06E-01 | 9.88E-02 | 9.20E-05 | 1.07E-03 |
| SFA | 3.29E-01 | 1.02E-01 | 1.80E-03 | 1.26E-02 |
| LA | 2.29E-01 | 1.05E-01 | 3.20E-02 | 1.43E-01 |
| DHA | 1.76E-01 | 1.06E-01 | 9.93E-02 | 3.86E-01 |
| PUFA/MUFA | -3.65E-01 | 9.96E-02 | 4.31E-04 | 3.77E-03 |
| Omega-6/Omega-3 | -1.60E-01 | 1.06E-01 | 1.37E-01 | 4.18E-01 |
| Alanine | -8.77E-02 | 9.48E-02 | 3.58E-01 | 7.36E-01 |
| Glutamine | -4.61E-01 | 9.46E-02 | 5.16E-06 | 9.02E-05 |
| Glycine | -2.06E-01 | 1.05E-01 | 5.35E-02 | 2.20E-01 |
| Histidine | -5.92E-01 | 8.20E-02 | 1.95E-10 | 1.36E-08 |
| Total BCAA | 6.38E-03 | 9.85E-02 | 9.48E-01 | 9.76E-01 |
| Isoleucine | 1.24E-02 | 9.82E-02 | 9.00E-01 | 9.55E-01 |
| Leucine | -8.74E-02 | 9.67E-02 | 3.68E-01 | 7.36E-01 |
| Valine | 6.48E-02 | 1.00E-01 | 5.19E-01 | 8.53E-01 |
| Phenylalanine | 2.07E-02 | 9.79E-02 | 8.33E-01 | 9.40E-01 |
| Tyrosine | 8.33E-02 | 1.05E-01 | 4.31E-01 | 8.15E-01 |
| Phosphoglycerides | 1.62E-01 | 1.06E-01 | 1.30E-01 | 4.18E-01 |
| TG/PG | 6.07E-02 | 1.07E-01 | 5.71E-01 | 8.53E-01 |
| Total cholines | 1.62E-01 | 1.06E-01 | 1.29E-01 | 4.18E-01 |
| Phosphatidylcholines | 4.38E-02 | 1.07E-01 | 6.83E-01 | 8.69E-01 |
| Sphingomyelins | 4.18E-01 | 9.68E-02 | 4.24E-05 | 5.94E-04 |
| Total triglycerides | 1.21E-01 | 1.07E-01 | 2.59E-01 | 6.26E-01 |
| VLDL-TG | 6.21E-02 | 1.07E-01 | 5.65E-01 | 8.53E-01 |
| LDL-TG | 3.95E-01 | 9.90E-02 | 1.40E-04 | 1.40E-03 |
| HDL-TG | 2.48E-02 | 1.07E-01 | 8.18E-01 | 9.40E-01 |
| Total-PL | 1.43E-01 | 1.06E-01 | 1.84E-01 | 5.11E-01 |
| VLDL-PL | 6.89E-02 | 1.08E-01 | 5.24E-01 | 8.53E-01 |
| LDL-PL | 1.02E-02 | 1.06E-01 | 9.23E-01 | 9.65E-01 |
| HDL-PL | 1.43E-01 | 1.05E-01 | 1.76E-01 | 5.11E-01 |
| Total-CE | 4.65E-02 | 1.06E-01 | 6.63E-01 | 8.59E-01 |
| VLDL-CE | 3.83E-02 | 1.07E-01 | 7.22E-01 | 8.86E-01 |
| LDL-CE | -3.71E-03 | 1.07E-01 | 9.72E-01 | 9.86E-01 |
| HDL-CE | 1.02E-01 | 1.06E-01 | 3.39E-01 | 7.36E-01 |
| Total-FC | 5.44E-02 | 1.07E-01 | 6.13E-01 | 8.53E-01 |
| VLDL-FC | 6.39E-02 | 1.08E-01 | 5.54E-01 | 8.53E-01 |
| LDL-FC | -5.25E-02 | 1.05E-01 | 6.19E-01 | 8.53E-01 |
| HDL-FC | 1.61E-01 | 1.06E-01 | 1.32E-01 | 4.18E-01 |
| Total-L | 1.16E-01 | 1.07E-01 | 2.83E-01 | 6.38E-01 |
| VLDL-L | 6.74E-02 | 1.08E-01 | 5.33E-01 | 8.53E-01 |
| LDL-L | 1.52E-02 | 1.06E-01 | 8.86E-01 | 9.55E-01 |
| HDL-L | 1.35E-01 | 1.05E-01 | 2.04E-01 | 5.11E-01 |
| Total-P | 7.29E-02 | 1.05E-01 | 4.91E-01 | 8.53E-01 |
| VLDL-P | 8.94E-02 | 1.07E-01 | 4.08E-01 | 7.93E-01 |
| LDL-P | 4.91E-02 | 1.06E-01 | 6.46E-01 | 8.53E-01 |
| HDL-P | 6.94E-02 | 1.05E-01 | 5.11E-01 | 8.53E-01 |
| VLDL particle size | -2.48E-02 | 1.08E-01 | 8.18E-01 | 9.40E-01 |
| LDL particle size | 5.93E-02 | 1.05E-01 | 5.74E-01 | 8.53E-01 |
| HDL particle size | 1.38E-01 | 1.06E-01 | 1.98E-01 | 5.11E-01 |
| ApoB | 3.83E-02 | 1.07E-01 | 7.20E-01 | 8.86E-01 |
| ApoA1 | 9.66E-02 | 1.05E-01 | 3.61E-01 | 7.36E-01 |
| ApoB/ApoA1 | -2.37E-02 | 1.05E-01 | 8.22E-01 | 9.40E-01 |
| Pyruvate | 2.78E-01 | 1.09E-01 | 1.29E-02 | 6.44E-02 |
| Citrate | -5.63E-01 | 8.92E-02 | 1.15E-08 | 2.68E-07 |
| Acetate | -2.91E-01 | 9.40E-02 | 2.65E-03 | 1.69E-02 |
| Acetoacetate | 1.31E-01 | 9.94E-02 | 1.90E-01 | 5.11E-01 |
| Acetone | 6.47E-02 | 1.01E-01 | 5.25E-01 | 8.53E-01 |
| Creatinine | 1.28E-01 | 7.88E-02 | 1.07E-01 | 3.94E-01 |
| Albumin | -5.99E-01 | 8.56E-02 | 5.31E-10 | 1.86E-08 |

^a^ Adjusted by BH

**Supplementary Data D10.** The association between hsCRP and 70 primary measures from a linear regression model with 70 primary measures as the exposure and hsCRP as an outcome adjusting age and sex of participants (n=90)

| Biomarker | estimate | se | P-value | P_adj_^a^ |
| --- | --- | --- | --- | --- |
| Total-C | -1.56E-01 | 1.07E-01 | 1.48E-01 | 4.44E-01 |
| non-HDL-C | -1.57E-01 | 1.07E-01 | 1.46E-01 | 4.44E-01 |
| Remnant-C | -1.45E-01 | 1.08E-01 | 1.81E-01 | 4.70E-01 |
| VLDL-C | -8.38E-02 | 1.09E-01 | 4.44E-01 | 7.21E-01 |
| Clinical LDL-C | -1.49E-01 | 1.06E-01 | 1.65E-01 | 4.44E-01 |
| LDL-C | -1.64E-01 | 1.07E-01 | 1.28E-01 | 4.44E-01 |
| HDL-C | -6.50E-02 | 1.08E-01 | 5.49E-01 | 7.39E-01 |
| Total fatty acids | 7.57E-02 | 1.10E-01 | 4.92E-01 | 7.21E-01 |
| Unsaturation | -1.19E-02 | 1.08E-01 | 9.12E-01 | 9.49E-01 |
| Omega-3 | 2.48E-02 | 1.09E-01 | 8.21E-01 | 9.35E-01 |
| Omega-6 | 1.35E-02 | 1.09E-01 | 9.02E-01 | 9.49E-01 |
| PUFA | 1.89E-02 | 1.09E-01 | 8.63E-01 | 9.44E-01 |
| MUFA | 1.54E-01 | 1.09E-01 | 1.60E-01 | 4.44E-01 |
| SFA | 5.10E-02 | 1.10E-01 | 6.43E-01 | 8.04E-01 |
| LA | -3.18E-02 | 1.10E-01 | 7.73E-01 | 9.01E-01 |
| DHA | 4.32E-02 | 1.09E-01 | 6.93E-01 | 8.36E-01 |
| PUFA/MUFA | -2.16E-01 | 1.06E-01 | 4.47E-02 | 3.84E-01 |
| Omega-6/Omega-3 | -2.38E-02 | 1.09E-01 | 8.28E-01 | 9.35E-01 |
| Alanine | -2.14E-01 | 9.39E-02 | 2.52E-02 | 2.83E-01 |
| Glycine | -2.71E-01 | 1.05E-01 | 1.15E-02 | 1.62E-01 |
| Total BCAA | 5.62E-02 | 1.01E-01 | 5.78E-01 | 7.64E-01 |
| Isoleucine | 5.36E-02 | 9.96E-02 | 5.92E-01 | 7.67E-01 |
| Leucine | -1.17E-02 | 9.86E-02 | 9.06E-01 | 9.49E-01 |
| Valine | 1.03E-01 | 1.02E-01 | 3.16E-01 | 5.98E-01 |
| Phenylalanine | 1.50E-01 | 9.82E-02 | 1.31E-01 | 4.44E-01 |
| Tyrosine | 1.55E-01 | 1.06E-01 | 1.49E-01 | 4.44E-01 |
| Phosphoglycerides | -1.12E-01 | 1.08E-01 | 3.05E-01 | 5.98E-01 |
| TG/PG | -1.07E-02 | 1.09E-01 | 9.22E-01 | 9.49E-01 |
| Total cholines | -1.11E-01 | 1.08E-01 | 3.09E-01 | 5.98E-01 |
| Phosphatidylcholines | -2.12E-01 | 1.06E-01 | 4.94E-02 | 3.84E-01 |
| Sphingomyelins | 1.68E-01 | 1.07E-01 | 1.21E-01 | 4.44E-01 |
| Total triglycerides | -5.53E-02 | 1.09E-01 | 6.15E-01 | 7.82E-01 |
| VLDL-TG | -6.67E-02 | 1.09E-01 | 5.43E-01 | 7.39E-01 |
| LDL-TG | 1.37E-01 | 1.08E-01 | 2.10E-01 | 4.89E-01 |
| HDL-TG | -1.99E-01 | 1.07E-01 | 6.67E-02 | 4.38E-01 |
| Total-PL | -1.22E-01 | 1.08E-01 | 2.66E-01 | 5.47E-01 |
| VLDL-PL | -7.53E-02 | 1.09E-01 | 4.93E-01 | 7.21E-01 |
| LDL-PL | -1.27E-01 | 1.07E-01 | 2.37E-01 | 5.03E-01 |
| HDL-PL | -8.17E-02 | 1.07E-01 | 4.49E-01 | 7.21E-01 |
| Total-CE | -1.57E-01 | 1.07E-01 | 1.46E-01 | 4.44E-01 |
| VLDL-CE | -9.02E-02 | 1.09E-01 | 4.08E-01 | 7.14E-01 |
| LDL-CE | -1.59E-01 | 1.07E-01 | 1.42E-01 | 4.44E-01 |
| HDL-CE | -6.95E-02 | 1.08E-01 | 5.21E-01 | 7.29E-01 |
| Total-FC | -1.52E-01 | 1.08E-01 | 1.61E-01 | 4.44E-01 |
| VLDL-FC | -7.53E-02 | 1.09E-01 | 4.93E-01 | 7.21E-01 |
| LDL-FC | -1.76E-01 | 1.05E-01 | 9.96E-02 | 4.44E-01 |
| HDL-FC | -4.85E-02 | 1.09E-01 | 6.57E-01 | 8.07E-01 |
| Total-L | -1.33E-01 | 1.09E-01 | 2.23E-01 | 5.03E-01 |
| VLDL-L | -7.52E-02 | 1.10E-01 | 4.94E-01 | 7.21E-01 |
| LDL-L | -1.41E-01 | 1.07E-01 | 1.90E-01 | 4.76E-01 |
| HDL-L | -8.33E-02 | 1.08E-01 | 4.41E-01 | 7.21E-01 |
| Total-P | -1.63E-01 | 1.06E-01 | 1.27E-01 | 4.44E-01 |
| VLDL-P | -7.16E-02 | 1.09E-01 | 5.14E-01 | 7.29E-01 |
| LDL-P | -7.84E-02 | 1.08E-01 | 4.70E-01 | 7.21E-01 |
| HDL-P | -1.59E-01 | 1.06E-01 | 1.36E-01 | 4.44E-01 |
| VLDL particle size | -1.02E-01 | 1.09E-01 | 3.53E-01 | 6.50E-01 |
| LDL particle size | 1.39E-03 | 1.07E-01 | 9.90E-01 | 9.90E-01 |
| HDL particle size | 3.19E-02 | 1.09E-01 | 7.70E-01 | 9.01E-01 |
| ApoB | -9.72E-02 | 1.08E-01 | 3.70E-01 | 6.65E-01 |
| ApoA1 | -1.35E-01 | 1.07E-01 | 2.09E-01 | 4.89E-01 |
| ApoB/ApoA1 | -7.05E-03 | 1.07E-01 | 9.48E-01 | 9.61E-01 |
| Pyruvate | 2.07E-02 | 1.12E-01 | 8.54E-01 | 9.44E-01 |
| Acetate | -1.77E-01 | 9.83E-02 | 7.53E-02 | 4.39E-01 |
| Acetoacetate | 2.18E-01 | 9.78E-02 | 2.83E-02 | 2.83E-01 |
| Acetone | 1.82E-01 | 9.88E-02 | 6.88E-02 | 4.38E-01 |
| Creatinine | 9.63E-02 | 8.06E-02 | 2.36E-01 | 5.03E-01 |
| Glutamine | -4.97E-01 | 9.09E-02 | 4.68E-07 | 1.64E-05 |
| Histidine | -4.35E-01 | 9.27E-02 | 1.03E-05 | 2.40E-04 |
| Citrate | -4.04E-01 | 9.77E-02 | 8.10E-05 | 1.42E-03 |
| Albumin | -6.55E-01 | 8.06E-02 | 3.20E-12 | 2.24E-10 |

^a^ Adjusted by BH
